# A two-site flexible clamp mechanism for RET-GDNF-GFRα1 assembly reveals both conformational adaptation and strict geometric spacing

**DOI:** 10.1016/j.str.2020.12.012

**Published:** 2021-07-01

**Authors:** Sarah E. Adams, Andrew G. Purkiss, Phillip P. Knowles, Andrea Nans, David C. Briggs, Annabel Borg, Christopher P. Earl, Kerry M. Goodman, Agata Nawrotek, Aaron J. Borg, Pauline B. McIntosh, Francesca M. Houghton, Svend Kjær, Neil Q. McDonald

**Affiliations:** 1 Signalling and Structural Biology Laboratory, Francis Crick Institute, NW1 1AT London, UK; 2 Structural Biology Science Technology Platform, Francis Crick Institute, NW1 1AT London, UK; 3 Mass Spectrometry Science Technology Platform, Francis Crick Institute, NW1 1AT London, UK; 4 Structural Biology of Cells and Viruses Laboratory, The Francis Crick Institute, 1 Midland Road, London NW1 1AT, UK; 5 Institute of Structural and Molecular Biology, Department of Biological Sciences, Birkbeck College, Malet Street, London WC1E 7HX, UK

**Keywords:** ligand recognition, receptor tyrosine kinase, GDNF family ligands, cryo-EM, X-ray crystallography, glycosylation, cystine knot, RET, co-receptor

## Abstract

RET receptor tyrosine kinase plays vital developmental and neuroprotective roles in metazoans. GDNF family ligands (GFLs) when bound to cognate GFRα co-receptors recognize and activate RET stimulating its cytoplasmic kinase function. The principles for RET ligand-co-receptor recognition are incompletely understood. Here, we report a crystal structure of the cadherin-like module (CLD1-4) from zebrafish RET revealing interdomain flexibility between CLD2 and CLD3. Comparison with a cryo-electron microscopy structure of a ligand-engaged zebrafish RET^ECD^-GDNF-GFRα1a complex indicates conformational changes within a clade-specific CLD3 loop adjacent to the co-receptor. Our observations indicate that RET is a molecular clamp with a flexible calcium-dependent arm that adapts to different GFRα co-receptors, while its rigid arm recognizes a GFL dimer to align both membrane-proximal cysteine-rich domains. We also visualize linear arrays of RET^ECD^-GDNF-GFRα1a suggesting that a conserved contact stabilizes higher-order species*.* Our study reveals that ligand-co-receptor recognition by RET involves both receptor plasticity and strict spacing of receptor dimers by GFL ligands.

## Highlights

• X-ray structure of zebrafish RET^CLD^^1-4^ module reveals conformational flexibility
• Conformational differences between RET^CLD1-4^ and a liganded RET^ECD^ cryo-EM structure
• Spatial separation of RET^ECD^ CRD dimer C termini imposed by each ligand dimer
• Differences in GDNF and GDF15 co-receptor engagement of RET and multimerization evidence

## Graphical abstract

**Figure:**
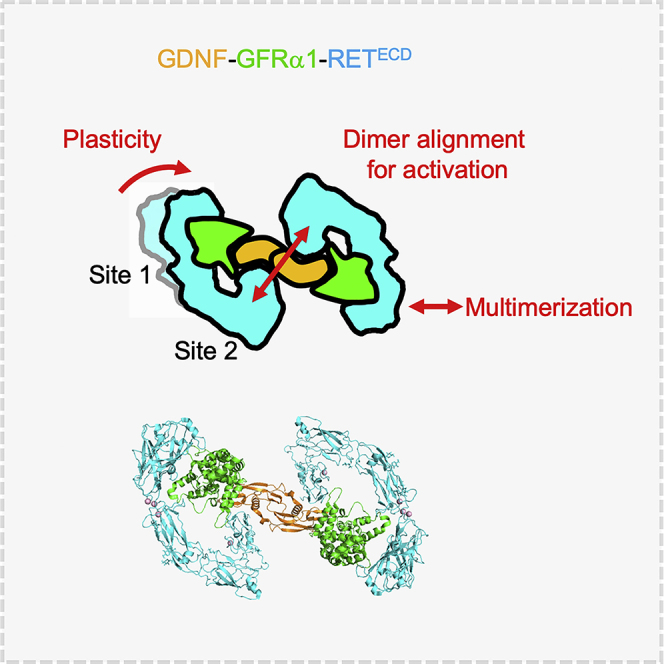

## Teaser

Adams et al. use X-ray crystallography and cryo-electron microscopy to probe conformational changes in RET due to binding of GDNF ligand-GFRα1 co-receptor. The study shows a two-site clamping mechanism with flexible adaptations near the RET calcium sites but near identical spacings of the RET cysteine-rich domains established by GDNF/GDF15 ligand dimers.

## Introduction

Neurotrophic factors fulfill an essential function to support and protect both developing and mature neurons (Henderson et al., 1994). This neuroprotective therapeutic potential has led to an interest in understanding how they engage and activate their cell surface receptors (Airaksinen and Saarma, 2002; Allen et al., 2013). The glial cell line-derived neurotrophic factor (GDNF) family ligands (GFLs) constitutes an important family of neurotrophic factors that include GDNF (Durbec et al., 1996), Neurturin (NRTN) (Kotzbauer et al., 1996), Artemin (ARTN) (Baloh et al., 1998b), Persephin (PSPN) (Airaksinen and Saarma, 2002; Milbrandt et al., 1998), and more recently GDF15 (Emmerson et al., 2017; Hsu et al., 2017; Mullican et al., 2017; Yang et al., 2017). Each of these soluble factors are covalent dimeric ligands and are members of the cystine knot/transforming growth factor β (TGF-β) superfamily (Hinck et al., 2016). Each GFL has a cognate GFRα (GFR) co-receptor that associate as GDNF-GFRα1 (Cacalano et al., 1998), NRTN-GFRα2 (Baloh et al., 1997), ARTN-GFRα3 (Baloh et al., 1998a), PSPN-GFRα4 (Thompson et al., 1998), and GDF15-GFRAL (Emmerson et al., 2017; Hsu et al., 2017; Mullican et al., 2017; Yang et al., 2017) complexes, respectively. The GFL co-receptors typically consist of three related helical domains (D1 to D3) and are anchored at the membrane either through glycosylphosphatidylinositol linkages (GFRα1-4) or by a transmembrane helix (GFRAL). The bipartite GFL-GFR complexes are recognized by the RET receptor tyrosine kinase (RTK) forming ternary RET-GFL-GFR complexes (Cacalano et al., 1998; Durbec et al., 1996; Jing et al., 1996; Treanor et al., 1996). Engagement of GFL-GFR by RET triggers RET auto-phosphorylation of critical tyrosine residues to activate intracellular signaling pathways (Ibáñez, 2013; Mulligan, 2014).

RET has four consecutive cadherin-like domains (CLD(1-4)) and a membrane-proximal cysteine-rich domain (CRD) in its extracellular domain (RET^ECD^) (Anders et al., 2001). The CLD domains diverge significantly, in sequence, structure, and arrangement from classical cadherins (calcium-dependent adhesion) (Anders et al., 2001; Brasch et al., 2012; Kjær et al., 2010). For example, the CLD(1-2) pair form a closed clamshell arrangement (Kjær et al., 2010). Calcium ions are critical for RET folding consistent with the conservation of calcium-coordinating motifs between CLD2 and CLD3 (Anders et al., 2001; Kjær and Ibáñez, 2003; van Weering et al., 1998). Biochemical efforts to map the bipartite GDNF-GFRα1 binding site within RET^ECD^ to a minimal-binding domain have implicated the entire RET^ECD^ region. This contrasts many receptor-ligand interaction RTKs that frequently map to an ~200 amino acid minimal-binding domain (Lemmon and Schlessinger, 2010). Two key interactions between RET^ECD^-GFRα1 and RET^ECD^-GDNF were identified from electron microscopy structures of RET^ECD^ bound to GDNF/NRTN and GFRα1/GFRα2, although lacking a CRD structure (Bigalke et al., 2019; Goodman et al., 2014). A study by Li et al. (2019) revealed a human RET^ECD^ structure, including the CRD, in complex with several GFL ligands. In this analysis, the D1 domain of GDNF-GFRα1 or GDF15-GFRAL complexes with RET^ECD^ were missing. Moreover, little information about conformational changes upon ligand binding was evident.

We report an X-ray structure of zebrafish RET^CLD−4^ and a cryo-electron microscopy (cryo-EM) structure of the zebrafish RET^ECD^-GDNF-GFRα1a complex. We observe plasticity within RET^CLD1-4^ and define the extent of conformational changes induced by ligand-co-receptor binding. Conformational adaptations are observed between RET and GFRα contacts even across clades, whereas a more strictly conserved interaction is observed between GFL and RET-CRD close to the transmembrane region. We show diversity in GFL co-receptor engagement by RET and describe RET^ECD^-GDNF-GFRα1a multimers on cryo-EM grids generating linear arrays.

## Results

### Crystal structure of zebrafish RET CLD(1-4) indicates localized flexibility

**Figure 1:**
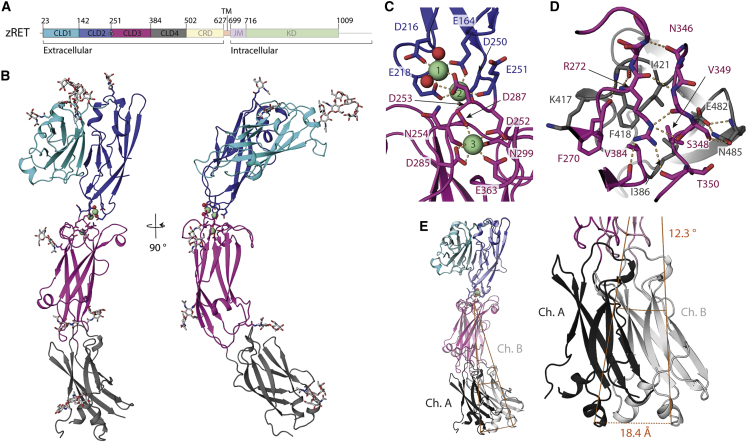
Crystal structure and flexibility of the zRET CLD(1-4) module (A) Schematic of zebrafish RET receptor tyrosine kinase. CLD, cadherin-like domains; CRD, cysteine-rich domain; TM, transmembrane helix; JM, juxtamembrane domain; KD, kinase domain. (B) Orthogonal views of zRET^CLD1-4^ colored as in (A). The calcium-binding site between CLD(2-3) has three calcium ions as green spheres with coordinating ligands as sticks and waters as red spheres. (C) Close-up view of the coordination shell for the three calcium atoms between CLD2 and CLD3. (D) Close-up of the interface between CLD3 and CLD4 centered on R272, selected side chains shown as sticks and dashed lines for hydrogen bonds. (E) Superposition of chains A and B within the crystallographic asymmetric unit.

Crystals were obtained for a zebrafish RET construct spanning residues 22–504 (zRET^22-504^) with glycosylation site mutations, N259Q, N308Q, N390Q, and N433Q (defined hereafter as zCLD(1-4)^red.sug.^). Diffraction data from these crystals led to a structure determination at 2.2 Å resolution (Figure 1; Table 1). The final zCLD(1-4)^red.sug.^ model contains residues 22–498 and includes 7 N-linked glycans well resolved in the electron density (Figure S1A). The crystals adopted the triclinic space group P1 and contained two molecules of CLD(1-4)^red.sug.^ within the asymmetric unit. Each had a similar overall structure but with different hinge angles between CLD2 and CLD3, pointing to flexibility within RET (Figure 1E).

**Table 1:** Crystallography data processing and refinement statistics

	zCLD(1-4)^red.sug.^	zGDNF^mat.^-GFRα1a^Δ^^D1^
Wavelength (Å)	0.9787	0.9795
Resolution range (Å) a	65.96–2.20 (2.28–2.20)	50.76–2.2 (2.28–2.2)
Space group	P 1	P 21 21 2

Unit cell dimensions		
a, b, c (Å)	51.17, 70.50, 105.44	125.07, 55.54, 70.96
α, β, γ (°)	105, 101, 100	90, 90, 90
Total no. of reflections	229,073 (22,789)	51,646 (5,054)
Unique reflections	67,550 (4,539)	25,823 (1,368)
Multiplicity	3.4 (3.4)	2.0 (2.0)
Completeness (%)	91.28 (66.36)	91.93 (53.94)
Mean I/σI	7.09 (1.92)	14.30 (3.15)
Wilson B factor (Å^2^)	28.88	20.87
R_merge_	0.073 (0.68)	0.056 (0.37)
R_meas_	0.087 (0.81)	0.079 (0.53)
R_pim_	0.046 (0.43)	0.056 (0.37)
CC½	0.996 (0.70)	0.997 (0.80)
CC	0.999 (0.91)	0.999 (0.94)
Resolution used for refinement	65.96–2.20	50.76–2.20
Reflections used in refinement	62,771 (4,522)	23,743 (1,363)
Reflections used for R_free_	3,098 (255)	1,152 (57)
R_work_	0.232 (0.316)	0.199 (0.247)
R_free_	0.277 (0.383)	0.230 (0.248)
CC (work)	0.895 (0.615)	0.888 (0.723)
CC (free)	0.884 (0.553)	0.888 (0.800)
No. of non-hydrogen atoms	7,997	2,736
Macromolecules	7,289	2,434
Ligands	539	83
Solvent	169	219
Protein residues	980	309

RMSD		
Bond lengths (Å)	0.009	0.006
Bond angles (^o^)	1.08	0.74

Ramachandran plot (%)		
Favored	96.6	97.03
Allowed	3.0	2.97
Outliers	0	0.0
Rotamer outliers (%)	0	0.0
Clashscore	14.56	4.03
Average B factor (Å^2^)	41.31	30.19
Macromolecules	39.53	28.99
Ligands	67.62	55.09
Solvent	33.82	34.06
No. of TLS groups	8	1
PDB:	7AMK	7AB8

RMSD, root-mean-square deviation.

a Values in parentheses are for highest-resolution shell.

The overall structure of zCLD(1-4)^red.sug^ showed that all CLDs have the predicted canonical seven β strand sandwich architecture of cadherin domains (Figure S1B) (Shapiro and Weis, 2009). The amino-terminal CLD1 is packed against CLD2 in a fold-over clamshell arrangement as anticipated from human RET, while CLD(2-4) forms a “C-shape” (Figure 1B). The zCLD(1-2) clamshell has a surprisingly large overall root-mean-square deviation (RMSD) of 18.9 Å over 229 Cαs when superposed with hCLD(1-2) (Winn et al., 2011). Key features contributing to this structural divergence are a shuffled disulfide connectivity, a lack of a β hairpin and a longer CLD1 helix α1 between higher and lower vertebrates (Figure S1C) (Kjær et al., 2010).

The irregular CLD2-β1 (residues 153–160) is largely separated from the main CLD2 sheet and lies between CLD1-β1 and CLD2-β7, anchored largely through CLD2-β2 side chains (such as R172 and R176) rather than main-chain interactions (Figure S1D). One end of CLD2-β1 is tethered through packing of two short α helices from CLD2-β1 and CLD2-β2, while the other end is anchored by aromatic side chains from residues amino-terminal to CLD1-β1. This configuration contributes to a substantial internal cavity between CLD1 and CLD2, with a surface volume of ~510 Å^3^ (Figure S1D). We note that analysis of the published human CLD(1-2) (Kjær et al., 2010) (PDB: 2X2U) also revealed a similar but smaller internal cavity of ~324 Å^3^ (Figure S1D) (Abagyan et al., 1994; An et al., 2005; Fernandez-Recio et al., 2005). On the opposing side of the clamshell interface, CLD1-β2 and CLD2-β2 contribute through both side- and main-chain interactions.

The limited size of the CLD(2-3) interface is typical of a calcium-dependent cadherin domain pair, with three calcium ions (Ca-1/Ca-2/Ca-3) and their coordinating ligands dominating the interface (Figure 1C) (Shapiro and Weis, 2009). Ca-1 and Ca-2 lie in close proximity (3.9 Å apart in chain A) and share three coordinating ligands, the side chains of E164, E218 (CLD2), and D253 (CLD3). Ca-1 is exposed to the solvent at the edge of CLD2, with the coordination sphere completed with D216 and two water molecules, one of which is coordinated by with N165 (Figure 1C). The Ca-2 coordination sphere includes D253, a main-chain carbonyl from E251 (CLD2), and D287 (CLD3), which is a ligand shared with Ca-3 (Figure 1C). Ca-3 is buried within CLD3 and located 6.9 Å away from Ca-2, the coordination shell is completed with the side chains of D252, D285, N299, and D363 and the main-chain carbonyl of N254 (Figure 1C).

CLD3 consists of 135 amino acids and is the largest RET CLD. It shows the greatest structural divergence of all CLDs (~5 Å RMSD) compared with the smaller canonical cadherin domains (Figure S1B) (Shapiro and Weis, 2009). Additional elements within CLD3 include a loop insertion between β2 and β3 adjacent to the calcium-binding site, an α helix between β3 and β4, and a much longer pair of antiparallel β strands, β4 and β5. Unusually, CLD3 lacks any disulfide bonds and its CLD4 interface is offset at one side of the domain giving a pronounced curvature to the entire CLD(1-4) module. CLD3 has five potential glycosylation sites, two of which were removed by site-directed mutagenesis in zCLD(1-4)^red.sug^ and three are visible in the electron density (Figure S1A). These features collectively ensure that CLD3 plays a crucial role in the stability and curvature of the zCLD(1-4) module.

The CLD(3-4) interface diverges substantially from classical cadherins and has previously confounded efforts to predict the precise CLD(3-4) domain boundaries (Anders et al., 2001). It lacks calcium ions and has a predominantly hydrophobic character, with peripheral hydrophilic interface contacts (Figure 1D). Hydrophobic contacts include CLD3 side chains F270 and V349 that pack against CLD4 F418 and I421 side chains and are tethered by V384 from a rigid connecting linker with sequence P383-V384-P385. An exception to the hydrophobic character of the interface is the buried R272 side chain from the CLD3-β1-β2 loop (Figure 1D). The aliphatic portion of R272 packs against V349, V384, and I421, while its guanidinium head engages main-chain carbonyls on the CLD3-β5-β6 loop and the CLD3-CLD4 linker (Figure 1D). This residue is equivalent to R287 in humans, a known site of mutation in a severe form of Hirschsprung's disease (R287Q), highlighting the crucial nature of this residue for folding (Attie et al., 1995; Pelet et al., 1998).

Differences in the CLD interface size indicate flexibility between CLD2 and CLD3 but rigidity between CLD3 and CLD4. This is supported by superpositions of the two independent molecules of zCLD(1-4)^red.sug^ demonstrating plasticity in the tapered CLD(2-3) interface (Figure 1E). Superimposing chain B onto chain A, aligned through CLD(1-2), reveals that the rigid CLD(3-4) module pivots about the CLD(2-3) calcium-binding site interface with a variation of 12.3°, which leads to a difference of 18.4 Å at the furthest point from the CLD(2-3) interface (Figure 1E). Subtle angular differences proximal to the calcium ions, propagating down the module lead to a tightening of the C-shaped structure between chain A and chain B.

### Cryo-EM structure of the ternary zebrafish GDNF-GFRα1a-RET^ECD^ complex

**Figure 2:**
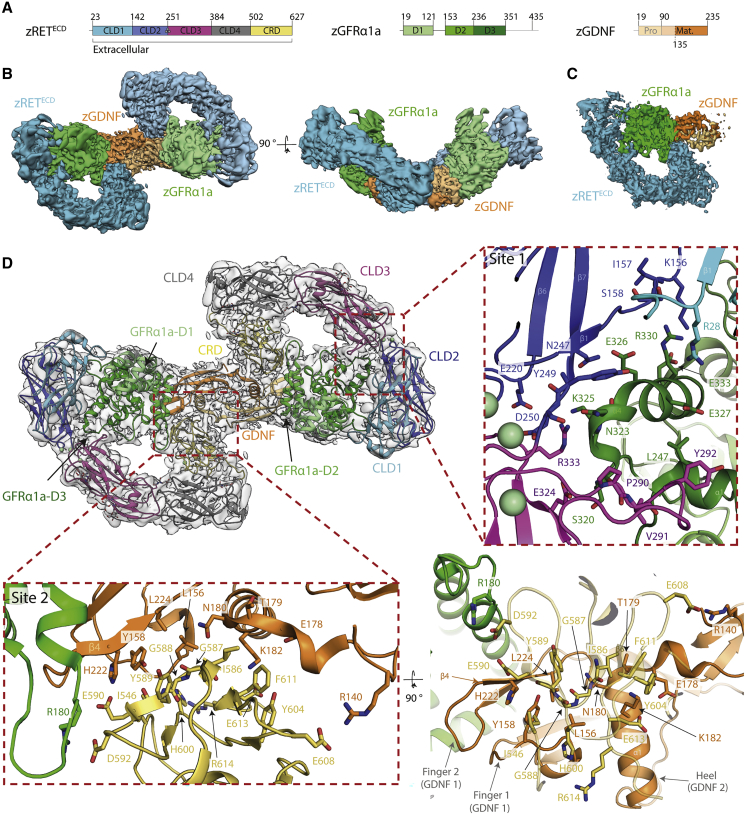
Cryo-EM structure of the zRET^ECD^-zGFRα1a^D1-3^-zGDNF^mat.^ (zRGα1a) complex (A) Schematic of zRET^ECD^, zGFRα1a^D1-3^, and zGDNF^mat.^, color coded according to Figure 1A. (B) Orthogonal views of the reconstituted zRGα1a complex cryo-EM map, projecting down the approximate molecular dyad or perpendicular to it. The cryo-EM map is segmented and colored by protein, with zRET^ECD^ cyan, zGFRα1a^D1-3^ green, and zGDNF^mat.^ orange. (C) Symmetry-expanded map of zRGα1a half-complex, with the map segmented and colored by protein as in (B). (D) The final model of the zRGα1a complex built into the C2 symmetry map, colored light gray. The domains are colored as in Figure 1 with zRET^ECD^: for GFRα1a domains D1-3 are pale green, green, and dark green, respectively; the two molecules of zGDNF^mat.^ are orange and pale orange. Two sites of interaction within the zRGα1a complex are highlighted in red dashed boxes, labeled as site 1 (zGFRα1a-zRET) and site 2 (zGDNF-zRET). Interaction residues are highlighted as sticks and the backbone represented as a cartoon.

A reconstituted complex was assembled consisting of the zRET^ECD^ (residues 1–627), a C-terminal truncated zGFRα1a (zGFRα1a^D1-3^) covering residues 1–353, and an N-terminal truncated zGDNF, residues 135–235 (zGDNF^mat.^), defined hereafter as zRGα1a from RET-GDNF-GFRα1a (Figures 2A and S2). The zRGα1a complex homogeneity and stability was improved by crosslinking using the GraFix technique (Kastner et al., 2008). An initial cryo-EM dataset (dataset 1) collected on the reconstituted zRGα1a yielded a 3D cryo-EM map that confirmed a 2:2:2 stoichiometry (see Figure S3), consistent with size-exclusion multi-angle laser light scattering data (Figure S2) and similar to recently published human RET complexes (Bigalke et al., 2019; Li et al., 2019). The map displayed substantial anisotropic resolution due to particle orientation bias on the grids. To overcome this, a second dataset was collected with a sample grid tilted at an angle of 30° (dataset 2) (see Figure S3). The combined particles from both datasets were used to generate an initial 3D volume with C2 symmetry applied in CryoSPARC-2 (Punjani et al., 2017). Additional processing with symmetry expansion in RELION-3 (Kimanius et al., 2016; Scheres, 2012; Zivanov et al., 2018), improved the anisotropy and resolution of the map by addressing flexibility at the 2-fold symmetry axis, to produce a map with a nominal resolution of 3.5 Å (Figures 2C, S4, S5A, and S5B). Subsequent analysis of this final map with 3DFSC indicated that there were a limited number of particles contributing to the Z direction of the 3D reconstruction, which resulted in the resolution in that direction being limited to ~10 Å (Figure S4) (Tan et al., 2017).

The zRGα1a cryo-EM map resembles a figure-of-eight with a molecular 2-fold centered at the crossover point (Figure 2B). To enable building of a full structure into the map, we determined a crystal structure of zGDNF^mat^-zGFRα1a^151-353^ lacking domain D1 (referred to hereafter as zGFRα1a^ΔD1^) at 2.2 Å (see the STAR Methods and Figure S5C). We then fitted crystal structures for zRET CLD(1-4) and zGDNF^mat^-zGFRα1a^ΔD1^ into the symmetry-expanded map (Figure S5C) together with homology models for the zRET^CRD^ and zGFRα1a^D1^. An initial model for zRET^CRD^ was generated from the hRET^ECD^-hGFRα2-NRTN structure (Li et al., 2019) and for zGFRα1a^D1^ from the hGFRα2-NRTN (Sandmark et al., 2018) structure by substituting zebrafish sequences followed by model optimization using Swiss-Model (Schwede et al., 2003) and Modeller (Webb and Sali, 2016), respectively. The initial structure was refined against the symmetry-expanded map and rebuilt, before placing it into the C2-averaged map for further refinement in PHENIX (Adams et al., 2010) (Table 2; Figures S5A and S5B). The final near complete structural model has a crosscorrelation of 0.63 against this map with highest-resolution features close to GDNF and zRET^CLD4−CRD^ (Figure S5). N-Acetylglucosamine (GlcNAcβ1-Asn) glycan rings linked to asparagine sites were also evident in the map. Density was also evident for zGFRα1a^D1^, sandwiched between zGFRα1a^D3^ and zRET^CLD1^, at a similar position to GFRα2^D1^ (Bigalke et al., 2019; Li et al., 2019; Sandmark et al., 2018) (Figure 2D).

**Table 2:** EM data acquisition and processing statistics

	zRGα1a C2 map	zRGα1a symmetry-expanded map	zR15AL negative stain map
EMDB:	EMD-11822	EMD-11822	EMD-11777
PDB:	7AML		
Magnification	46,296	46,296	40,719
Voltage (kV)	300	300	120
Electron exposure (e^−^/Å^2^)	48.6	48.6	–
Defocus range (μm)	1.4–3.5	1.4–3.5	1.0–1.5
Pixel size (Å)	1.08	1.08	3.44
Symmetry imposed	C2	C1	C2
Initial particle images	2,424,600 (dataset 1), 1,393,023 (dataset 2)	–	27,551
Final particle images	382,547 (360,189 dataset 1 and 22,358 dataset 2)	765,094	6,519
Map resolution (Å)	3.3	3.5	26
FSC threshold	0.143	0.143	0.143
Map resolution range (Å)	12–3.3	11–3.5	

**Refinement**	

Initial model, PDB:	7AMK, 7AB8
Model resolution (Å)	4.2
FSC threshold	0.5
Map sharpening B factor (Å^2^)	−75
Non-hydrogen atoms	16,020
Protein residues	1,996
Ligands	8
N-Glycans	16
Protein	122.4
Ligands	111.6
Bond lengths (Å)	0.004 (0)
Bond angles (°)	0.646 (6)

**Validation**	

MolProbity score	1.85
Clashscore	9.45
Poor rotamer (%)	0.89
Favored	94.94
Allowed	5.06
Disallowed	0.0

RMSD, root-mean-square deviation.

The final structure shows zGDNF at the core of the complex flanked by two zGFRα1a^D1-3^ co-receptors, both of which are further enveloped by two “G”-shaped RET^ECD^ molecules (Figure 2D). The spur of the RET^ECD^ G shape is formed by the CRD domain making contacts with both GDNF protomers and zGFRα1a, as first predicted from lower-resolution negative stain EM analysis (Goodman et al., 2014) as well as other structures (Bigalke et al., 2019; Li et al., 2019). There are two major interfaces between zRET^ECD^ and its ligand-co-receptor at opposite ends of zRET^ECD^, each is well defined in the cryo-EM map with side-chain level information (Figure 2D). The dominant interaction is between zCLD(1-3) and GFRα1^D3^ (defined hereafter as the site 1), with a key second site between zCRD and a concave surface presented by the GDNF dimer and a loop from GFRα1 (defined hereafter as site 2) (Figure 2D). Site 2 shows a close equivalence to the “low” affinity TGF-β receptor I binding site for TGF-β (Groppe et al., 2008; Kirsch et al., 2000) and is also used by other TGF-β superfamily ligands (Hinck et al., 2016).

Site 1 on zRET involves elements from the CLD(1-2) clamshell structure and the CLD(2-3) calcium-binding region (Figure 2D). Both contacts engage the zGFRα1 domain D3 (zGFRα1^D3^) close to helix α4, its preceding loop and helix α1 . Together these zGFRα1 elements form a wedge-shaped surface to access the calcium-binding region of zRET^CLD(2-3)^. This interface covers a total area of 846 Å^2^ and comprises both hydrophilic and electrostatic interactions, as calculated by PDBePISA (Krissinel and Henrick, 2007). The isolated CLD2-β1 strand bridges between the CLD1-CLD2 interface, running antiparallel to the zGFRα1^D3^ helix α4. Hydrophilic side chains from helix α4 interact with CLD2-β1 main chain as well as two proximal strands; CLD1-β1 and CLD1-β7 (Figure 2D). The side chain from R330 of zGFRα1^D3^ helix α4, lies close to the main-chain carbonyl of I157 from CLD2-β1 and the side chain of E326 is positioned near the side chains of N247 and Y249 (hydroxyl). The loop preceding helix α4 of zGFRα1a^D3^ is anchored between the CLD3-β2-β3 loop and the CLD3-β4-β5 loop; main chain-main chain interactions form between P290 from the CLD3-β2-β3 loop and S321 of zGFRα1a^D1-3^ (Figure 2D). The main chain of N323 from the loop preceding α4 of zGFRα1a^D1-3^ appears to interact with the guanidinium head of R333 from CLD3-β4, and the side chain of N323 interacts with the main chain of D250 at the calcium-binding site (Figure 2D).

Site 2 interaction involves the zRET^CRD^ and a concave “saddle”-shaped surface formed by both protomers of the zGDNF^mat.^ dimer and a loop from zGFRα1a^D2^ (Figure 2D). This agrees with our previous assignment of this site as a “shared” site (Goodman et al., 2014). The interface is mainly hydrophobic in character and has a surface area of 598 Å^2^. The surface contains three main elements; a β-turn from zGFRα1^D2^ centered on R180, residues 156–159 (LGYR) and residues 222–224 (HTL) from the fingers of one GDNF protomer (GDNF1) and residues 176–179 (DATN) with the “heel” helix of the second protomer (GDNF2). These residues engage G588 and Y589 from the CRD-β3-β4 loop (Figure 2D) and make van der Waals's contacts to the I546 side chain from the CRD-β1-β2 loop (Figure 2D). A hydrophobic interaction is evident between I586 from the CRD-β3-β4 loop and the T179 from the loop preceding the zGDNF2 heel (Figure 2D). The remaining contacts are mainly hydrophilic in nature between the heel of GDNF2 and the CRD. From the heel of zGDNF2; N180^GDNF^ interfaces with the amide of G587, and K182 of GDNF2 interacts with E613. This contact is consistent with the absence of a crosslink in the XL-MS data (Figure S6). The zRET^CRD^ β5-β6 β turn is 2 amino acids shorter than hRET^CRD^ allowing it to engage amino-terminal residues 138–140 of zGDNF2 with a likely salt bridge between E607 and R140. Also, H222 from zGDNF1 is likely to contact E590 (equivalent to E595 in human RET, a known Hirschprung's mutation site) (So et al., 2011).

Two further contacts with zRET are indicated but are less well defined in the map. A limited interface between zRET^CLD1^ and GFRα1^D1^ is observed allowing zRET^CLD1^ and GFRα1^D1^ domains to be placed and the interaction is very similar to that seen in the RET^ECD^-NRTN-GFRα2 structure (Li et al., 2019). Second, residues immediately after the CRD from residues 615 to 627 are poorly ordered. This acidic stretch includes 12 residues likely to pass beneath the highly basic GDNF ligand (pI of 9.3 for mature zGDNF) before entering the plasma membrane. The final residue in RET^ECD^ observed is P617, which is separated by a distance of 40.9 Å from the dimer equivalent residue. A lower map contour shows density for these residues beneath the GDNF molecular 2-fold axis consistent with RET^ECD^-NRTN-GFRα2 (Bigalke et al., 2019).

### Clade-specific features influence ligand binding affinity

**Figure 3:**
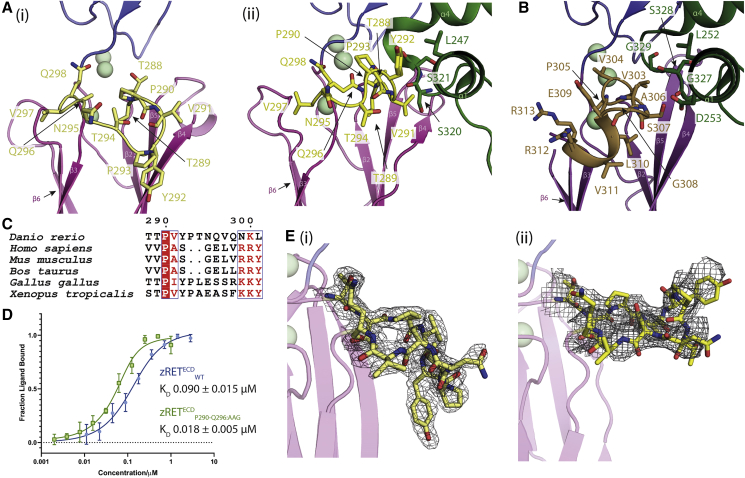
Ligand-co-receptor-induced conformational changes in zRET^ECD^ (A) The CLD3-β2-β3 loop is shown in yellow as sticks (i) projects “downward” in the view shown for zRET CLD(1-4) (see the orientation of Y292 side chain) and (ii) projects “upward” to engage the GFRα1^D2^ α1 helix (green sticks) in the zRGα1a structure. (B) The shorter CLD3-β2-β3 loop and extra helix from the human RET^ECD^-NRTN-GFRα2 structure (PDB: 6Q2O) shown as olive colored sticks, domains colored as in Figure 1. (C) Sequence alignment of RET CLD3-β2-β3 loop sequences by Espript (http://espript.ibcp.fr) (Robert and Gouet, 2014). (D) Binding curves and calculated K_D_ values for zRET^ECD^_wt_ and mutant (zRET^ECD^_P291-Q296:AAG_) binding to zGFRα1a_2_-zGDNF_2_ measured by MST, with a minimum of n = 3 repeats for the WT and the mutations with the SE represented. (E) (i) Electron density map calculated using m2Fo-DFc coefficients over the CLD3-β2-β3 loop, yellow sticks and contoured at 1.0σ. (ii) Coulombic potential cryo-EM map for CLD3-β2-β3 loop from the zRGα1a complex (black mesh). Calcium ions are represented as pale green spheres.

Comparison of site 1 of zRET in both the crystal and cryo-EM structure reveals differences in the conformation of residues 288–298 from a CLD3 loop (Figure 3A). In the absence of ligand, this loop packs against CLD3 core (loop “down” position) interacting with the β4 strand. In the presence of the ligand, this loop forms a central part of the interface with zGFRα1a^D3^ and is repositioned upward (loop-“up”) toward the calcium ions and engages L247 of helix α1 of zGFRα1a^D2^ (Figure 3A). No equivalent interaction is observed for the human RET CLD3 structure (Figure 3B). The cryo-EM map clearly shows zGFRα1a^D3^ side-chain contacts with Y292 and how this residue shifts substantially relative its unliganded position (Figure 3C). This movement of 18.5 Å (hydroxyl-hydroxyl) or 8.3 Å (Cα-Cα) also results in main chain amides from P290 and V291 of the CLD3-β2-β3 loop lying close to the main-chain carbonyl of S320 from zGFRα1a^D3^, forming a pseudo-β sheet interaction (Figure 3A).

In view of the critical role of this loop in the zRET co-receptor recognition, it is surprising that loop CLD3-β2-β3 contains an “indel” of two extra amino acids Y292 and P293, unique to lower vertebrates (Figure 3D). The equivalent shorter loop in human RET adopts a helical turn connecting the two β strands (Figure 3C) (Li et al., 2019). To probe the contribution of the CLD3-β2-β3 loop to zGDNF-zGFRα1a binding, we truncated the residues P290-Q296 to AAG and assessed its ligand binding properties by microscale thermophoresis (MST). Surprisingly loop truncation improved binding affinity for the ligand-co-receptor by 5-fold compared with wild-type (WT) zRET^ECD^, with a dissociation constant (K_D_) of 18 nM (±5 nM) compared with 90 nM (±15 nM) for WT zRET (Figure 3E). This increase in affinity implies either that higher vertebrates RET^ECD^ have a higher affinity for ligand than their lower vertebrate counterparts or that the loop contributes to an auto-inhibitory function in lower vertebrates. Taken together, our structural results show an unexpected conformational change in a clade-specific loop proximal to the CLD(2-3) calcium sites.

Comparisons of interfaces within ternary RET complexes either between species (human and zebrafish GDNF-GFRα1) or paralogs (Neurturin-GFRα2 and GDF15-GFRAL) reveal considerable variation in contacts at site 1 and nearly identical contacts at site 2. This translates into a substantial variation in the size of these interfaces (Table S1).

One contributing factor to these variations is the additional contacts seen between helix α1 of zGFRα1^D^^3^ and residues 288–298 of zRET. Another example is GFRAL, which makes multiple additional contacts through residues 247–266, centered on the disulfide C252-C258. These contacts engage residues flanking the β hairpin at Y76/R77 and R144/Y146 on the CLD1 β7 strand. Both elements are unique to higher-vertebrate RET and contribute to the ligand-free RET dimer interface (Kjær et al., 2010; Li et al., 2019).

**Figure 4:**
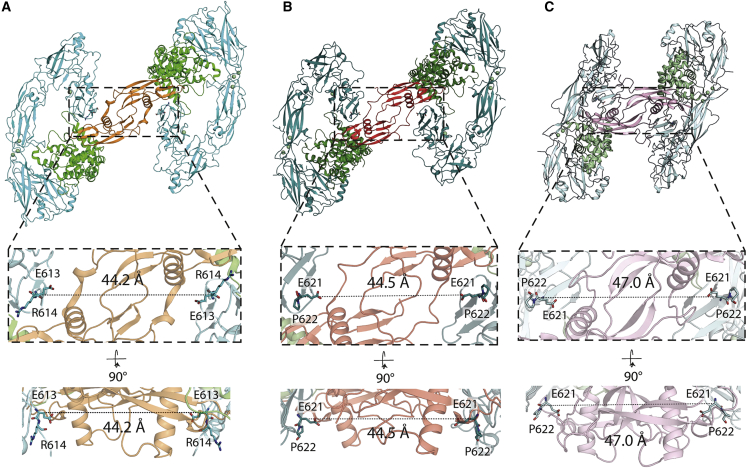
Different GFL ligands establish a conserved spacing between RET CRD-CRD pairs in their respective ternary complexes (A) Separation between the Cα of E613 (equivalent to E620 of hRET) from both molecules of zRET^ECD^ within the zRGα1a structure. (B) Equivalent distance between the Cα E620 from both molecules of hRET^ECD^ from the hRET^ECD^-NRTN-GFRα2 (PDB: 6Q2O) structure. (C) Equivalent separation between the Cα E620 from the two molecules of hRET^ECD^ from the hRET^ECD^-GDF15-GFRAL (PDB: 6Q2J) structure. The overall structure is represented as a cartoon and the Ca^2+^ ions are represented as spheres. RET is colored cyan, teal, and pale cyan in zRGα1a, hRET^ECD^-NRTN-GFRα2, and hRET^ECD^-GDF15-GFRAL structures, respectively. GFRα1a, GFRα2, and GFRAL are colored green, dark green, and pale green, respectively. GDNF, NRTN, and GDF15 are colored orange, red, and light pink, respectively.

Comparison of all available liganded RET^ECD^ structures at site 2 consistently show a spacing of 44.2–47.0 Å between each pair of CRD C termini (measured at residue E613/620 in zRET/hRET) within an RET dimer (Figures 4A–4C). This suggests a stringent requirement for CRD spacing to couple the transmembrane and intracellular modules. We note this distance is defined by the geometric length of a GFL ligand dimer and the position of the CRD relative to the dyad-axis of GDNF, presumed to sit above the RET transmembrane region.

### Structure-function analysis of zRET-GDNF-GFRα1a interaction sites

**Figure 5:**
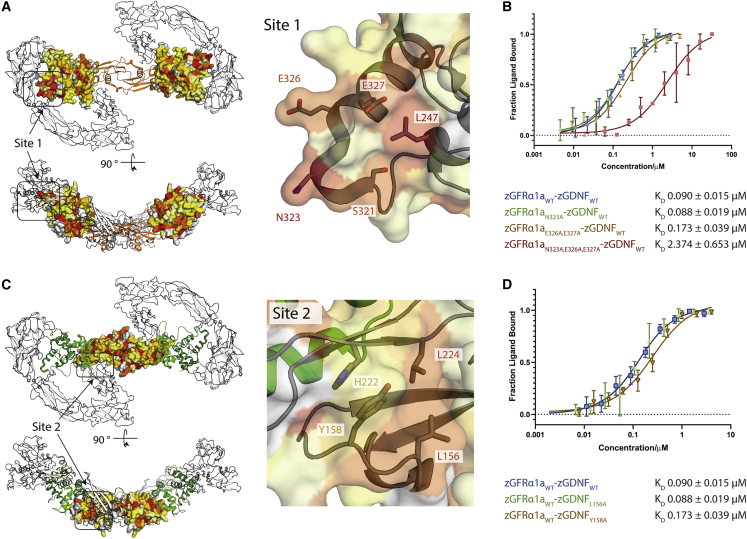
Mutational analysis of zGDNF and zGFRα1 site 1 and 2 interactions with zRET^ECD^ (A) Heatmap of the sequence conservation between hGFRα paralogs, and zGFRα1a mapped onto the structure of zGFRα1a D2-D3 domains reported here. Residues are colored by similarity (red highly similar to yellow through to white, least similar). Two orthogonal views are shown. Right panel, close-up of site 1 and conserved zGFRα1a residues. (B) Binding curves and K_D_ values obtained using MST for zGFRα1a^D1-3^ and mutations assessed in complex with zGDNF^mat.^, with a minimum of n = 3 repeats for the WT and the mutations with the SE represented. (C) Heatmap of the sequence similarity between GDNF paralogs depicted as a surface representation, mapped onto zGDNF^138-235^. Right panel, close-up of site 2 contact between RET^CRD^ and zGDNF dimer. (D) MST binding curves and K_D_ values for zGDNF and mutations L156A and Y158A probed in complex with WT zGFRα1a binding to zRET^ECD^.

The importance of each zRET interaction site on ligand-complex assembly was probed by mutation of the GDNF co-receptor at site 1 or GDNF at site 2. Surface residue heatmaps identified the loop-helix α4 element of zGFRα1a^D3^ contributes residues N323, E326, and E327 to the RET-co-receptor interface and are present in most GFRα sequences (Figure 5A). These residues were mutated to alanine, both individually and as a triple mutant. Using solution-based MST, affinity measurements of zGDNF^mat.^_WT_-zGFR1α1a^D1−3^_N323A_ and zGDNF^mat.^_WT_-zGFR1α1a^D1−3^_E326A,E327A_ complexes binding to fluorescently labeled zRET^ECD^ indicated only a modest impact, with a 2-fold decrease in affinity of E326A-E327A, corresponding to a K_D_ of 0.17 ± 0.039 μM versus 0.090 ± 0.015 μM for zGDNF ^mat.^_WT_-zGFR1α1a^D1−3^_WT_ (Figure 5B). However, when combined as a triple mutation, zGDNF_wt_-zGFR1α1a^D1−3^_N323A,E326A,E327A_, a 25-fold reduction in affinity was observed (K_D_ of 2.35 ± 0.653 μM) (Figure 5B).

To probe the contribution of site 2 interface residues (Figure 5C) L156, Y158, L224, and E220/H222 of zGDNF^mat.^ were selected for mutation to alanine and prepared using insect cells co-expressed with WT zGFRα1a^D1-3^. The L224A and E220A/H222A mutations adversely affected the expression of zGDNF^mat.^ and could not be evaluated. MST was used to test the affinity of zGDNF^mat.^_L156A_-zGFRα1a^D1−3^_WT_ and zGDNF^mat.^_Y158A_-zGFRα1a^D1−3^_WT_ toward zRET^ECD^. A 2-fold decrease in affinity observed for zGDNF^mat.^_Y158A_ toward zRET^ECD^, whereas no substantial loss in affinity was observed for zGDNF^mat.^_L156A_ (Figure 5D). We interpret the minimal effect of these mutations to zGDNF^mat.^ on zRET^ECD^ binding is indicative of a low-affinity interaction site relative to the zCLD(1-3)-zGFRα1a^D3^ site 1. Taken together, the data for zRET loop deletion and targeted zGFR1α and zGDNF mutations point to site 1 being the dominant high-affinity binding site despite both sites being required for ternary complex assembly.

### Different D1 domain orientation between GDNF and GDF15 co-receptor complexes

**Figure 6:**
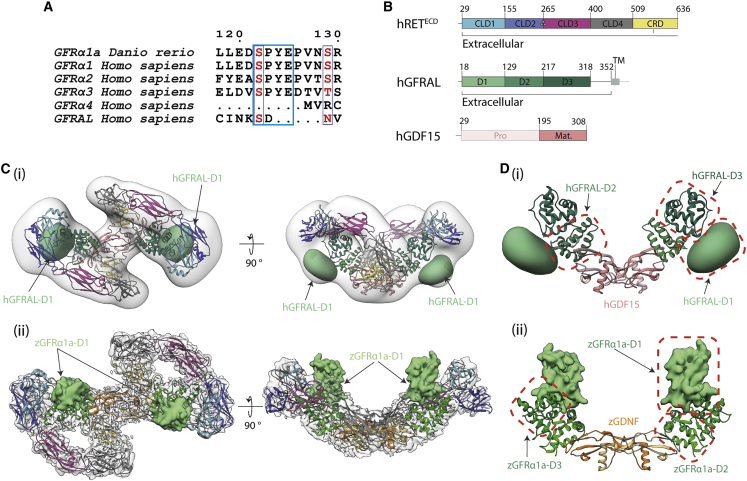
Divergent GFRα1/GFRAL co-receptor D1 domain positions within the RET^ECD^ ternary complex (A) The D1-D2 domain linker motif (SPYE), highlighted in cyan is conserved between zGFRα1a, GFRα1, GFRα2, and GFRα3. It is missing from the shorter GFRα4 that lacks a D1 domain altogether and from the divergent GFRAL. (B) Schematic diagram of human RET^ECD^, GFRAL, and GDF15 construct boundaries used and individual domains annotated as in Figure 1. (C) (i) Negative stain EM envelope of a reconstituted hRET^ECD^_2-_hGDF15_2_-hGFRAL_2_ (hR15AL) complex docked with hR15AL (PDB: 6Q2J) revealing additional map potential indicated by a green Gaussian volume (generated from a D1 domain homology model). (ii) Cryo-EM map of zRGα1a (light gray) superposed with the final model (colored as in Figure 2) with GFRα1a^D1^ shown (light green Gaussian volume at 5 Å^2^). (D) Comparison of co-receptor D1 domain position and interfaces (i) GFRAL^D1^ makes different contacts to domains D2-D3 (green), GFRAL^D1^ shown as a 30 Å^2^ Gaussian volume (light green), and GDF15 (salmon). (ii) zGFRα1a^D1^ contacts and colored as in Figure 2. zGFRα1a^D1^ represented as a 5 Å^2^ Gaussian volume (light green).

In the zRGα1a cryo-EM structure, the GFRα1^D1^ domain packs against GFRα1^D3^ using a linker with a conserved SPYE motif that is retained in all co-receptor sequences except GFRα4 and GFRAL (Figure 6A). We therefore hypothesized that GFRAL^D1^ may require different contacts with RET through a distinctive D1-D2 linker sequence. To explore this possibility, a ternary complex was assembled comprising the hRET^ECD^, hGDF15^mat.^ (hGDF15^195−380^), and hGFRAL^D1-3^ (hGFRAL^18-318^) (referred to hereafter as hR15AL) (Figure 6B) and crosslinked using GraFix (Figure S7). A low-resolution negative stain envelope was produced with a total of 6,519 particles with C2 symmetry averaging applied (Figures 6C and S7). While the overall shape of the envelope is similar to that of the zRGα1a map with a winged figure-of-eight appearance, it was evident that the wings are at a more acute angle to one another than in the zRGα1a cryo-EM map corresponding to a more “upright” hR15AL complex than the zRGα1a complex (Figure 6C).

Docking the recently published hRET^ECD^GDF15^mat.^GFRAL^129-318^ cryo-EM structure (PDB: 6Q2J) (Li et al., 2019) into the low-resolution envelope corroborated the more upright position of both hRET copies. It also revealed substantial density not accounted for by the fitted model, located beneath CLD(1-2) and flanking domains 2 and 3 (D2 and D3) of GFRAL (Figure 6C). The lack of domain 1 (D1) in the fitted model indicates that the area of unoccupied density is most likely the location of GFRAL^D1^ (Figure 6C). Such a position is in marked contrast to that of zGFRα1a^D1^ in zRGα1a (Figure 6D). This indicates a substantial plasticity in GFRAL as the most divergent GFR family member, explaining its lack of sequence conservation within the D1-D2 linker. It also emphasizes further the ability of RET^ECD^ to accommodate a variety of ligand-co-receptor geometries from the flatter ARTN-GFRα3 to the upright GDF15-GFRAL complex, as shown by Li et al. (2019). Some flexibility was apparent within the zGFRα1a^Δ1^-zGDNF^mat.^ co-receptor-ligand complex itself and had been noted previously (Parkash and Goldman, 2009). The 2:2 complex X-ray crystal structure in the absence of RET^ECD^ has a distance of 127.9 Å between K325 of each GFRα1a protomer whereas in the presence of RET^ECD^ this distance increases to 131.3 Å (Figure S8). Further studies are required to map in detail the additional interactions provided by GFRAL^D1^ to bind RET. We conclude that plasticity is not only evident within RET^ECD^ in accepting different GFL ligand-co-receptor geometries but also points to different roles for domain D1 between paralogs.

### Linear arrays of RET^ECD^-GDNF-GFRα1a observed on cryo-EM grids

**Figure 7:**
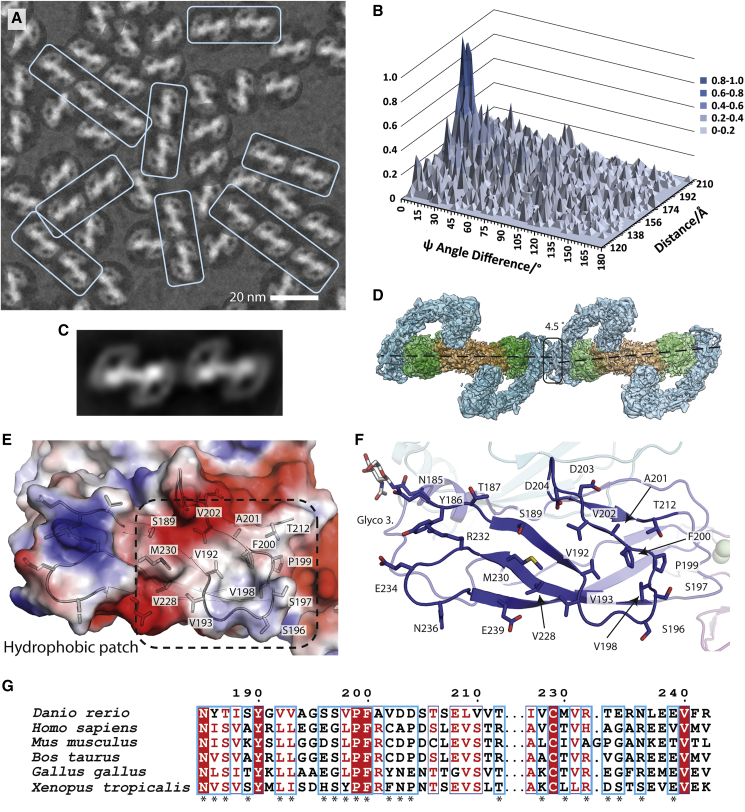
Evidence for linear arrays of zRGα1a particles on cryo-EM grids (A) Close-up of a representative micrograph for zRGα1a showing the particle orientation bias by fitting the dominant 2D class average view into picked particles and a recurring linear array of particles highlighted within pale cyan boxes. (B) Statistical distribution of the difference between the angle psi (Δψ) between two particles and their separation distance. Here the angle ψ is defined for each particle as the angle of rotation of each particle required to align it onto the 2D class average. (C) 2D class average from automated particle picking containing two adjacent zRGα1a particles. (D) The zRGα1a-zRGα1a interface highlighted with a black box. The angle and separation between each complex is based on the peak maxima coordinates from (B) assuming both particles are at the same Z height. (E) An electrostatic potential surface with selected side chains for the homotypic zRET^CLD2^ interface. (F) Close-up of the CLD2 contact, highlighting interface residues. (G) Conservation of representative RET sequences at the CLD2-CLD2 interface shown with an asterix.

Cryo-EM micrographs of the non-crosslinked sample of zRGα1a revealed significant orientation bias of the zRGα1a particles and a single predominant view projecting down the zRGα1a molecular by 2-fold (Figure 7A). Upon closer inspection using RELION particle reposition (Zivanov et al., 2018) a homotypic interaction between zRGα1a particles was observed throughout the grids resulting in linear arrays of complexes (Figure 7A). These arrays can consist of between two and four particles in length. We analyzed 3,756 randomly picked particles from 14 micrographs. Using an interparticle distance of 214.2 Å (170 pixels) from the centroid of one particle to the centroid of neighboring particles (x, y coordinates from the star file), 4,132 particle pairs were defined. A 3D surface distribution plot of the difference in psi angles (Δψ) for pairs of particles against the distance between their centroids was calculated (Figure 7B), the ψ angles are generated in RELION 2D classification (Kimanius et al., 2016; Zivanov et al., 2018). An error of 3° exists within the plot due to the angular sampling value used during 2D classification. The 3D plot revealed that particles at a distance of 181 ± 3 Å from one another have an average Δψ of 4.5° ± 2.3°, using a minimal frequency of Δψ to average distance of the more than 0.5 (Figure 7B). The recurrent and repetitive nature of this end-to-end contact for neighboring particle pairs was further captured in a 2D class average, which used 1,194 particle pairs (2,388 individual particles) (Figure 7C).

Using the information gathered from the particle pair analysis, two copies of the zRGα1a complex structure were aligned with an interparticle distance ~180 Å apart and an angle of 4.5° between the two copies (Figure 7D). Observations of both the single particle as well as the 2D class averages generated for a pair of zRGα1a complexes show that the two wings of the figure-of-eight structure do not appear to be symmetrical, with a slightly more acute angle appearing between zGFRα1a and zGDNF on the sides in contact with one another in the neighboring particles (Figures 7A and 7C). The interparticle interaction site observed on cryo-EM grids maps to a predominantly hydrophobic surface of CLD2, centered on V192 and V202 (Figures 7E and 7F). This hydrophobic patch is conserved between lower and higher vertebrates and is flanked by both basic (R232) and acidic clusters (D203, D204, and E239) that reciprocally neutralize each other across the zRGα1a-zRGα1a interface (Figure 7G). We note a highly conserved glycosylation site at N185 of CLD2 (found in both higher and lower vertebrates) is situated on the periphery of the multimerization interface (Figure 7G, glyco-site 3). In a linear array context, this glycan could potentially interact with calcium ion Ca-1 (near the CLD(2-3) junction) of an adjacent ternary complex to complete its coordination shell in *trans*. Further analyses are required to demonstrate a functional role for this multimeric interaction for full-length RET in a cellular context. Nevertheless, the high-sequence conservation within the interface points to an important role beyond ligand-co-receptor interaction.

## Discussion

Here, we establish principles for understanding the assembly of RET ligand-co-receptor complexes. We rationalize how RET can accept a range of activating GFL-co-receptor binary complexes through conformational adaptations between RET and co-receptor. By using crystallography and cryo-EM we define the architecture and ligand recognition properties of zebrafish RET^ECD^ and compare this with the human RET^ECD^. Our results provide four main insights; (1) there is conformational flexibility at the CLD(2-3) interface of RET^ECD^ that contributes to optimized adaptations at the co-receptor binding site; (2) there are conformational differences between unliganded and liganded RET centered on a clade-specific RET loop; (3) a strict spatial separation of RET^ECD^ C termini within the ternary complex is imposed by each CRD interaction with a GFL dimer; (4) differences in co-receptor engagement and putative higher-order multimers of ligand-bound RET suggest divergent interactions at each level of receptor engagement.

Previous insights into GFL-co-receptor recognition from negative stain and cryo-EM have revealed two main contact sites in RET (Bigalke et al., 2019; Goodman et al., 2014; Li et al., 2019). These structures explained why an intact calcium-loaded RET^ECD^ is required for GDNF-GFRα1 binding as the GFRα1^D3^ loop-helix α4/GFRα1^D2^ helix α1 wedge targets the calcium-dependent CLD(2-3) hinge while the GDNF dimer targets the CRD. The GFRα1 wedge may act as a sensor for calcium bound to RET implicating calcium not only in promoting RET folding but also proper recognition by co-receptor for signaling (Nozaki et al., 1998). The RET^CRD^ interaction with both protomers of a GDNF dimer is directly equivalent to the binding site of “low-affinity” TGF-β/BMP family of type 1 receptors for TGF-β (“knuckles” and “thumb”) (Hinck et al., 2016). Whereas the TGF-β “fingers” engage the high-affinity TGF-β receptor, equivalent to GFRα co-receptors binding to GFL fingers.

Several studies identified a role for site 1 contacts close to N323 in RET ternary complex formation (Goodman et al., 2014; Bigalke et al., 2019; Li et al., 2019). The strikingly distinctive contacts made by different GFRα homologs at site 1 suggest conformational adaptations enable the recognition of multiple GFRα co-receptors and different GFR_2_GFL_2_ geometries. Our findings suggest that engagement of ligand-co-receptor through the calcium-dependent CLD(2-3) hinge promotes a remodeling of the lower-vertebrate-specific loop and may precede site 2 RET^CRD^ engagement. This could involve either a pre-assembled RET-GFRα complex or presentation of GFRα after dimerization by GFL, before RET^CRD^ interaction. We show here from substitution of zGDNF residues in site 2 (L156A and Y158A) that these contacts do not appear to play a dominant role in ternary complex assembly. This contrasts with a study showing mutation of Y119 to E in Neurturin (equivalent to Y158 of zGDNF) disrupted ternary complex formation and signaling (Bigalke et al., 2019). Given the analogous RET^CRD^ contacts at site 2 for each GFL dimer are proximal to the RET transmembrane segment, this suggests an organizing role for signal transduction in addition to contributing to ligand recognition.

The D1 domain is missing from previous structures of GDNF-GFRα1 and GDF15-GFRAL, but had been observed for NRTN-GFRα2 alone or bound to hRET^ECD^ (Bigalke et al., 2019; Li et al., 2019). We were able to place the GFRα1 domain D1 adjacent to zRET^CLD1^, consistent with previous negative stain EM models (Goodman et al., 2014). As previously shown, the D1 proximity to RET^CLD1^ is not essential for ternary complex formation. We present evidence for a quite different contact position for the GFRAL D1 domain adjacent to GFRAL D2 and D3 domains, on the outside of RET and underneath its “wings.” This explains the absence of the otherwise conserved SPYE motif common to GFRα1/2/3 motifs at the D1 and D3 interface. This position for the GFRAL D1 domain arises from a more upright position for GFRAL observed than GDNF-GFRα1 complexes (Li et al., 2019). While the functional significance of this difference is yet to be understood, it could impact on ligand-biased signaling outputs or the assembly of higher-order multimers, such as those observed for zRGα1a.

We and others have provided structural evidence for RET dimers in the absence of a ligand-co-receptor through a CLD1-2 dimer interface involving R77 and R144 side chains (Kjær et al., 2010; Li et al., 2019). Here, we describe a ligand-dependent linear array of zGDNF-zGFRα1a-zRET^ECD^ complexes observed throughout the cryo-EM micrographs. This dominant mode of multimerization observed on micrographs is mediated by a homotypic hydrophobic patch on an exposed part of CLD2 (CLD2-CLD2 interface). The arrangement is distinct from but compatible with the “stacked” interaction observed between two hNRTN-hGFRα2-hRET^ECD^ ternary complexes by Li et al., 2019. The stacked interaction involved contacts between hRET^CLD4^ and NRTN and was reported to influence the rate of receptor endocytosis. We do observe occasional stacked particles packed in this manner but we cannot conclude their significance at this point and the zRET^CLD4^ stacked interface is not conserved. These findings suggest that a signaling-competent RET^ECD^ conformation is likely to involve higher-order multimers consistent with findings for other RTKs, such as EphR (Seiradake et al., 2010), EGFR (Needham et al., 2016), and DDR1 (Corcoran et al., 2019) RTKs. Therefore, a crucial aspect of receptor activation beyond the positioning of the RET transmembrane regions within a dimeric assembly may prove to be their arrangement within higher-order clusters.

In summary, this study reveals several under-appreciated aspects of GFL-co-receptor binding to RET, including receptor flexibility, clade-specific adaptations, and conformational changes. All these features reveal a substantial tolerance within RET to accommodate different GFL-co-receptors using a flexible arm. It also suggests that a key requirement for coupling ligand binding to RET activation is a strict spatial separation between CRD C termini within RET dimers imposed by the geometric dimensions of each GDNF family ligand. The next challenge will be to visualize such arrangements of a full-length RET multimer in a membrane context and to use this knowledge in the design of both antagonist and agonist biologicals that with therapeutic utility.

## STAR★Methods

### Key resources table

**Table:** 

REAGENT or RESOURCE	SOURCE	IDENTIFIER
**Chemicals, peptides, and recombinant proteins**		

Gibco ™ Sf-900-III SFM	Thermo Fisher	Cat# 12658019
Lactalbumin	Sigma Aldrich	Cat# 58901C-100ML
Yeastolate	Sigma Aldrich	Cat# 58902C
Ex-Cell420 media	Sigma Aldrich	Cat# 14420C-1000ML
Fetal Bovine Serum	Thermo Fisher	Cat# 10082147
ExpreS^2^-Insect TR	Expression Systems	Cat# 95-055-075
Gibco ™ FreeStyle™ 293 Expression Medium	Thermo Fisher	Cat# 12338026
Polyethylimine	Polysciences	Cat# 19850
Optimem	Thermo Fisher	Cat# 31985062
FuGENE® HD Transfection Reagent	Promega	Cat# E2311
FlashBAC Gold Baculovirus Expression System	2B Scientific	Cat# GWB-67B0AE
Glutaraldehyde (50 % v/v)	Sigma Aldrich	Cat# 49628
Recombinant protein: zebrafish RET (aa 1-504, ref# A8E7C6)	This paper	N/A
Recombinant protein: zebrafish RET (aa 1-626, ref# A8E7C6)	This paper	N/A
Recombinant protein: zebrafish GFRα1a (aa 1-353, ref# Q98TT9)	This paper	N/A
Recombinant protein: zebrafish GDNF (aa 135-235, ref# Q98TU0)	This paper	N/A
Recombinant protein: human RET (aa 1-635, ref# P07949)	This paper	N/A
Recombinant protein: human GFRAL (aa ref# Q6UXV0)	This paper	N/A
Recombinant protein: human GDF15 (aa ref# Q99988)	This paper	N/A

**Critical commercial assays**		

Monolith Protein Labeling RED-NHS 2^nd^ generation (Amine Reactive)	Nanotemper	Cat# MO-L001
Nanotemper hydrophobic capillaries	Nanotemper	Cat# MO-KO23

**Deposited data**		

zRET^CLD1-4^ crystal structure	This paper	PDB: 7AMK
zGFRα1^150-353^-zGDNF^135-235^ complex crystal structure	This paper	PDB: 7AB8
zRET^22-595^- zGFRα1^29-353^-zGDNF^135-235^ complex cryo-EM structure	This paper	PDB: 7AML
The zRGα1a C2 symmetry / the zRGα1a symmetry expanded cryo-EM maps	This paper	EMD11822
The hR15AL negative stain EM map	This paper	EMD11777
hRET^CLD1-2^ crystal structure	Kjær et al., 2010	PDB: 2X2U
hRET^ECD^-GDF15^mat.^GFRAL^129-318^ cryo-EM structure	Li et al., 2019	PDB: 6Q2J
hGDNF-hGFRα1^D2-D3^ crystal structure	Parkash and Goldman (2009)	PDB: 3FUB
hGFRα2-neurturin crystal structure	Sandmark et al., 2018	PDB: 5MR4
hRET^ECD^-GFRα2-neurturin cryo-EM structure	Li et al., 2019	PDB: 6Q2O
C-cadherin ectodomain crystal structure	Boggon et al., 2002	PDB: 1L3W
N-cadherin EC1 domain crystal structure	Shapiro et al., 1995	PDB: 1NCI
N-cadherin EC1 domain solution structure	Koch et al., 2004	PDB: 1OP4
Protocadherin Beta 1 EC1-3 crystal structure	Rubinstein et al., 2015	PDB: 4ZPL
Protocadherin Alpha C2 EC1-3 crystal structure	Rubinstein et al., 2015	PDB: 4ZPM
Protocadherin Gamma C5 EC1-3 crystal structure	Rubinstein et al., 2015	PDB: 4ZPO
Protocadherin Gamma A8 EC1-3 crystal structure	Rubinstein et al., 2015	PDB: 4ZPS
E-cadherin domains 1 and 2	Nagar et al., 1996	PDB: 1EDH

**Experimental models: cell lines**		

Human: Expi293 cells	Thermo Fisher	Cat# A14527
Insect: Sf21 cells	Thermo Fisher	Cat# 11497013
Insect: Hi Five cells	Thermo Fisher	Cat# B85502
Insect: *Drosophila* S2 cells	Thermo Fisher	Cat# R69007

**Recombinant DNA**		

Plasmid: pCEP	Kohfeldt et al., 1997	N/A
Plasmid: pCEP-hGFRAL-Histag	This paper	N/A
Plasmid: pCEP-hGDF15-Histag	This paper	N/A
Plasmid: pExpreS2.1	Expres2ion Biotech	Cat# S2-11A-001
Plasmid: pExpreS2.1-hRET^1-635^-Tev-Avi-Ctag	This paper	N/A
Plasmid: pBacPak	Clontech Laboratories, Inc.	Cat# 631410
Plasmid: pBacPak-Mellitin1-20-zGDNF^135-235^	This paper	N/A
Plasmid: pBacPak-zGFRα1a^1-35n3^-3C-Tev-ProteinA	This paper	N/A
Plasmid: pBacPak-zRET^1-619^-3C-TEV-ProteinA	This paper	N/A
Plasmid: pBacPak-zRET^1-504^(N259Q, N308Q, N309Q, N433Q) -3C-TEV-ProteinA	This paper	N/A

**Software and algorithms**		

NanoTemper analysis software	NanoTemper	v1.2.231
GraphPad Prism	GraphPad Software Inc.	http://www.graphpad.com/scientific-software/prism/
DIALS	Waterman et al., 2016, Winter et al., 2018	https://dials.github.io/
iMosflm & SCALA	Winn et al., 2011	www.ccp4.ac.uk/
PHASER	McCoy et al., 2007	http://www.phaser.cimr.cam.ac.uk/
PHENIX	Adams et al., 2010	http://www.phenix-online.org/
Coot	Emsley et al., 2010	http://www2.mrc-lmb.cam.ac.uk/Personal/pemsley/coot/
RELION	Scheres, 2012, Kimanius et al., 2016, Zivanov et al., 2018	https://github.com/3dem/relion
Scipion	de la Rosa-Trevín et al., 2016	http://scipion.i2pc.es/
Xmipp	de la Rosa-Trevín et al., 2013	http://scipion.i2pc.es/
Gautomatch	K. Zhang, MRC LMB	https://www.lmb.cam.ac.uk/kzhang/
CryoSparc	Structa Biotechnology Inc. Punjani et al., 2017	https://cryosparc.com/
3DFSC	Tan et al., 2017	https://3dfsc.salk.edu/, https://github.com/LyumkisLab/3DFSC
PyMOL	DeLano Scientific LLC	http://www.pymol.org/
Chimera	Pettersen et al., 2004	https://www.cgl.ucsf.edu/chimera/
Proteome Discoverer v.2.3 with XlinkX node	Thermo Fisher, Liu et al., 2015, Kao et al., 2011	Cat# OPTON-30795/30799
xiNET	Combe et al., 2015	Crosslinkviewer.org

### Resource availability

#### Lead contact

Further information and requests for resources and reagents should be directed to and will be fulfilled by the Lead Contact N.Q.M (neil.mcdonald@crick.ac.uk).

#### Materials availability

The study did not generate new unique reagents.

#### Data and code availability

The coordinates for the zRET-CLD(1-4), zGDNF-zGFRα1a and zRGα1a are available in the PDB with the primary accession code 7AMK, 7AB8 and 7AML, respectively. The zRGα1a C2 symmetry applied map, the zRGα1a symmetry expanded map and the hR15AL negative stain envelopes are available on the EMDB with accession codes EMD-11822 and EMD-11777, respectively.

### Experimental model and subject details

Expi293 cells were used in this study and were grown in suspension in Freestyle 293 Expression media. SF21 and Hi Five insect cells were also used in this study and were grown in serum-free media. Finally Drosophila S2 cells were used in this study and were grown in Ex-Cell420 medium. Additional details are provided in the Method Details section.

### Method details

#### Zebrafish RET CLD(1-4) expression and purification

Zebrafish RET^1-504^ (zCLD(1-4)^red.sug.^) was designed with glycosylation site mutations N259Q, N308Q, N390Q and N433Q to aid in crystallisation. This construct was cloned into a pBacPAK-LL-vector together with a 3C-cleavable C-terminal Protein A tag. A recombinant baculovirus was prepared using the FlashBAC system (2B Scientific). For protein production, SF21 cells were grown to a cell density of 1×10^6^ and incubated with recombinant virus for 112 hours at 27°C. The media was harvested and incubated with IgG sepharose (Sigma), with 1 ml of resin slurry to 1 l of media, whilst rolling at 4°C for 18 hrs. The resin was recovered and washed with 5 column volumes (c.v.) of 20 mM Tris (pH 7.5), 200 mM NaCl, 1 mM CaCl_2_ then incubated with 1:50 (w/w) PreScission Protease (GE Healthcare) for 18 hrs at 4°C. The eluted zCLD(1-4)^red.sug.^ was further purified using a SuperDex 200 (GE Healthcare).

#### zCLD(1-4)^red.sug.^ crystallisation and X-ray data collection

The purified zCLD(1-4)^red.sug.^ was concentrated to 12 mg/ml. Vapour diffusion drops were set up with 2 μl of protein and 2 μl of precipitant; 50 mM MES (pH 6.2), 31.5 % PEG MME 350 (v/v), against 90 μl of precipitant. After 24 hrs of equilibration seeding was performed using Crystal probe (Hampton Scientific). Crystals grew over 14 days at which point they were harvested and flash frozen in liquid nitrogen.

#### zCLD(1-4)^red.sug.^ x-ray data processing and structure determination

Data from these crystals was collected at the Diamond Light Source, initially on beamline I04 and finally on beamline I03. The data was processed with XIA2 utilising DIALS (Winter et al., 2018), before further processing through STARANISO (Tickle et al., 2018) for anisotropy correction to give a 2.08 Å dataset (cut to 2.20 Å for refinement owing to low completeness in the outer shells). Crystals belonged to the triclinic space group P1 with cell dimensions shown in Table 1. Molecular replacement was used as implemented in PHASER (McCoy et al., 2007) to initially locate two copies of CLD1-2 (PDB code 2X2U). The positions of the two associated copies of CLD4 were then determined, utilising an ensemble of the following seven models (superposed by secondary structure matching in COOT): 1L3W (resid A 6-99)(Boggon et al., 2002), 1NCI (resid A 6-99)(Shapiro et al., 1995), 1OP4 (resid A 40-123)(Koch et al., 2004), 4ZPL (resid A 206-314)(Rubinstein et al., 2015), 4ZPM (resid B 207-317)(Rubinstein et al., 2015), 4ZPO (resid A 205-311)(Rubinstein et al., 2015) and 4ZPS (resid A 205-313)(Rubinstein et al., 2015). Initial refinement with PHENIX.REFINE was followed by automated model building with PHENIX.AUTOBUILD (Terwilliger et al., 2007) which completed most of the two polypeptide chains present. Cycles of manual model building with COOT and refinement with PHENIX.REFINE (Afonine et al., 2012) followed. Insect cell glycosylation sites were modelled and checked using PRIVATEER (Agirre et al., 2015), with additional libraries, describing the linkages between monomers generated, and used initially in refinement to maintain a reasonable geometry.

#### zGDNF^mat.^-zGFRα1a^D1-3^ expression and purification

Baculoviruses for zebrafish GFRα1a^1-353^ (zGFRα1a^D1-3^) and zebrafish GDNF^135-235^ (zGDNF^mat.^) were produced using the pBacPAK-LL-zGFRα1a^D1-3^-3C-ProteinA construct and the pBacPAK-LL-melittin^1-20^-zGDNF^mat.^-3C-ProteinA respectively and FlashBacGold viral DNA (2B Scientific) using standard protocols (2B Scientific). Recombinant baculoviruses producing either zGDNF^mat.^ or zGFRα1^D1-3^ were used with SF21 insect cells. The protein was expressed one of two methods. (1) 6 x 2 L flasks containing 500 ml of SF21 cells grown to a cell density of 1 x 10^6^ in SFIII media (Gibco, ThermoFisher), were each infected with 10 ml of the zGDNF_mat._ baculovirus stock and 2 ml of the zGFRα1^D1-3^ baculovirus stock for 86 hrs at 27°C. (2) 4 x 2L flasks containing 300 ml of SF21 cells grown to a cell density of 5 x 10^6^ in SFIII media, were each infected with 30 ml of the zGDNF^mat.^ baculovirus stock and 6 ml of the zGFRα1^D1-3^ baculovirus stock, with 12 ml of yeastolate (50 x stock, Sigma Aldrich), 12 ml lactalbumin (50 x stock, Sigma-Aldrich) and 6 ml glucose (5 M) for 86 hrs at 27°C. Cells were pelleted at 3500 xg and the media containing the secreted 2:2 zGFRα1a^D1-3^-zGDNF^mat.^ complex was pooled. A 1 ml slurry of IgG sepharose resin (GE Healthcare) was added to 1 l of media and incubated at 4°C for 18 hrs. The resin was recovered and washed with 5 column volumes of 20 mM Tris (pH 7.0), 150 mM NaCl and 1 mM CaCl_2_, resuspended in 2 column volumes of the same buffer and incubated with GST-3C (20 μl at 8 mg/ml) for 16 hours. zGDNF^mat^_._-zGFRα1^D1-3^ was further purified using size exclusion chromatography using a Superdex 200 (16/600) (GE Healthcare) in 20 mM Tris (pH 7.0), 100 mM NaCl and 1 mM CaCl_2_.

#### zGDNF^mat.^-zGFRα1a^D1-3^ crystallisation and structure determination

Purified zGDNF^mat.^-zGFRα1^D1-3^ was concentrated to 2.5 mg/ml. 100 nl of protein was dispensed with 100 nl of precipitant onto sitting well trays (MRC-2 drop trays) which comprised 100 mM Tris (pH 8.0), 5 % (w/v) PEG 20,000, 3.7 % (v/v) acetonitrile and 100 mM NaCl. A volume of 90 μl of precipitant solution was dispensed into the well and the trays were then incubated at 22°C. Crystals of zGDNF^mat.^-zGFRα1^D1-3^ formed after 30 days. Crystals were harvested after 55 days and frozen in liquid N_2_ with 30 % (v/v) ethylene glycol used as a cryo-protectant. Data was collected on I04 at Diamond using PILATUS 6M Prosport+ detector. The X-ray diffraction data collected was reduced and integrated using DIALS (Waterman et al., 2016; Winter et al., 2018) at the Diamond Light Source. The structure was phased by molecular replacement in PHASER (McCoy et al., 2007) and in CCP4 (The CCP4 Suite, 1994; Winn et al., 2011) using the human GDNF-GFRα1 starting model (PDB 3FUB) (Parkash & Goldman (2009)). Model refinement was performed using COOT (Emsley and Cowtan, 2004; Emsley et al., 2010) and PHENIX.REFINE (Adams et al., 2010; Afonine et al., 2012) against the dataset that was reduced and integrated using the STARANISO (Tickle et al., 2018) at a resolution of 2.2 Å. Glycosylation sites were validated using PRIVATEER (Agirre et al., 2015).

#### zRET^ECD^-zGDNF^mat.^-zGFRα1a^D1-3^- (zRGα1a) complex expression and purification

A recombinant baculovirus was prepared to produce zRET^ECD^ (residues 1-626) using the pBacPAK-LL-zRET^ECD^-3C-Protein A construct and FlashBac viral DNA (2B Scientific) using standard protocols and as described above. To produce zRET^ECD^ either one of two separate protocols were used; (1) SF21 insect cells grown using SFIII media in 6×500 ml flasks to a cell density of 1×10^6^ were then infected with 2 ml of the baculovirus that contained zRET^ECD^ for 86 hrs at 27°C, (2) 4 x 2L flasks containing 300 ml of SF21 cells grown to a cell density of 5.5 x 10^6^ in SFIII media, were each infected with 6 ml of the zRET^ECD^ baculovirus stock, with 12 ml of yeastolate (50 x stock, Sigma-Aldrich), 12 ml lactalbumin (50 x stock, Sigma-Aldrich) and 6 ml glucose (5 M) for 86 hrs at 27°C. Cells were pelleted at 3500 g and the media containing secreted zRET^ECD^ was pooled and 1 ml of IgG sepharose resin (GE Healthcare) was added to 1 l of media and incubated at 4°C for 18 hrs. The resin was recovered and washed with 5 column volumes of 20 mM Tris (pH 7.0), 150 mM NaCl and 1 mM CaCl_2_, then resuspended in 2 column volumes of the same buffer. Purified 2:2 zGFRα1a^D1-3^-zGDNF^mat.^ complex was then added directly. The sample was incubated for 45 min at 4°C. The resin with the zRGα1a complex was then recovered and washed with 5 c.v. of 20 mM Tris (pH 7.0), 150 mM NaCl and 1 mM CaCl_2_ buffer, resuspended in 2 column volumes of buffer and incubated with GST-3C (20 μl at 8 mg/ml) for 18 hours at 4°C. The eluted zRGα1a complex was further purified using size exclusion chromatography using a Superdex 200 (16/600) (GE Healthcare) in 20 mM HEPES (pH 7.0), 150 mM, NaCl and 1 mM CaCl_2_.

To prepare a cross-linked sample, 100 μl of purified zRGα1a (4 mg/ml) was applied on top of a 5-20 % (w/v) sucrose gradient which contained a 0-0.1 % (v/v) glutaraldehyde gradient, the gradient was buffered with 20 mM HEPES (pH 7.0), 150 mM NaCl and 1 mM CaCl_2_. Ultracentrifugation was performed at 33,000 r.p.m (SW55 rotor) for 16 hours at 4°C. The sucrose gradient was fractionated in 125 μl fractions, the glutaraldehyde was quenched with 1 M Tris (pH 7.0), to a final concentration 100 mM. The fractions that contained cross-linked zRGα1a were pooled and further purified by Superdex200inc 10/300 (GE Healthcare) in a buffer of 20 mM Tris (pH 7.0), 150 mM NaCl and 1 mM CaCl_2_, in order to remove the sucrose from the crosslinked zRGα1a complex.

#### zRGα1a cryo-electron microscopy sample preparation

To prepare cryo-EM grids, 1.2/1.3 300 mesh Cu Quantifoil™ grids 300 mesh grids were glow discharged using 45 mA for 30 s using a Quorum Emitech K100X. For the untilted dataset (Dataset 1), 4 μl of crosslinked zRGα1a sample, at 0.1 mg/ml, was applied to the grids, using a Vitrobot Mark IV (Thermo Fisher) with the parameters; 90 s wait time, 5 s blot time at 22°C with 100 % humidity. The same glow discharge parameters were used for the grids for the tilted dataset (dataset 2), 4 ul was applied to the grid at 4 ˚C and a 20 s wait with 3 s blot time under 100 % humidity. For the non-crosslinked zRGα1a sample, the same glow discharge parameters were used for 1.2/1.3 300 mesh Cu Quantifoil™ grids 300 mesh grids. 4 μl of non-crosslinked zRGα1a at 0.1 mg/ml was applied to the grids with the same parameters as those used for the grids prepared for dataset 1, these grids were used for dataset 3.

#### Cryo-EM data acquisition: Datasets 1 to 3

Frozen-hydrated grids of the crosslinked zRGα1a sample were imaged on a Titan Krios electron microscope (Thermo Fisher) operating at 300 kV at the Francis Crick Institute. Movies were captured on a BioQuantum K2 detector (Gatan) in counting mode at 1.08 Å/pixel and with an energy filter slit width of 20 eV. Dataset 1 was collected with a 0° tilt angle, a defocus range of 1.4-3.5 μm and comprised a total of 6105 movies. For dataset 2, 6375 movies were collected in total using a tilt angle of 30° and the same defocus range used for dataset 1. Movies from datasets 1 and 2 had an exposure of 1.62 e^-^/Å^2^ per frame for a total electron exposure of 48.6 e^-^/Å^2^. The dose rate was 6.4 e^-^/pixel/sec and exposure time was 9 seconds/movie. For dataset 3, frozen-hydrated grids of non-crosslinked zRGα1a were collected on a Talos Arctica microscope (Thermo Fisher) operating at 200 kV at the Francis Crick Institute. A total of 1705 movies were captured on a Falcon 3 detector in integrating mode at 1.26 Å/pix and a defocus range of 1.5-3.0 μm. Movies from dataset 3 had an exposure of 6.07 e^-^/Å^2^ per frame which led to a total exposure of 60.66 e^-^/Å^2^. All datasets were collected using EPU version 1.9.0 (Thermo Fisher).

#### Cryo-EM data processing of crosslinked zRGα1a (dataset 1)

MotionCorr2 (Zheng et al., 2017) was used to correct for motion in the movie frames in Scipion 1.2 (de la Rosa-Trevín et al., 2016). The contrast transfer function was estimated using CTFfind4.1(Rohou and Grigorieff, 2015). 5855 micrographs were selected from dataset 1 and initial particle picking was performed with RELION-2.1 manual picking, 4899 particles were extracted with RELION-2.1 (Kimanius et al., 2016) particle extract function (de la Rosa-Trevín et al., 2016) with a box size of 340 and binned two-fold. 2D classification was performed using RELION 2D classification, with 20 initial classes. Six classes were used to pick a subset of 3000 micrographs using RELION-2.1 autopicking in Scipion 1.2, giving 638,000 particles with box size 340, binned 2 fold. These were classified using 2D classification in RELION-2.1. Twelve classes were selected for picking using Gautomatch [K. Zhang, MRC LMB (www.mrc-lmb.cam.ac.uk/kzhang/)] to pick 2,424,600 particles, which were extracted with a box size of 340 pixels and binned 2-fold using RELION-2.1 2D class averaging was performed in CryoSPARC-2 (Punjani et al., 2017) leading to 1,156,517 particles which were extracted using RELION-2.1 (Kimanius et al., 2016; Scheres, 2012) with a box size of 320 pixels.

#### Cryo-EM data processing of crosslinked zRGα1a (tilted dataset 2)

Dataset 2 was processed and corrected for motion correction and CTF estimation as described above. A total of 4848 micrographs were used to pick particles semi-automatically with Xmipp and 69,386 particles were extracted with a box size of 360 pixels using RELION-2.1 (Kimanius et al., 2016; Scheres, 2012) in Scipion1.2 (de la Rosa-Trevín et al., 2016). 2D classification was then performed using RELION automatic picking leading to 1,183,686 particles being extracted using RELION-2.1 (Kimanius et al., 2016; Scheres, 2012; Zivanov et al., 2018) with a box size of 340 binned 2-fold. Subsequent 2D classification in RELION-2.1 (Kimanius et al., 2016; Scheres, 2012) lead to 12 classes which were used by Gautomatch [K. Zhang, MRC LMB (www.mrc-lmb.cam.ac.uk/kzhang/)] to pick 1,393,023 particles. The particles were extracted with RELION-2.1 (Kimanius et al., 2016; Scheres, 2012) with a box size 320, 2-fold binned, were imported into CryoSPARC-2 (Punjani et al., 2017) and 2D classification generated 208,057 particles from 3175 micrographs. These particles were re-extracted with a box size of 320 and per-particle CTF estimation was performed using GCTF (Zhang, 2016).

#### Combining and processing cryo-EM datasets 1 and 2 for crosslinked zRGα1a

Dataset 1 and 2 were combined and an initial 2D classification was performed in CryoSPARC-2 on the 1,364,574 particles (Afonine et al., 2018). Following this, 1,242,546 particles underwent two heterogeneous refinements using 5 classes with strict C2 symmetry applied in CryoSPARC-2 (Punjani et al., 2017) lead to a homogeneous refinement with 468,922 particles. Once re-imported into Scipion1.2, RELION 2D class averaging was implemented to generate 364,158 and 22,358 particles from dataset 1 and dataset 2, respectively (Kimanius et al., 2016; Scheres, 2012). Particle polishing was performed in RELION-2.1 (Kimanius et al., 2016). Once imported into CryoSPARC-2, 2D class averaging removed any further particles, yielding 382,547 particles used for a homogeneous refinement followed by a non-uniform refinement with C2 symmetry applied. This final reconstruction gave a resolution of 3.3 Å as calculated using the ‘gold’ standard (FSC=0.143) (Punjani et al. 2017). Symmetry expansion was performed in RELION-2.1 and 3D-refinement with masking was performed with no symmetry applied (Kimanius et al., 2016; Scheres, 2012). Postprocessing in RELION-2.1 of the final symmetry expanded reconstruction with a resolution 3.5 Å (Figure S4) (Kimanius et al., 2016; Scheres, 2012).

#### Building the zRGα1a complex into the final cryo-EM map

To build a full ligand-co-receptor complex, the zGDNF^mat.^-zGFRα1^ΔD1^ crystal structure described here was used together with a homology model of domain D1 (zGFRα1^29-121^) generated by MODELLER from the GFRα2-neurturin crystal structure (PDB 5MR4) (Sandmark et al., 2018; Webb and Sali, 2016). For zRET, chain A of the CLD(1-4) module described here was used together with a CRD model generated with SwissPROT (Schwede et al., 2003) using the structure of hRET^ECD^ in complex with GFRα2-neurturin (PDB 6Q2O)(Li et al., 2019; Webb and Sali, 2016). The zGDNF-zGFRα1 and zRET^ECD^ structures were then docked into the symmetry expanded map using PHENIX (Adams et al., 2010). The model was refined at 4.2 Å against the sharpened map using PHENIX_REAL_SPACE_REFINE (Afonine et al., 2018) and manual model building and model refinement was done in COOT (Emsley and Cowtan, 2004; Emsley et al., 2010). The final symmetry expanded model was used to generate the 2:2:2 zRGα1a model, which was placed in the C2 averaged map using PHENIX (Adams et al., 2010) using PHENIX_REAL_SPACE_REFINE (Afonine et al., 2018). Glycosylation sites were validated using PRIVATEER (Agirre et al., 2015). Protein-protein interface areas were calculated using PDBePISA (Krissinel and Henrick, 2007). All images of maps were produced in Chimera (Pettersen et al., 2004) and structure-based figures were rendered in PyMOL (Schrodinger, 2015).

#### Cryo-EM data processing for a non-crosslinked zRGα1a sample (dataset 3)

MotionCorr2 (Zheng et al., 2017) was used to correct for motion in the movie frames in RELION-3 (Zivanov et al., 2018). The contrast transfer function was estimated using CTFfind4.1 (Rohou and Grigorieff, 2015). 384 micrographs were selected from and initial particle picking was performed with RELION-3 manual picking, 951 particles were extracted with RELION-3 (Zivanov et al., 2018) particles extract with a box size of 320 and binned 2 fold. 2D classification was performed using RELION-3 2D classification, with 10 initial classes ( Zivanov et al., 2018). One class, due to the orientation bias, was selected and used by RELION autopick to pick from a subset of 81 micrographs. This gave 19,715 particles picked and extracted with a box size of 320 pixels using RELION-3. These particles were sorted in RELION-3 and 15,519 were then were classified using RELION 2D classification. A total of 11070 particles were used from 81 micrographs to explore the linear particle arrays observed for the zRGα1a complex.

For the 2D classification of the isolated zRGα1a particle pairs initial picking performed with RELION-3 manual picking yielded 239 particles that were extracted with a box size of 420 pixels. 2D classification was performed and one class was used to for RELION autopick, yielding 4567 particles that were extracted using RELION extract with a box size of 400 pixels from 81 micrographs. RELION 2D classification produced the final 2D of the isolated zRGα1a pair with 1194 particle pairs (2388 individual particles) (Zivanov et al., 2018).

#### Analysis of zRGα1a multimer formation on cryo-EM grids

Following 2D class averaging in RELION-3, the final 11070 particles were repositioned onto 81 micrographs collected from cryo-grids prepared from the non-crosslinked zRGα1a sample using RELION particle reposition. A Python script was written to extract the particle number, psi angle (ψ) and Cartesian coordinates of particle pairs from the 2D class average STAR file. Particle pairs were detected through analysing each single particle and locating surrounding particles within 214.2 Å (170 pixels), using their extracted Cartesian coordinates. A subset of 14 micrographs was used, where a total of 3756 individual particles lead to 4132 particle pairs. The distance between each particle pair was determined using their X and Y coordinates. The ψ angles were corrected to positive integers, and were permitted to be within the 180° range due to the C2 symmetry of the complex. The difference between the two positive ψ angles from the particle pairs (Δψ) was calculated as an absolute value. Distance between the particles and the Δψ between particle pairs was calculated and plotted on a 3D surface plot with the bins every 2 Å and every 2.6°, respectively.

#### Human RET^ECD^ expression and purification

A codon-optimised human RET^ECD^ (hRET^ECD^) cDNA encoding residues 1-635 followed by a TEV-cleavable Avi and C-tag was cloned into a pExpreS2.1 vector (ExpreS2ion Biotechnologies, Hørsholm, Denmark) with Zeocin resistance. A stable pool of S2 cells, secreting hRET^ECD^, was generated by transfecting 25 ml of S2 cells grown in Ex-Cell420 medium (Sigma) with 10 % (v/v) FBS at a density of 5×10^6^ cells/ml using 12.5 μg of DNA and 50 μl of ExpresS^2^-Insect TR (5×). Stably transfected cells were selected with 2 mg/ml Zeocin with repeated medium exchange. The culture was expanded to 1 litre in a 5L glass-flask and the supernatants collected after 7 days.

For purification, 1 ml of C-tag capture resin (ThermoFisher) was added to a cleared and filtered S2 supernatant and incubated for 18 hrs at 4°C. The resin was pelleted and washed several times with PBS before eluting bound hRET^ECD^ by competition with PBS containing 200 μg/ml SEPEA peptide. At this point, the affinity and biotinylation tags were removed by digestion with TEV (a 1:10 ratio of TEV protease:RET). The purified hRET^ECD^ was further purified by size-exclusion using a Superdex200 10/300 with a 50 mM Tris (pH 7.5), 100 mM NaCl buffer.

#### Human GDF15^mat.^-GFRAL^D1-3^ complex expression and purification

Both human GFRAL^21-352^ (referred to hereafter as hGFRAL^D1-3^) and hGDF15^198-308^ (referred to hereafter as hGDF15^mat^^.^) were cloned into a pCEP vector with an N-terminal BM40 secretion sequence. The hGFRAL construct had a C-terminal 6 His tag. The constructs were co-transfected into Expi293 cells (Life Tech) using polyethylimine. The transfected cells were incubated in Freestyle media at 37°C, 8 % CO_2_ with 125 rpm shaking. Conditioned media was harvested after 5 days, and Tris pH 8.0 and imidazole added to a final concentration of 10 mM and 20 mM respectively. The media was incubated with Ni-NTA agarose beads whilst rolling at 4°C for 2 hours. The beads were recovered and washed with 20 mM Tris (pH 7.4), 137 mM NaCl and the protein was eluted with 20 mM HEPES (pH 7.4), 137 mM NaCl and 500mM imidazole. The protein was concentrated to ~5 mg/ml. This protein was further purified by Superdex 200 increase size exclusion chromatography in buffer 20 mM HEPES (pH 7.4), 137 mM NaCl to give a pure 2:2 GDF15-GFRAL complex.

#### hRET^ECD^-hGDF15^mat.^-hGFRAL^D1-3^ (hR15AL) complex assembly and purification

An excess of purified hRET^ECD^ (300 μl, 1.1 mg/ml) was incubated with purified hGDF15-hGFRAL (300 μl, 0.75 mg/ml) for 1 hr whilst mixing at 4°C in the presence of 10-fold excess heparan sulfate DP-10 (20 μM) (Iduron, UK). The hR15AL complex was further purified by size exclusion chromatography using a Superdex 200 increase in to 20 mM HEPES (pH 7.0), 150 mM NaCl and 1 mM CaCl_2_. For sample crosslinking, 100 μl of the hR15AL complex (0.75 mg/ml) was applied on top of a 5-20 % (w/v) sucrose gradient which contained a 0-0.1 % (v/v) glutaraldehyde gradient, the gradient was buffered with 20mM HEPES (pH 7.0), 150 mM NaCl and 1 mM CaCl_2_. Ultracentrifugation was performed at 33,000 rpm for 16 hours at 4°C. The sucrose gradient was fractionated in 125 μl fractions, the glutaraldehyde was quenched with 1M Tris (pH 7.0), to a final concentration 100 mM. The fractions were assessed using SDS-PAGE and fractions that contained the complex were used for negative stain.

#### hR15AL negative stain preparation, data acquisition and processing

Cu 200 mesh carbon coated grids were glow discharged under vacuum using 45 mA for 30 s. A sample of 4 μl of the crosslinked hR15AL undiluted from the GraFix column was applied to the charged grid and left for 30 s and the excess removed by blotting and placing the grid, sample side facing the solution, in 10 μl of 2 % (w/v) uranyl acetate solution in d.H_2_O, and blotting immediately twice, followed by placing the grid in the 3^rd^ 10 μl drop sample side facing down and leaving it in solution for 1 min, followed by a final blot until almost all the solution has been wicked off. The grid was then left to dry for 5 mins.

Micrographs were collected on a BMUltrascan 1000 2048x2048 CCD detector using a Tecnai Twin T12 (Thermo Fisher) at 120 kV with a defocus range of 1-1.5 μm and with a 1 s exposure time. A total of 299 micrographs were collected and particles were picked using Xmipp (de la Rosa-Trevín et al., 2013) semi-automated picking, in Scipion1.2 (de la Rosa-Trevín et al., 2016). This gave 27,551 particles were extracted with RELION-2.0 particle extraction (Kimanius et al., 2016; Scheres, 2012). 2D class averaging was performed with RELION-2.0 (Kimanius et al., 2016; Scheres, 2012). The resulting 16,159 particles were used to generate an initial model using RELION 3D ab-initio model. 3D classifications with 5 classes were performed using RELION-2.0 3D classification (Kimanius et al., 2016; Scheres, 2012). 6519 particles were taken forward into the final reconstruction a resolution of 25.8 Å using RELION-2.0 3D refinement (Kimanius et al., 2016; Scheres, 2012). The data processing was done in Scipion1.2 (de la Rosa-Trevín et al., 2016).

#### Microscale thermophoresis (MST) measurement of zRET^ECD^ binding affinity

MST measurements were performed at 25°C in 20 mM HEPES (pH 7.0), 150 mM NaCl, 1 mM CaCl_2_ and 0.05 % (v/v) Tween-20 using a Nanotemper Monolith NT.115 (Nanotemper). To measure the affinity of zGFRα1^D1-3^-zGDNF^mat.^ towards zRET^ECD^; zRET^ECD^ was labelled with NHS-RED 2^nd^ generation dye (Amine Reactive) using the labelling kit (Nanotemper). A 1:1 serial dilution of unlabelled zGFRα1^D1-3^-zGDNF^mat.^ (WT and mutants) was performed. The samples were incubated with the labelled zRET^ECD^-NHS-RED (50 nM, fluorophore, 83.7 nM zRET^ECD^) for 10 mins at 22°C. Hydrophobic treated capillaries were filled with the serially diluted samples (Nanotemper). The MST run was performed using a Monolith 1.115 with the LED power and MST both set to 20 %, with a measurement time of 20 sec. To measure the affinity of zGFRα1^D1-3^-zGDNF^mat.^ towards zRET^ECD^_P291-Q296;AAG_; zRET^ECD^_P291-Q296;AAG_ was labelled with NHS-RED 2^nd^ generation dye (Amine Reactive) using the labelling kit (Nanotemper), and the procedure was carried out as above with zRET^ECD^_P291-Q296;AAG_-NHS-RED (50 nM, fluorophore, 80.7 nM zRET^ECD^).

#### Surface conservation analysis and heatmaps for different GFL-GFR ligand-coreceptor pairs

The sequence for the globular domains of zGFRα1a (Uniprot Q98TT9) was aligned to hGFRα1 (Uniprot P56159), hGFRα2 (Uniprot O00451), hGFRα3 (Uniprot O60609), hGFRα4 (Uniprot Q9GZZ7), and hGFRAL (Uniprot Q6UXV0), using Clustal Omega.(Sievers et al., 2011) The sequence of the mature zGDNF (Uniprot Q98TU0) was aligned to hGDNF (Uniprot P39905), hNRTN (Uniprot Q99748), hARTN (Uniprot Q5T4W7), hPSPN (Uniprot O60542), and hGDF15 (Uniprot Q99988) using Clustal Omega (Sievers et al., 2011). Using these alignments, residues were categorised based on residue type and a heat map was generated and values mapped onto a surface representation on the zGFRα1a^D2-D3^. D1 was excluded from the analysis due to the major differences between each of the co-receptors; which is missing hGFRα4 and is located in a completely different position in hGFRAL. Each of the categories for residue type are as follows; aromatic residues (F, W, and Y), aliphatic residues (A, I, L, and V), residues containing an alcohol functional group (S and T), positively charged residues (R and K), negatively charged residues (D and E), and residues containing an amide bond in the side chain (N and Q), and C, G, H and M were counted individually. The sequence similarity was numbered from 0-1, 0 indicating no similarity at all and 1 indicating the residue type was identical between the GFR or GFL family members respectively. The value for each residue in the sequence were represented as a surface colour coded with the highest residue similarity in red (1) through yellow (0.5) to white (0).

### Quantification and statistical analysis

Binding kinetics were derived from the MST binding curves using the NanoTemper analysis software version 1.2.231, with each point determined by averaging data obtained between 10 and 15 sec on the MST curve for each capillary. Fractional binding values from an entire concentration range were derived by normalising the values from 0 to 1 in Microsoft Excel, with visual inspection to check these concentrations corresponded to the plateau in MST signal at low and high ligand concentrations. Data for at least three such binding experiments were imported into Graphpad Prism 8.0.0 and, due to the proximity of the apparent binding constant and fluorescently-labelled RET receptor concentration, subjected to a non-linear regression fit using a quadratic equation to determine the K_D_.

## Supplemental information

Document S1. Figures S1–S8, Table S1, and Data S1/Methods S1

Document S2. Article plus supplemental information
