# Supplementary material for: A two-site flexible clamp mechanism for RET-GDNF-GFRα1 assembly reveals both conformational adaptation and strict geometric spacing
Source: Structure. 2021 Jul 1;29(7):694–708.e7. doi: 10.1016/j.str.2020.12.012 (PMC8266384; doi:10.1016/j.str.2020.12.012)
Supplement: Document S1. Figures S1–S8, Table S1, and Data S1/Methods S1 [file mmc1.pdf]

Structure, Volume *29*

Supplemental Information

A two-site flexible clamp mechanism for RET-GDNF-GFRα1 assembly reveals both conformational adaptation and strict geometric spacing

Sarah E. Adams, Andrew G. Purkiss, Phillip P. Knowles, Andrea Nans, David C. Briggs, Annabel Borg, Christopher P. Earl, Kerry M. Goodman, Agata Nawrotek, Aaron J. Borg, Pauline B. McIntosh, Francesca M. Houghton, Svend Kjær, and Neil Q. McDonald

SUPPLEMENTARY FIGURES

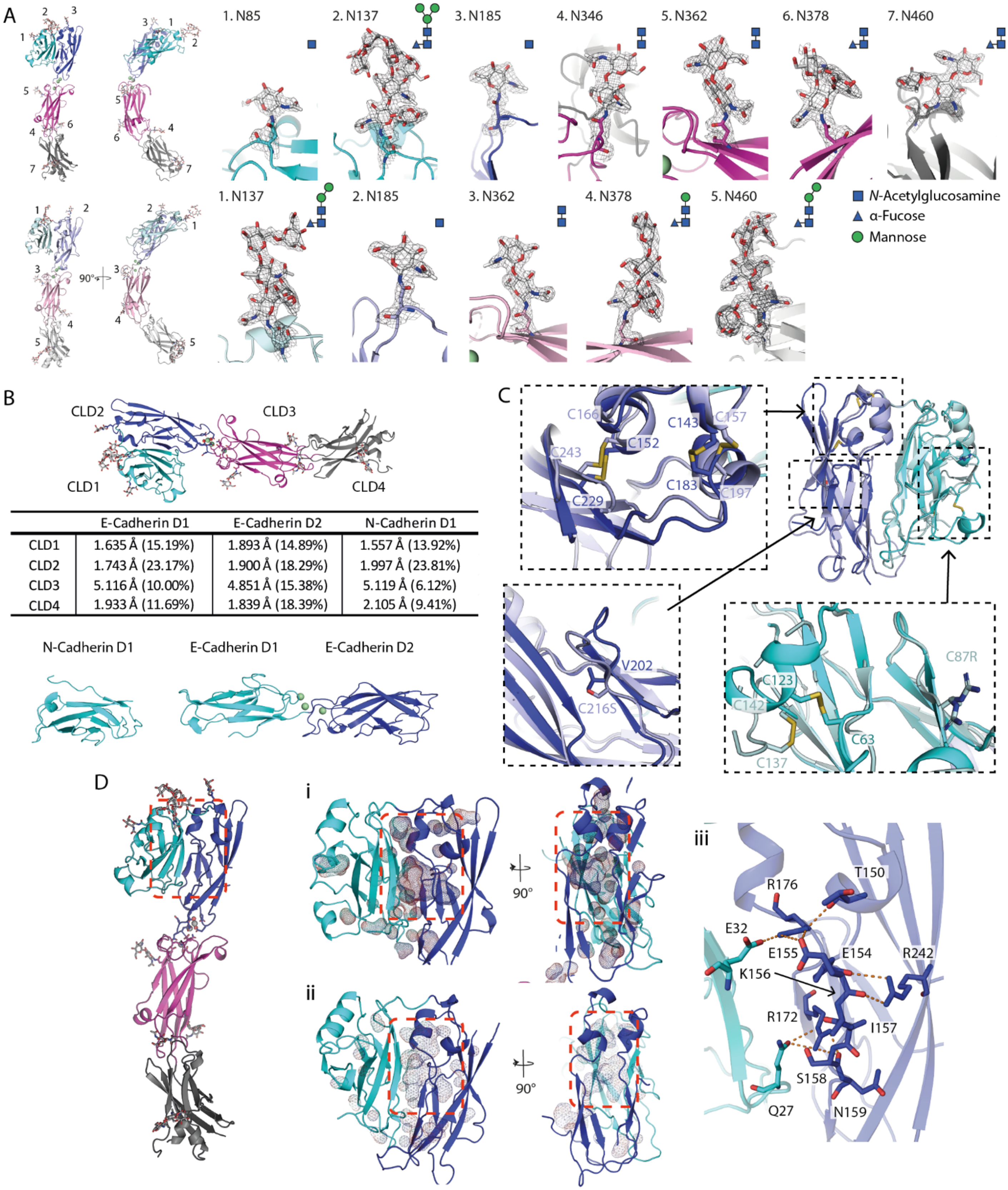

	E-Cadherin D1	E-Cadherin D2	N-Cadherin D1
CLD1	1.635 Å (15.19%)	1.893 Å (14.89%)	1.557 Å (13.92%)
CLD2	1.743 Å (23.17%)	1.900 Å (18.29%)	1.997 Å (23.81%)
CLD3	5.116 Å (10.00%)	4.851 Å (15.38%)	5.119 Å (6.12%)
CLD4	1.933 Å (11.69%)	1.839 Å (18.39%)	2.105 Å (9.41%)

Supplementary Figure 1: Structural analysis of human RET CLD1-4$^{red.Sug.}$ A) Asparagine-linked sites (N-linked) within the zRET^{CLD1-4} module and final electron density map calculated using m2Fo-DFc coefficients contoured at 1.0 σ corresponding to each N-linked glycosylation site are shown. B) Calculated RMSD values (and sequence identity) between each of the cadherin like domains from zCLD1-4$^{red.sug.}$ and E-cadherin domains 1 and 2 (PDB 1EDH)(Nagar et al., 1996) and N-cadherin domain 1 (PDB 1NCI)(Shapiro et al., 1995). C) Disulfide swapping evident between higher and lower vertebrates; zCLD1-2 (in cyan and blue) aligned with the hCLD12 structure (in pale cyan and pale blue, PDB 2X2U), with close-ups of the cysteine within the structure. The two unpaired cysteines unique to hRET were mutated to arginine (C87R) and a serine (C216S) to aid structure determination of hRET^{CLD1-2} (Kjær et al., 2010). D) Analysis of the zCLD1-2 clamshell interface; orthogonal views of the cavity (shown in mesh) within CLD1-2 for both zebrafish (i) and human (ii) (PDB 2X2U)(Kjær et al., 2010). (iii) The residues incorporated into the CLD1-2 clamshell interface; CLD2-β1 stabilised with R242 (CLD2-β6), R172 and R176 (CLD2-β2) the latter of which also interact with Q27 and E32 (CLD1-β1). In all cases the structures are represented as a cartoon and the residues are represented as sticks with individual domains are coloured as follows; CLD1 in cyan, CLD2 in blue, CLD3 in magenta and CLD4 in grey. All images were rendered in PyMOL (Schrodinger, 2015). Related to Figure 1.

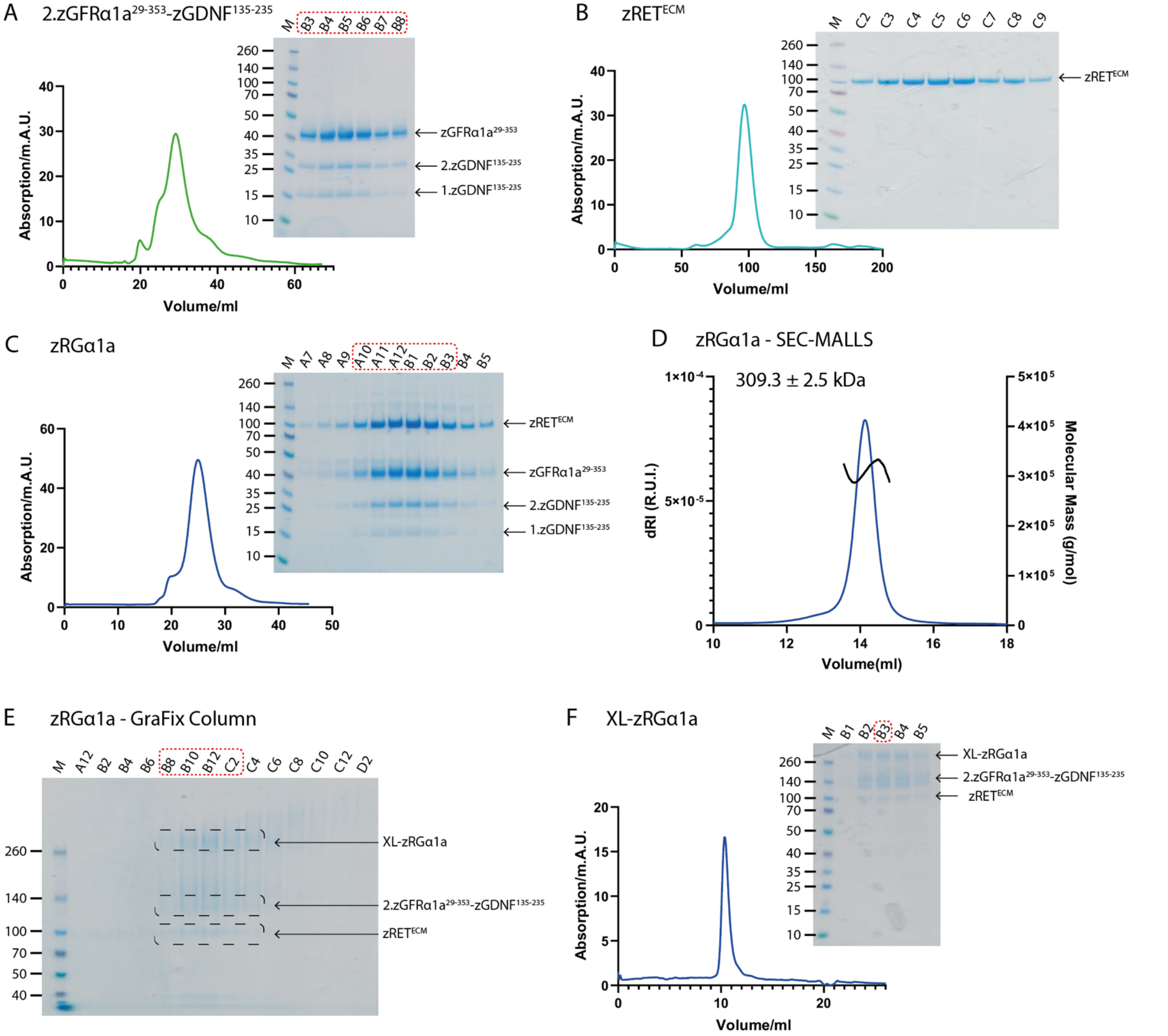

Supplementary Figure 2: Purification of individual zRGα1a components and crosslinking of the zRGα1a ternary complex. Size exclusion profiles of A) 2.zGFRα1a$^{29-353}$-zGDNF$^{135-235}$, B) zRET^{ECD} (shown here as zRET^{ECM} for extracellular module) and C) zRGα1a D) SEC-MALLS trace of the purified zRGα1a E) SDS-PAGE of fractionated GraFix stabilised zRGα1a sample. F) Size exclusion profile of the crosslinked zRGα1a sample.Related to STAR methods.

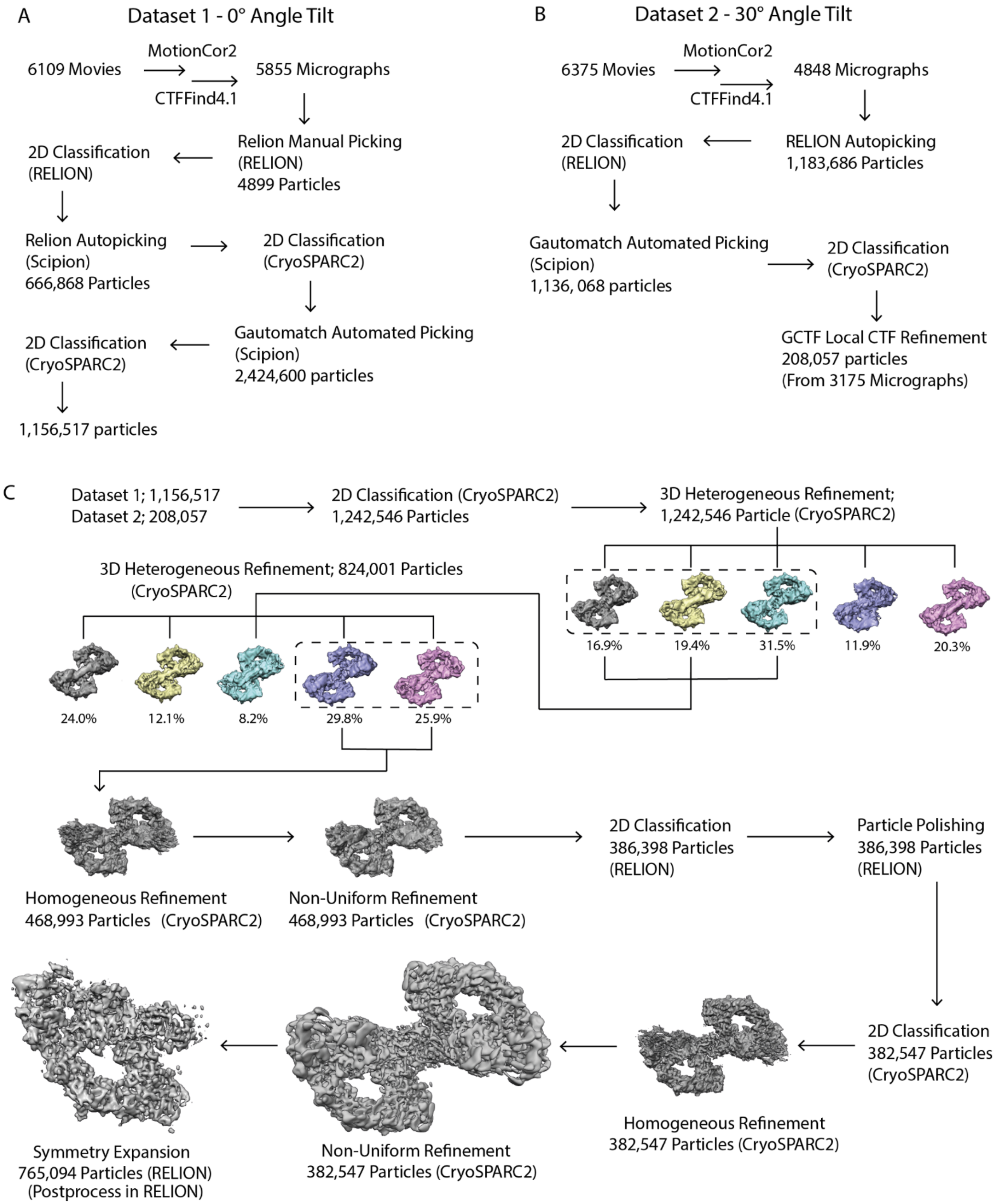

Supplementary Figure 3: zRGα1a cryo-EM data processing workflow. A) Non-tilted particles dataset 1 and B) tilted particles dataset 2 were processed independently. C) Combined particles from both datasets 1 and 2 and workflow. Software packages used; CryoSPARC2 (Punjani et al., 2017), CTFFind4.1 (Rohou and Grigorieff, 2015), Gautomatch [K. Zhang, MRC LMB (www.mrc-lmb.cam.ac.uk/kzhang/)], GCTF (Zhang, 2016), MotionCor2 (Zheng et al., 2017) RELION (Kimanius et al., 2016; Scheres, 2012; Zivanov et al., 2018), Scipion (de la Rosa-Trevín et al., 2016), Related to Figure 2.

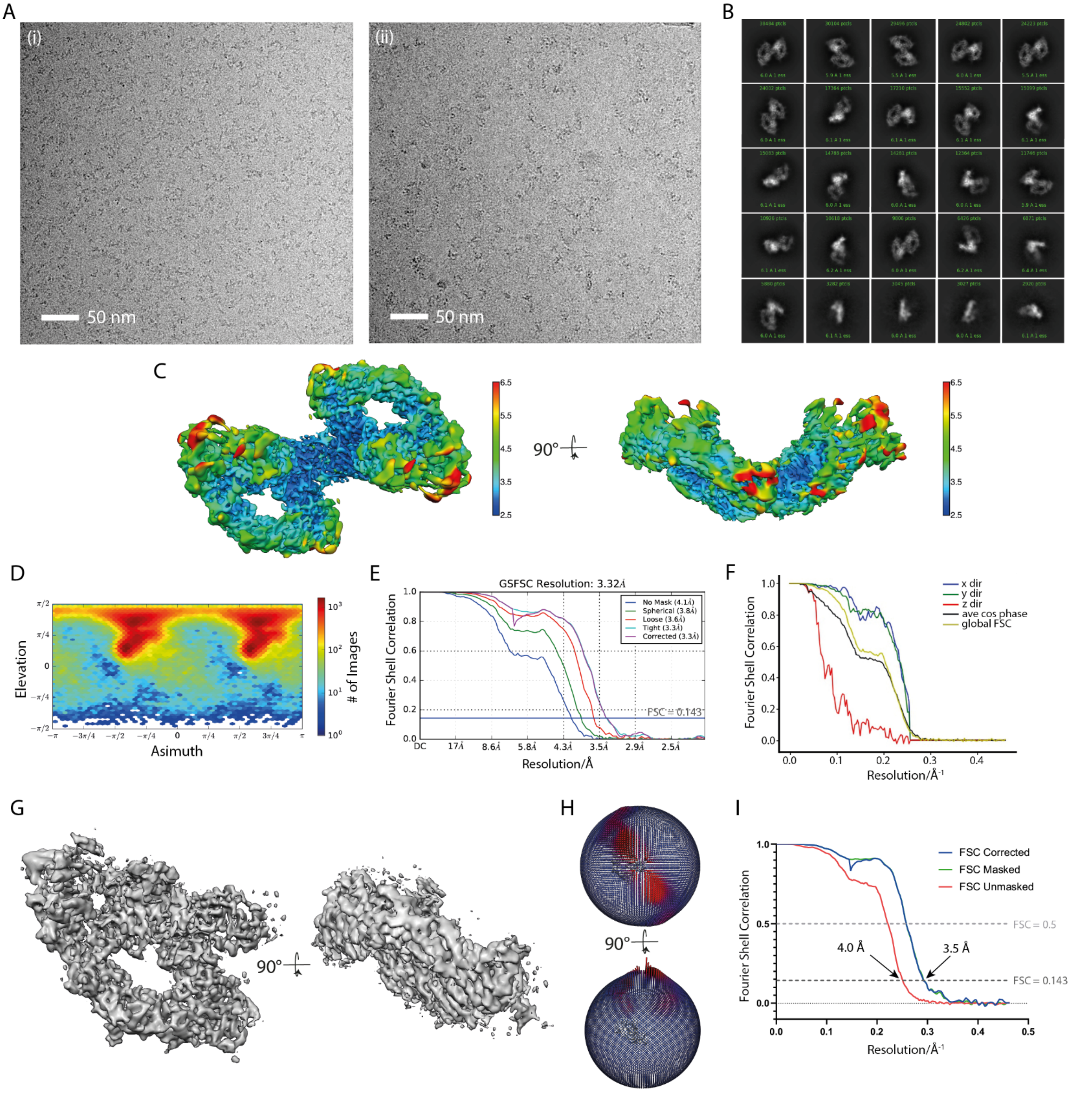

Supplementary Figure 4: zRGα1a single particle cryo-EM reconstruction. A) Representative micrographs from the non-tilted dataset (i) and the dataset collected with a tilt angle of 30° (ii). B) Twenty-five 2D class averages from all the particles in the final reconstruction. C) The C2 averaged cryo-EM map is coloured by local resolution and was generated by the locRes option in CryoSPARC2 (Punjani et al., 2017) using blue for 2.5 Å resolution areas, green for 4.5 Å and red for 6.5 Å resolution. A total of 382,574 particles were generated using CryoSPARC2 (Punjani et al., 2017), non-uniform refinement and postprocessed in RELION (Kimanius et al., 2016; Scheres, 2012; Zivanov et al., 2018). Two orthogonal views of the zRGα1a complex are shown. D) Angular distribution of the particles in the C2 averaged map. E) Fourier shell correlation curve of the C2 averaged map. F) A 3DFSC (Tan et al., 2017) plot generated from the C2 averaged map. G) Two views orthogonal of the symmetry expanded map, generated using particles expansion in RELION (Kimanius et al., 2016; Scheres, 2012; Zivanov et al., 2018). H) Two orthogonal views of the angular distribution from the symmetry expanded map. I) The Fourier shell correlation curves from the symmetry expanded map showing an overall resolution of 3.5 Å. Images of the maps were rendered using Chimera (Pettersen et al., 2004). Related to Figure 2.

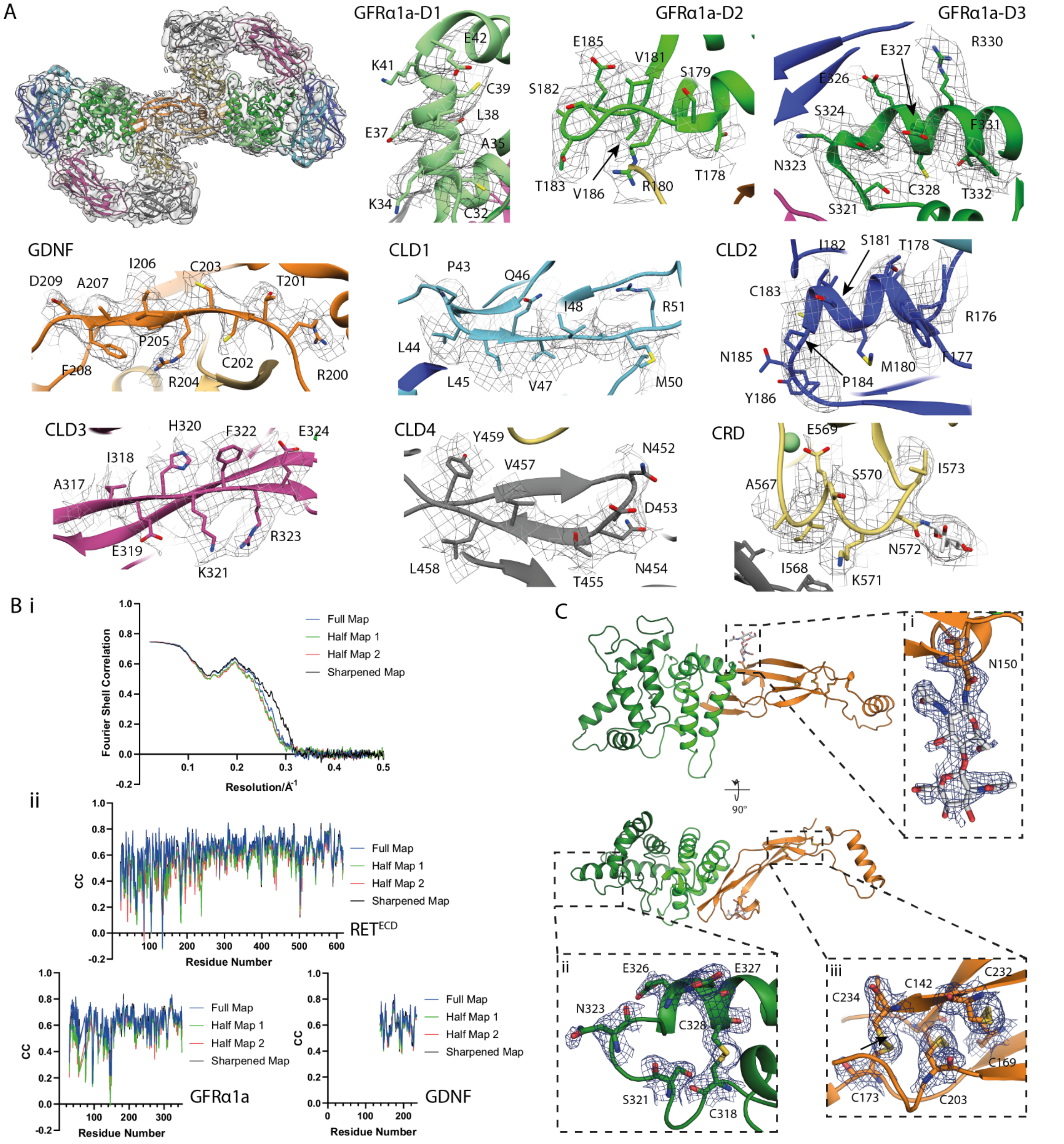

Supplementary Figure 5: Cryo-EM map to zRGα1a model correlation and electron density map quality for zGDNF$^{138\text{-}235}$zGFRα1a$^{151\text{-}353}$ structure A) The C2 averaged map and model with sections of each domain highlighted. The residues are represented as sticks and the mainchain as ribbon, the maps are shown as mesh. B) (i) Map-to-model Fourier shell correlation between the C2 averaged cryo-EM map and the zRGα1a model. (ii) Cross correlation between each residue in the model and the C2 averaged map. Correlation statistics were provided with the use of the full maps and two half maps in each case using Phenix cryo-EM model validation tools (Afonine et al., 2018). C) Final 2.2 Å zGDNF-zGFRα1a structure showing the crystallographic asymmetric unit contains a single copy of zGDNF$^{138\text{-}235}$zGFRα1a$^{151\text{-}353}$. The insets reveal the final electron density calculated for different areas of the structure using m2Fo-DFc coefficients and contoured at 1.0 σ. The glycosylation site located on N150 of GDNF (i), the binding site of zGFRα1a that interacts with RET CLD(2-3) calcium site (ii), and the disulfide bond network in zGDNF (iii). Images were rendered in Chimera (Pettersen et al., 2004) or PyMOL (Schrodinger, 2015).Related to Figure 2.

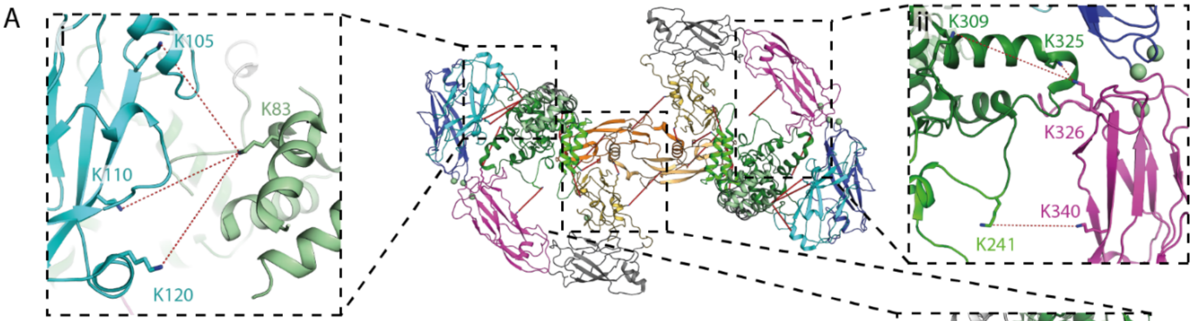

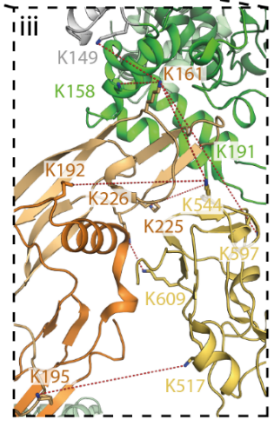

Score	Protein1	PepPos1	PepSeq1	LinkPos1	Protein2	PepPos2	PepSeq2	LinkPos2	
212.03	GDNF	192	KLDKDTPSR	1	RETECD	544	DKGISKR	2	
182.35	GFRa1	309	SPKISVSPFCDCSSSGNSKEECDR	3	RETECD	326	FREEKAIFGNVR	5	
169.05	RETECD	609	AGHGTCYCFEGKCFCER	12	GDNF	226	KHSAK	1	
163.62	GFRa1	325	ISVSPFCDCSSSGNSKEECDRFTEFFTDNACLR	16	RETECD	326	FREEKAIFGNVR	5	
155.38	GDNF	161	TKEELIFR	2	RETECD	544	DKGISKR	2	
147.34	RETECD	517	TDEPRFPACAEKR	12	GDNF	136	VKGQGR	2	*
140.5	RETECD	597	DLYGIKAGHGTCYCFEGK	6	GDNF	161	TKEELIFR	2	
127.49	GDNF	195	KLDKDTPSR	4	RETECD	511	FPACAEKR	7	
121.09	GFRa1	158	ENNCLNAAKACNLNDTCKK	9	GDNF	161	TKEELIFR	2	
118.13	GFRa1	83	LVLDALKQSPLYNCR	7	RETECD	105	SPKDLTLK	3	
118.11	GFRa1	149	LAPIYSGEPALAKENNCLNAAK	13	GDNF	161	TKEELIFR	2	
112.24	GDNF	225	TCCRPIAFDDDISFLDDSLEYHTLKK	25	GFRa1	191	RKCHK	2	
111.66	RETECD	120	VGVSSTPPMKVMCTILPTVEVK	10	GFRa1	83	LVLDALKQSPLYNCR	7	
99.02	GFRa1	48	ANELCLKEPGCSSKYR	14	GDNF	231	KCACV	1	#
92.69	GFRa1	241	RQTIVPACSYEDKERPNCLTLQVSCK	13	RETECD	340	GTVHEYKLK	7	
82.45	GFRa1	83	LVLDALKQSPLYNCR	7	RETECD	110	DLTLKVGVSSTPPMK	5	
79.74	GFRa1	34	LDCVKANELCLK	5	GDNF	136	VKGQGR	2	*

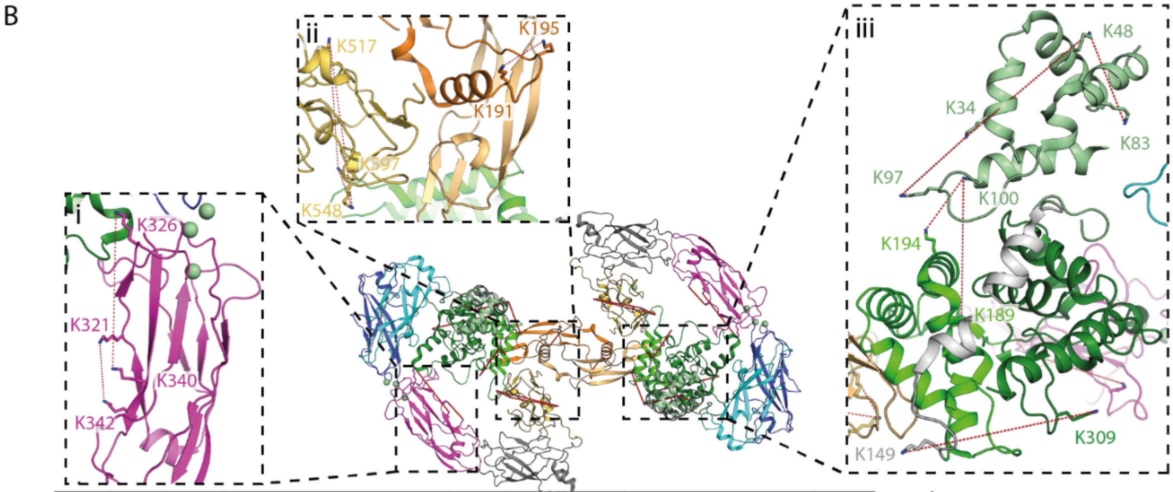

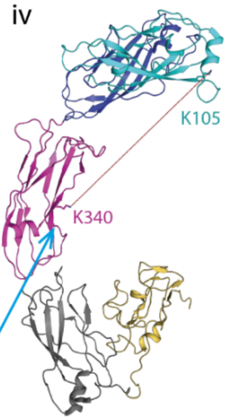

Score	Protein1	PepPos1	PepSeq1	LinkPos1	Protein2	PepPos2	PepSeq2	LinkPos2	
214.96	GDNF	195	KLDKDTPSR	4	GDNF	136	VKGQGR	2	*
171.33	GFRa1	189	VSTAEVCNKR	9	GFRa1	100	EKNCLR	2	
166.9	GFRa1	71	ESNFSMATGMEAKDECR	13	GFRa1	48	EPGCSSKYR	7	*
163.04	GDNF	161	TKEELIFR	2	GDNF	136	VKGQGR	2	*
159.98	GFRa1	309	SPKISVSPFCDCSSSGNSKEECDR	3	GFRa1	149	LAPIYSGEPALAKENNCLNAAK	13	
147.39	RETECD	326	FREEKAIFGNVR	5	RETECD	340	GTVHEYKLK	7	
144.03	GDNF	191	ILNNLTHNKK	9	GDNF	136	VKGQGR	2	*
133.6	RETECD	517	FPACAEKR	7	RETECD	548	DKGISKR	6	
119.14	GDNF	191	YCSGPCHDAETNYDKILNNLTHNKK	24	GDNF	195	LDKDTPSR	3	
118.75	RETECD	597	DLYGIKAGHGTCYCFEGK	6	RETECD	548	DKGISKR	6	
117.99	RETECD	342	LKLSQNLSVTEQR	2	RETECD	320	NNFAIEHKFR	8	
104	GFRa1	34	LDCVKANELCLK	5	GFRa1	97	RGMKK	4	
100.67	GFRa1	194	KCHKALR	4	GFRa1	100	EKNCLR	2	
99.02	GFRa1	47	ANELCLKEPGCSSKYR	14	GFRa1	83	LVLDALKQSPLYNCR	7	
98.46	RETECD	597	DLYGIKAGHGTCYCFEGK	6	RETECD	517	FPACAEKR	7	
88.56	GFRa1	71	ESNFSMATGMEAKDECR	13	GFRa1	83	LVLDALKQSPLYNCR	7	*
81.28	GFRa1	34	LDCVKANELCLK	5	GFRa1	47	EPGCSSKYR	7	
70.4	RETECD	340	GTVHEYKLK	7	RETECD	105	SPKDLTLK	3	

Supplementary Figure 6: Mapping interactions within the zRGα1a complex by XL-MS (Cross-Linking Mass Spectrometry). A) Intermolecular crosslinks identified between lysine side-chains from RET^{ECD}, $zGFR\alpha1a^{D1\text{-}D3}$ and $zGDNF^{mat.}$ highlighted in the C2 zRGα1a model, close-ups of the residues in the insets; RET^{CLD1}-$zGFR\alpha1a^{D1}$(i), RET^{CLD3}-$zGFR\alpha1a^{D3}$(ii) and RET^{CRD}-$zGFR\alpha1a^{D2}$-$zGDNF^{mat}$ (iii). B) Intramolecular crosslinks between lysine side-chains from RET^{ECD}, $zGFR\alpha1a^{D1\text{-}D3}$ and $zGDNF^{mat.}$ highlighted in the C2 zRGα1a model, close-ups of the residues in the insets; RET^{CLD3} (i), RET^{CRD}/ $zGDNF^{mat.}$ (ii) $zGFR\alpha1a^{D1\text{-}D3}$ (iii). (iv) Shows an intramolecular crosslink between CLD1 and CLD3 indicating flexibility at the calcium binding site. The crosslinked peptides highlighted with * do not have any structural model therefore are not shown in the model. The crosslinked peptide highlighted with # represents domain 1 of zGFRα1a cross-linked to zGDNF which has a distance of ~59 Å. This may indicate that $zGFR\alpha1a^{D1}$ is quite mobile consistent with the poorer quality of the map for this domain. The overall C2 zRGα1a model is represented as a cartoon with the crosslinks formed between lysines using disuccinimidyl sulfoxide (DSSO) represented as red lines between crosslinked lysines represented as sticks. The model is coloured according to its domains; CLD1 in cyan, CLD2 in blue, CLD3 in magenta, CLD4 in grey, CRD in yellow, $zGFR\alpha1a^{D1}$ in pale green, $zGFR\alpha1a^{D2}$ in green, $zGFR\alpha1a^{D3}$ in dark green and zGDNF in orange. All images were rendered in PyMOL (Schrodinger, 2015). Related to STAR methods.

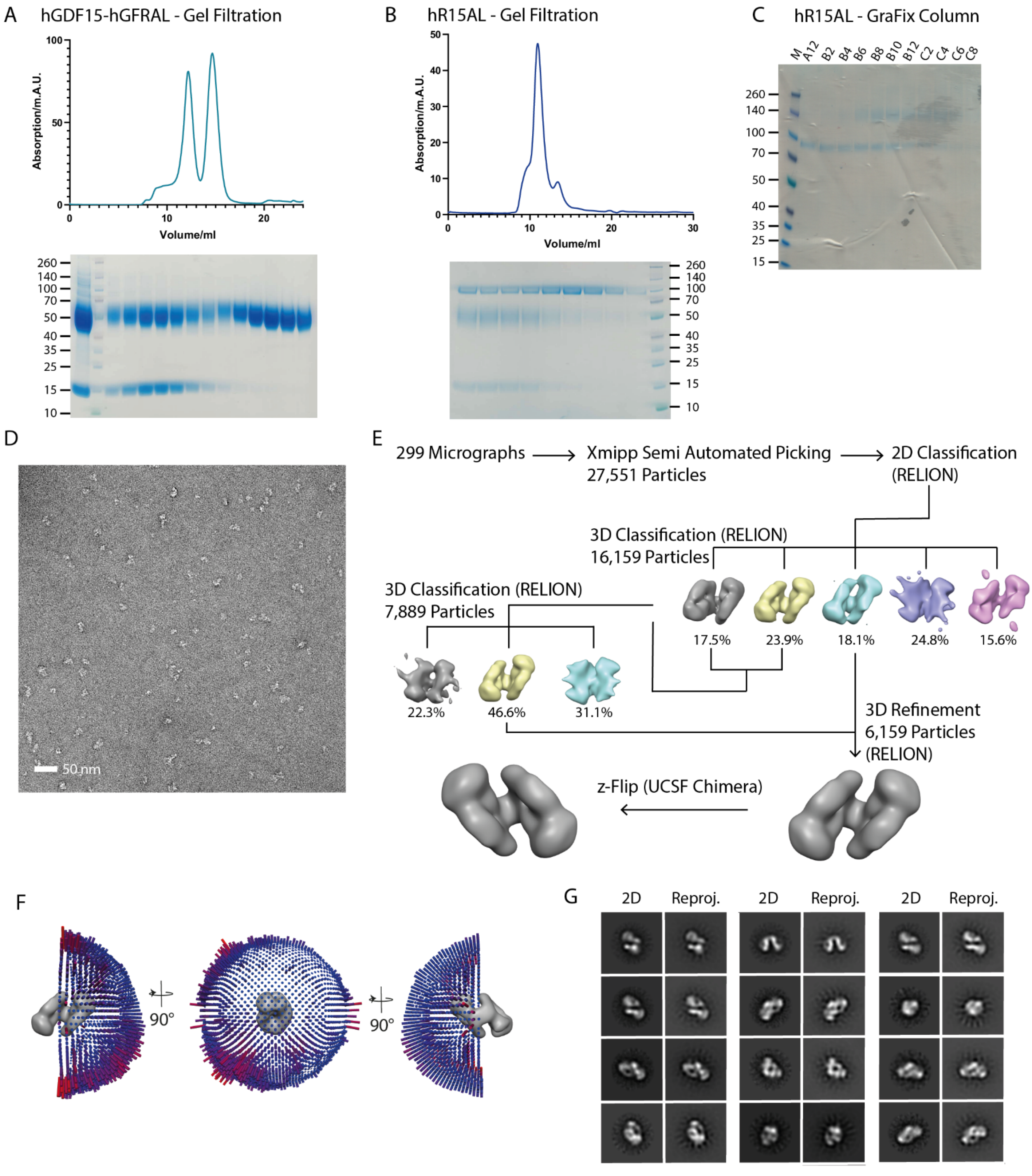

Supplementary Figure 7: hR15AL sample preparation and EM data processing. Size exclusion profiles and Coomassie-stained SDS-PAGE gels of A) hGDF15-hGFRAL and B) hRET-hGDF15-hGFRAL (hR15AL). C) fractions from the GraFix (Gradient Fixation) of hR15AL. D) A representative negative stain micrograph of the hR15AL-XL complex. E) The data processing pipeline leading to the final negative stain envelope of hR15AL. F) The particle distribution in the negative stain envelope with C2 symmetry applied. G) Projection matching, performed using Xmipp projection match (De la Rosa-Trevín et al., 2013), between the RELION (Kimanius et al., 2016; Scheres, 2012; Zivanov et al., 2018) showing 2D class averages from the particles that comprise the final reconstruction and different views of the 3D envelope. Related to Figure 6.

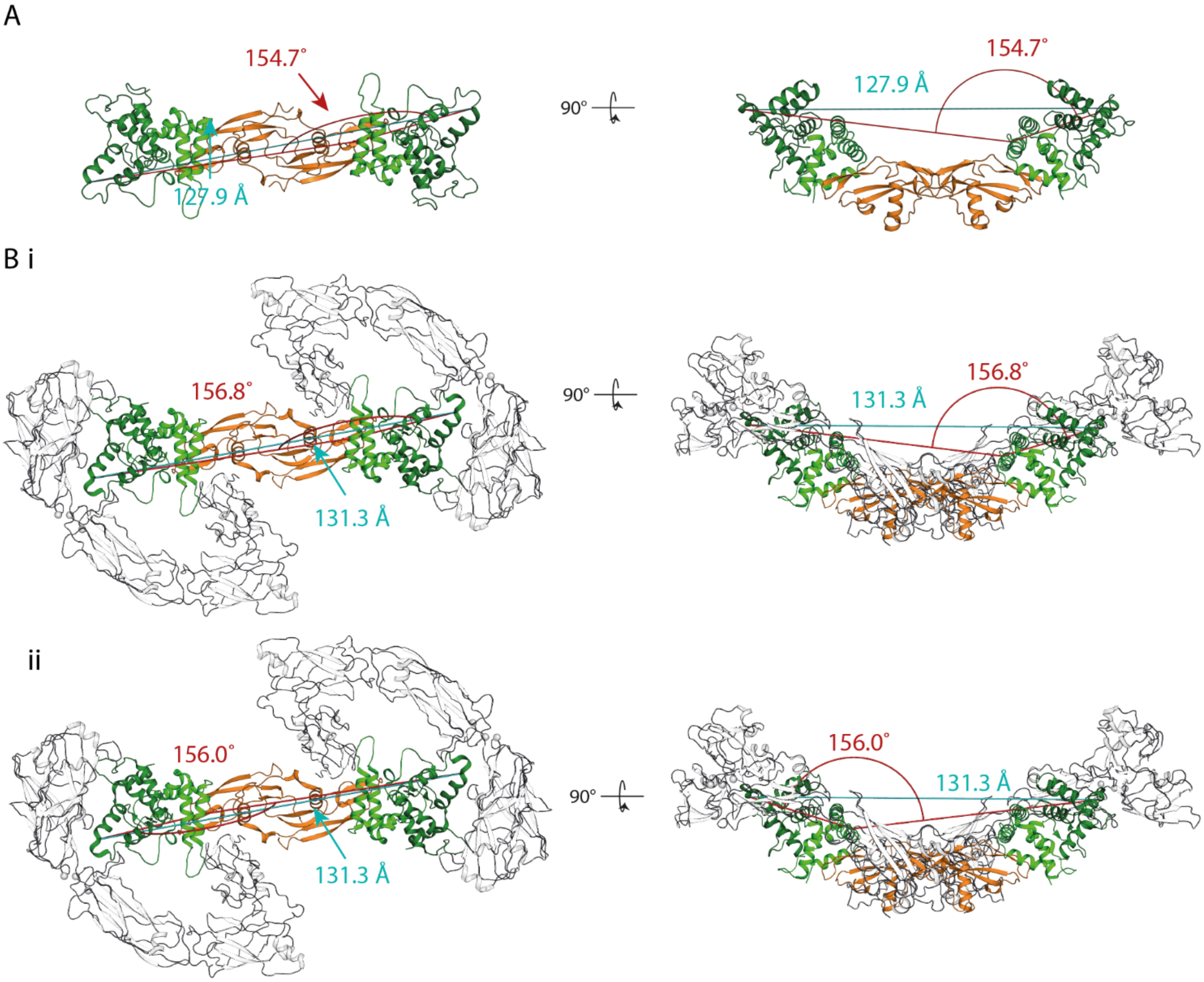

Supplementary Figure 8: Evidence for limited conformational flexing of zGDNF-zGFRα1a in the presence or absence of $zRET^{ECD}$. A) The crystal structure of a 2:2 zGDNF-zGFRα1a is shown as a cartoon, with GDNF in light orange, $zGFR\alpha 1a^{D2}$ in light green and $zGFR\alpha 1a^{D3}$ in green. The distance between K325 from symmetry-related molecules of zGFRα1a is highlighted in cyan and the angle between the K325-A172 from one molecule of zGFRα1 and K325 in the second molecule of zGFRα1a in red. B) 2:2 ligand:co-receptor zGDNF-$zGFR\alpha 1a^{D2\text{-}D3}$ built into the cryo-EM zRGα1a structure, represented as a cartoon, with $zGFR\alpha 1a^{D2}$, $zGFR\alpha 1a^{D3}$ and zGDNF in green, forest green and orange, respectively, and $zRET^{ECD}$ in grey. The distance between K325 from symmetry-related molecules of zGFRα1a highlighted in cyan and the angle between the K325-A172 from one molecule of zGFRα1 and K325 in the second molecule of zGFRα1a in red, there are two angles calculated using these residues in the C2 averaged structure highlighted in parts (i) and (ii) of the figure. All images were rendered in PyMOL (Schrodinger, 2015). Related to Figure 4.

SUPPLEMENTARY TABLES

	LIGAND				CO-RECEPTOR				RET	
	zGDNF	hGDNF	hNRTN	hGDF15	zGFRα1	hGFRα1	hGFRα2	hGFRAL	zRET	hRET
**LIGAND**										
zGDNF	1530Å				866Å				347Å	
zGDNF*									251Å	
hGDNF		1003Å				961Å				187Å
hGDNF*										148Å
hNRTN			2006Å				843Å			296Å
hNRTN*										218Å
hGDF15				1398Å				577Å		407Å
hGDF15*										369Å
**CO-RECEPTOR**										
zGFRα1									846Å	
hGFRα1										872Å
hGFRα2										960Å
hGFRAL										1094Å

Interface sizes (averaged over both protomers) calculated by PDBePISA

* Contact surface to the second protomer of GFL dimer

Supplementary Table 1: Major interface sizes for ternary complexes of hRET/zRET, GFRα1/α2/GFRAL and GDNF/NRTN/GDF15. Related to STAR methods.

SUPPLEMENTARY DOCUMENT S1

XL-MS analysis of the zRET^{ECD}-GFRα1-GDNF complex:

All chemicals were purchased from Sigma at the highest purity unless otherwise stated. A total of 200 ng protein in 20 mM HEPES (pH 7.5), 150 mM NaCl, 1 mM $CaCl_2$ was cross-linked using 1 mM disuccinimidyl sulfoxide (DSSO)(Kao et al., 2011) (Thermo Fisher Scientific) with mild shaking for 15 min at 37 °C. The reaction was quenched using a final concentration of 5% hydroxylamine for a further 15 min at 37 °C. The sample was subsequently alkylated, reduced and proteolysed. To do this, the sample was dried to completion using vacuum centrifugation and resolubilised with sonication into 8 M urea. Cysteine reduction was carried out using 2.5 mM TCEP for 30 min at 37 °C and alkylated in the dark using 5 mM iodoacetamide at room temperature for 30 min. The urea was diluted to 1 M using 50 mM triethylammonium bicarbonate and proteins were proteolysed using trypsin (Pierce) at 1:50 w/w trypsin:protein overnight at 37 °C. The solution was acidified to pH 2-3 using trifluoroacetic acid (TFA) and desalted using in house built STAGE tips made using Empore SPE C18 disks (3M, 66883-U). The eluent was then dried to completion.

Liquid Chromatography Mass Spectrometry

Peptides were reconstituted in 0.1 % TFA (v/v) and chromatographically resolved using an Ultimate 3000 RSLCnano (Dionex) HPLC. Peptides were first loaded onto an Acclaim PepMap 100 C18, 3 µm particle size, 100 Å pore size, 20 mm x 75 µm ID (Thermo Scientific, 164535) trap column using a loading buffer (2 % acetonitrile (MeCN) (v/v) and 0.05 % TFA in 97.95 % H2O) with a flow rate of 7 µL/min. Chromatographic separation was achieved using an EASY-Spray column, PepMap C18, 2 µm particles, 100 Å pore size, 500 mm x 75 µm ID (Thermo Scientific, ES803). The gradient utilised a flow of 0.275 µL/min, starting at 98 % mobile A (0.1% formic acid, 5 % dimethyl sulfoxide (DMSO) in H_2O) and 2 % mobile B (0.1 % formic acid, 75 % MeCN, 5% DMSO and 19.9 % H2O). After 3 min mobile B was increased to 8 % over 3 min, increased to 25 % over 69 min, to 45 % over 35 min, further increased to 90% in 17 min and held for 5 min. Finally, mobile B was reduced back to 5 % over 3 min for the rest of the acquisition.

MS1 data were acquired in real time over 150 minutes using an Orbitrap Fusion Lumos Tribrid mass spectrometer in positive, top speed mode with a cycle time of 5 s. The chromatogram (MS1) was captured using 60,000 resolution, a scan range of 375-1500 with a 50 ms maximum injection time, and 4e5 AGC target. MS2 dynamic exclusion with repeat count 2, exclusion duration of 30 s, 20 ppm tolerance window was used, along with isotope exclusion, a minimum intensity exclusion of 2e4, charge state inclusion of 3-8 ions and peptide mono isotopic precursor selection. Precursors within a 1.2 *m/z* isolation window were then fragmented using 25 % normalised collusion-induced dissociation (CID), 100 ms maximum injection time and 5 e4 AGC target. Scans were recorded using 30,000 resolution in centroid mode starting 120 *m/z*. MS3 spectra containing peaks with a mass difference of 31.9721 Da were further fragmented with a 43 % normalised higher collision induced dissociation, using a 2 *m/z*

isolation window, 150 ms maximum injection time and 2e4 AGC target. 4 scans were recorded using an ion trap detection in rapid mode starting at 120 *m/z*.

Data analysis.

Data processing was carried out using Proteome Discoverer Version 2.3 (ThermoFisher Scientific) with the XlinkX node(2017; Liu et al., 2015). The acquisition strategy was set to MS2_MS3 mode. The database comprised solely of the specific zRET^{ECD}, zGFRα1a^{D1-3} and GDNF$^{mat.}$ sequences. Trypsin was selected as the proteolytic enzyme allowing up to two missed cleavages with a minimal peptide length of five residues. Masses considered were in the range of 0.3-10 kDa. The precursor mass tolerance, FTMS fragment mass tolerance, and ITMS Fragment Mass Tolerance were set to 10 ppm, 20 ppm and 0.5 Da respectively. A static carbamidomethyl (+57.021 Da) modification was utilised for cysteine residues, with an additional dynamic modification for oxidation (+15.995 Da) on methionine residues. The False Discovery Rate (FDR) threshold was set to 0.05 with percolator as the strategy. The list of reported cross-linked spectral matches were manually examined and cross-links with spectra that did not contain acceptable b and y ion coverage were excluded. The reduced list was exported to crosslinkviewer.org (Combe et al., 2015) in order to graphically view the cross-links.
