# Supplementary material for: A two-site flexible clamp mechanism for RET-GDNF-GFRα1 assembly reveals both conformational adaptation and strict geometric spacing
Source: Structure. 2021 Jul 1;29(7):694–708.e7. doi: 10.1016/j.str.2020.12.012 (PMC8266384; doi:10.1016/j.str.2020.12.012)
Supplement: Document S2. Article plus supplemental information [file mmc2.pdf]

Structure

Article

A two-site flexible clamp mechanism for RET-GDNF-GFRα1 assembly reveals both conformational adaptation and strict geometric spacing

Graphical Abstract

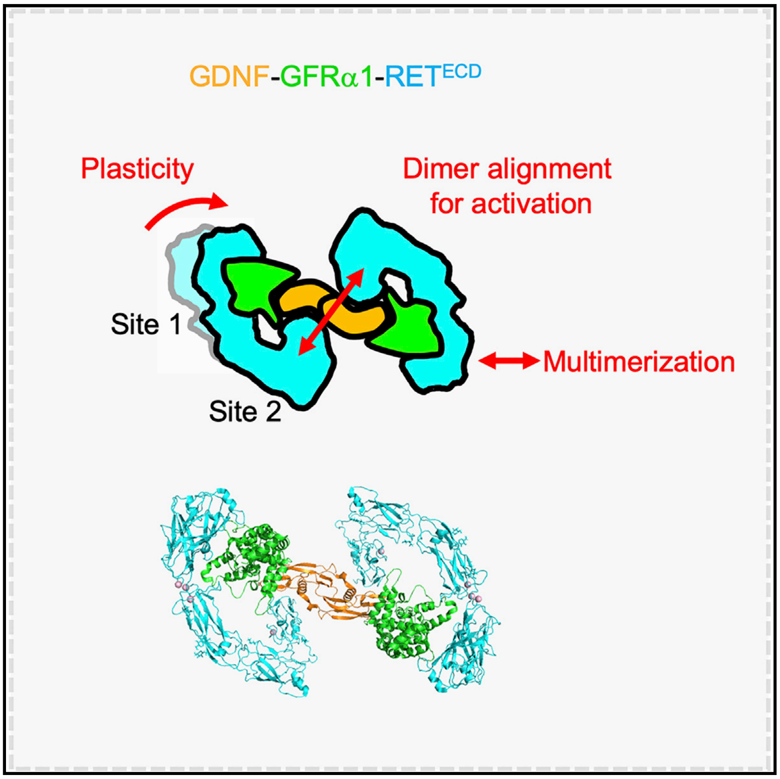

HIGHLIGHTS

- X-ray structure of zebrafish RET^{CLD1-4} module reveals conformational flexibility
- Conformational differences between RET^{CLD1-4} and a liganded RET^{ECD} cryo-EM structure
- Spatial separation of RET^{ECD} CRD dimer C termini imposed by each ligand dimer
- Differences in GDNF and GDF15 co-receptor engagement of RET and multimerization evidence

Authors

Sarah E. Adams, Andrew G. Purkiss, Phillip P. Knowles, ..., Francesca M. Houghton, Svend Kjær, Neil Q. McDonald

Correspondence

neil.mcdonald@crick.ac.uk

In Brief

Adams et al. use X-ray crystallography and cryo-electron microscopy to probe conformational changes in RET due to binding of GDNF ligand-GFRα1 co-receptor. The study shows a two-site clamping mechanism with flexible adaptations near the RET calcium sites but near identical spacings of the RET cysteine-rich domains established by GDNF/GDF15 ligand dimers.

Adams et al., 2021, Structure *29*, 694–708
July 1, 2021 © 2021 The Authors. Published by Elsevier Ltd.
https://doi.org/10.1016/j.str.2020.12.012

CellPress

CellPress
OPEN ACCESS

Structure

Article

A two-site flexible clamp mechanism for RET-GDNF-GFRα1 assembly reveals both conformational adaptation and strict geometric spacing

Sarah E. Adams,[1] Andrew G. Purkiss,[2] Phillip P. Knowles,[1] Andrea Nans,[2] David C. Briggs,[1] Annabel Borg,[2] Christopher P. Earl,[1] Kerry M. Goodman,[1,6] Agata Nawrotek,[1,7] Aaron J. Borg,[3] Pauline B. McIntosh,[4] Francesca M. Houghton,[1] Svend Kjær,[2] and Neil Q. McDonald[1,5,8,*]

[1]Signalling and Structural Biology Laboratory, Francis Crick Institute, NW1 1AT London, UK
[2]Structural Biology Science Technology Platform, Francis Crick Institute, NW1 1AT London, UK
[3]Mass Spectrometry Science Technology Platform, Francis Crick Institute, NW1 1AT London, UK
[4]Structural Biology of Cells and Viruses Laboratory, The Francis Crick Institute, 1 Midland Road, London NW1 1AT, UK
[5]Institute of Structural and Molecular Biology, Department of Biological Sciences, Birkbeck College, Malet Street, London WC1E 7HX, UK
[6]Present address: Zuckerman Institute, Columbia University, New York, NY, USA
[7]Present address: École normale supérieure Paris-Saclay, Gif-sur-Yvette, France
[8]Lead contact
*Correspondence: neil.mcdonald@crick.ac.uk
https://doi.org/10.1016/j.str.2020.12.012

SUMMARY

RET receptor tyrosine kinase plays vital developmental and neuroprotective roles in metazoans. GDNF family ligands (GFLs) when bound to cognate GFRα co-receptors recognize and activate RET stimulating its cytoplasmic kinase function. The principles for RET ligand-co-receptor recognition are incompletely understood. Here, we report a crystal structure of the cadherin-like module (CLD1-4) from zebrafish RET revealing interdomain flexibility between CLD2 and CLD3. Comparison with a cryo-electron microscopy structure of a ligand-engaged zebrafish RET^{ECD}-GDNF-GFRα1a complex indicates conformational changes within a clade-specific CLD3 loop adjacent to the co-receptor. Our observations indicate that RET is a molecular clamp with a flexible calcium-dependent arm that adapts to different GFRα co-receptors, while its rigid arm recognizes a GFL dimer to align both membrane-proximal cysteine-rich domains. We also visualize linear arrays of RET^{ECD}-GDNF-GFRα1a suggesting that a conserved contact stabilizes higher-order species. Our study reveals that ligand-co-receptor recognition by RET involves both receptor plasticity and strict spacing of receptor dimers by GFL ligands.

INTRODUCTION

Neurotrophic factors fulfill an essential function to support and protect both developing and mature neurons (Henderson et al., 1994). This neuroprotective therapeutic potential has led to an interest in understanding how they engage and activate their cell surface receptors (Airaksinen and Saarma, 2002; Allen et al., 2013). The glial cell line-derived neurotrophic factor (GDNF) family ligands (GFLs) constitutes an important family of neurotrophic factors that include GDNF (Durbec et al., 1996), Neurturin (NRTN) (Kotzbauer et al., 1996), Artemin (ARTN) (Baloh et al., 1998b), Persephin (PSPN) (Airaksinen and Saarma, 2002; Milbrandt et al., 1998), and more recently GDF15 (Emmerson et al., 2017; Hsu et al., 2017; Mullican et al., 2017; Yang et al., 2017). Each of these soluble factors are covalent dimeric ligands and are members of the cystine knot/transforming growth factor β (TGF-β) superfamily (Hinck et al., 2016). Each GFL has a cognate GFRα (GFR) co-receptor that associate as GDNF-GFRα1 (Cacalano et al., 1998), NRTN-GFRα2 (Baloh et al., 1997), ARTN-GFRα3 (Baloh et al., 1998a), PSPN-GFRα4 (Thompson et al., 1998), and GDF15-GFRAL (Emmerson et al., 2017; Hsu et al., 2017; Mullican et al., 2017; Yang et al., 2017) complexes, respectively. The GFL co-receptors typically consist of three related helical domains (D1 to D3) and are anchored at the membrane either through glycosylphosphatidylinositol linkages (GFRα1-4) or by a transmembrane helix (GFRAL). The bipartite GFL-GFR complexes are recognized by the RET receptor tyrosine kinase (RTK) forming ternary RET-GFL-GFR complexes (Cacalano et al., 1998; Durbec et al., 1996; Jing et al., 1996; Treanor et al., 1996). Engagement of GFL-GFR by RET triggers RET auto-phosphorylation of critical tyrosine residues to activate intracellular signaling pathways (Ibáñez, 2013; Mulligan, 2014).

RET has four consecutive cadherin-like domains (CLD(1-4)) and a membrane-proximal cysteine-rich domain (CRD) in its extracellular domain (RET^{ECD}) (Anders et al., 2001). The CLD domains diverge significantly, in sequence, structure, and arrangement from classical cadherins (calcium-dependent adhesion)

This is an open access article under the CC BY license (http://creativecommons.org/licenses/by/4.0/).

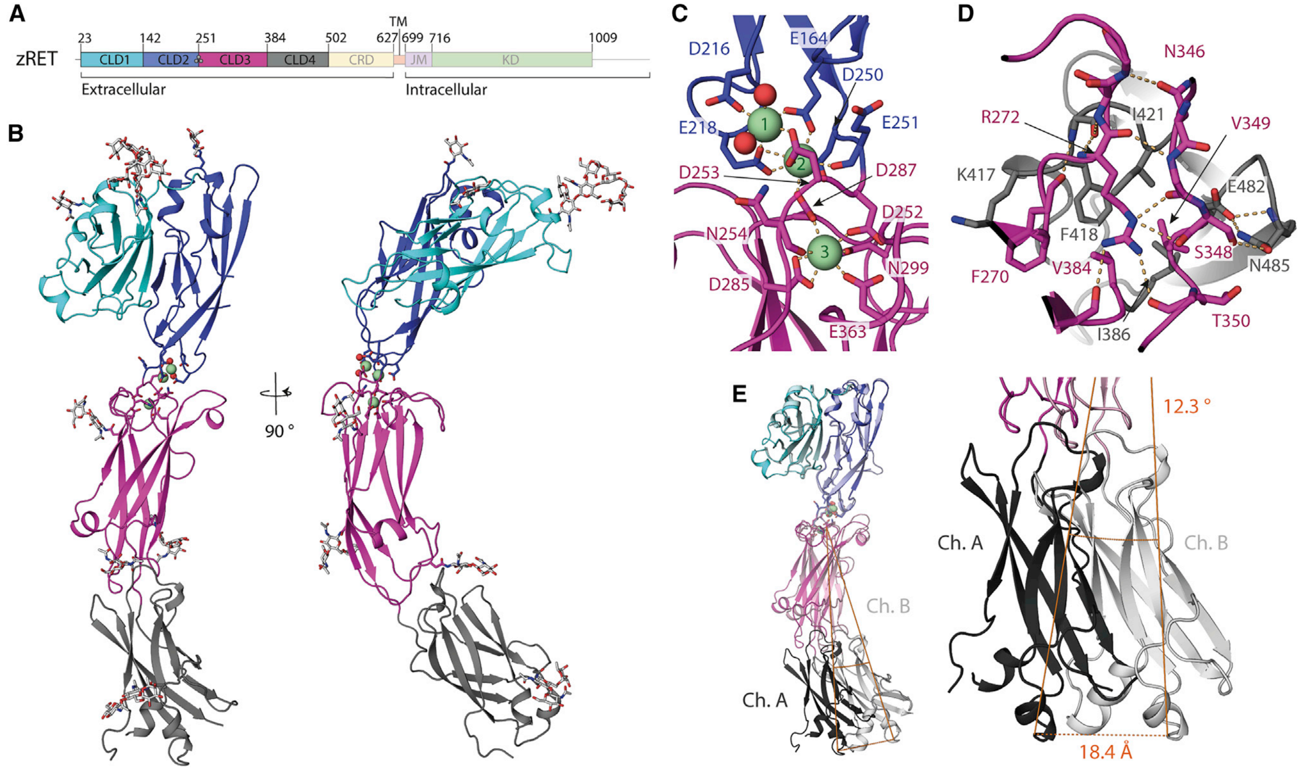

Figure 1. Crystal structure and flexibility of the zRET CLD(1-4) module

(A) Schematic of zebrafish RET receptor tyrosine kinase. CLD, cadherin-like domains; CRD, cysteine-rich domain; TM, transmembrane helix; JM, juxtamembrane domain; KD, kinase domain.

(B) Orthogonal views of zRET^{CLD1-4} colored as in (A). The calcium-binding site between CLD(2-3) has three calcium ions as green spheres with coordinating ligands as sticks and waters as red spheres.

(C) Close-up view of the coordination shell for the three calcium atoms between CLD2 and CLD3.

(D) Close-up of the interface between CLD3 and CLD4 centered on R272, selected side chains shown as sticks and dashed lines for hydrogen bonds.

(E) Superposition of chains A and B within the crystallographic asymmetric unit.

(Anders et al., 2001; Brasch et al., 2012; Kjær et al., 2010). For example, the CLD(1-2) pair form a closed clamshell arrangement (Kjær et al., 2010). Calcium ions are critical for RET folding consistent with the conservation of calcium-coordinating motifs between CLD2 and CLD3 (Anders et al., 2001; Kjær and Ibáñez, 2003; van Weering et al., 1998). Biochemical efforts to map the bipartite GDNF-GFRα1 binding site within RET^{ECD} to a minimal-binding domain have implicated the entire RET^{ECD} region. This contrasts many receptor-ligand interaction RTKs that frequently map to an ~200 amino acid minimal-binding domain (Lemmon and Schlessinger, 2010). Two key interactions between RET^{ECD}-GFRα1 and RET^{ECD}-GDNF were identified from electron microscopy structures of RET^{ECD} bound to GDNF/NRTN and GFRα1/GFRα2, although lacking a CRD structure (Bigalke et al., 2019; Goodman et al., 2014). A study by Li et al. (2019) revealed a human RET^{ECD} structure, including the CRD, in complex with several GFL ligands. In this analysis, the D1 domain of GDNF-GFRα1 or GDF15-GFRAL complexes with RET^{ECD} were missing. Moreover, little information about conformational changes upon ligand binding was evident.

We report an X-ray structure of zebrafish RET^{CLD-4} and a cryo-electron microscopy (cryo-EM) structure of the zebrafish RET^{ECD}-GDNF-GFRα1a complex. We observe plasticity within RET^{CLD1-4} and define the extent of conformational changes induced by ligand-co-receptor binding. Conformational adaptations are observed between RET and GFRα contacts even across clades, whereas a more strictly conserved interaction is observed between GFL and RET-CRD close to the transmembrane region. We show diversity in GFL co-receptor engagement by RET and describe RET^{ECD}-GDNF-GFRα1a multimers on cryo-EM grids generating linear arrays.

RESULTS

Crystal structure of zebrafish RET CLD(1-4) indicates localized flexibility

Crystals were obtained for a zebrafish RET construct spanning residues 22–504 (zRET$^{22-504}$) with glycosylation site mutations, N259Q, N308Q, N390Q, and N433Q (defined hereafter as zCLD(1-4)$^{red.sug.}$). Diffraction data from these crystals led to a structure determination at 2.2 Å resolution (Figure 1; Table 1). The final zCLD(1-4)$^{red.sug.}$ model contains residues 22–498 and includes 7 N-linked glycans well resolved in the electron density (Figure S1A). The crystals adopted the triclinic space group P1 and contained two molecules of CLD(1-4)$^{red.sug.}$ within the asymmetric unit. Each had a similar overall structure but with different

Table 1. Crystallography data processing and refinement statistics

	zCLD(1-4)$^{red.sug.}$	zGDNF$^{mat.}$-GFRα1a$^{\Delta D1}$
Wavelength (Å)	0.9787	0.9795
Resolution range (Å) [a]	65.96–2.20 (2.28–2.20)	50.76–2.2 (2.28–2.2)
Space group	P 1	P 21 21 2
Unit cell dimensions		
a, b, c (Å)	51.17, 70.50, 105.44	125.07, 55.54, 70.96
α, β, γ (°)	105, 101, 100	90, 90, 90
Total no. of reflections	229,073 (22,789)	51,646 (5,054)
Unique reflections	67,550 (4,539)	25,823 (1,368)
Multiplicity	3.4 (3.4)	2.0 (2.0)
Completeness (%)	91.28 (66.36)	91.93 (53.94)
Mean I/σI	7.09 (1.92)	14.30 (3.15)
Wilson B factor (Å^2)	28.88	20.87
R_{merge}	0.073 (0.68)	0.056 (0.37)
R_{meas}	0.087 (0.81)	0.079 (0.53)
R_{pim}	0.046 (0.43)	0.056 (0.37)
CC½	0.996 (0.70)	0.997 (0.80)
CC	0.999 (0.91)	0.999 (0.94)
Resolution used for refinement	65.96–2.20	50.76–2.20
Reflections used in refinement	62,771 (4,522)	23,743 (1,363)
Reflections used for R_{free}	3,098 (255)	1,152 (57)
R_{work}	0.232 (0.316)	0.199 (0.247)
R_{free}	0.277 (0.383)	0.230 (0.248)
CC (work)	0.895 (0.615)	0.888 (0.723)
CC (free)	0.884 (0.553)	0.888 (0.800)
No. of non-hydrogen atoms	7,997	2,736
Macromolecules	7,289	2,434
Ligands	539	83
Solvent	169	219
Protein residues	980	309
RMSD		
Bond lengths (Å)	0.009	0.006
Bond angles (°)	1.08	0.74
Ramachandran plot (%)		
Favored	96.6	97.03
Allowed	3.0	2.97
Outliers	0	0.0
Rotamer outliers (%)	0	0.0
Clashscore	14.56	4.03
Average B factor (Å^2)	41.31	30.19
Macromolecules	39.53	28.99
Ligands	67.62	55.09
Solvent	33.82	34.06
No. of TLS groups	8	1
PDB:	7AMK	7AB8

RMSD, root-mean-square deviation.
[a]Values in parentheses are for highest-resolution shell.

hinge angles between CLD2 and CLD3, pointing to flexibility within RET (Figure 1E).

The overall structure of zCLD(1-4)$^{red.sug}$ showed that all CLDs have the predicted canonical seven β strand sandwich architecture of cadherin domains (Figure S1B) (Shapiro and Weis, 2009). The amino-terminal CLD1 is packed against CLD2 in a fold-over clamshell arrangement as anticipated from human RET, while CLD(2-4) forms a “C-shape” (Figure 1B). The zCLD(1-2) clamshell has a surprisingly large overall root-mean-square deviation (RMSD) of 18.9 Å over 229 Cαs when superposed with hCLD(1-2) (Winn et al., 2011). Key features contributing to this structural divergence are a shuffled disulfide connectivity, a lack of a β hairpin and a longer CLD1 helix α1 between higher and lower vertebrates (Figure S1C) (Kjær et al., 2010).

The irregular CLD2-β1 (residues 153–160) is largely separated from the main CLD2 sheet and lies between CLD1-β1 and CLD2-β7, anchored largely through CLD2-β2 side chains (such as R172 and R176) rather than main-chain interactions (Figure S1D). One end of CLD2-β1 is tethered through packing of two short α helices from CLD2-β1 and CLD2-β2, while the other end is anchored by aromatic side chains from residues amino-terminal to CLD1-β1. This configuration contributes to a substantial internal cavity between CLD1 and CLD2, with a surface volume of ~510 Å^3 (Figure S1D). We note that analysis of the published human CLD(1-2) (Kjær et al., 2010) (PDB: 2X2U) also revealed a similar but smaller internal cavity of ~324 Å^3 (Figure S1D) (Abagyan et al., 1994; An et al., 2005; Fernandez-Recio et al., 2005). On the opposing side of the clamshell interface, CLD1-β2 and CLD2-β2 contribute through both side- and main-chain interactions.

The limited size of the CLD(2-3) interface is typical of a calcium-dependent cadherin domain pair, with three calcium ions (Ca-1/Ca-2/Ca-3) and their coordinating ligands dominating the interface (Figure 1C) (Shapiro and Weis, 2009). Ca-1 and Ca-2 lie in close proximity (3.9 Å apart in chain A) and share three coordinating ligands, the side chains of E164, E218 (CLD2), and D253 (CLD3). Ca-1 is exposed to the solvent at the edge of CLD2, with the coordination sphere completed with D216 and two water molecules, one of which is coordinated by with N165 (Figure 1C). The Ca-2 coordination sphere includes D253, a main-chain carbonyl from E251 (CLD2), and D287 (CLD3), which is a ligand shared with Ca-3 (Figure 1C). Ca-3 is buried within CLD3 and located 6.9 Å away from Ca-2, the coordination shell is completed with the side chains of D252, D285, N299, and D363 and the main-chain carbonyl of N254 (Figure 1C).

CLD3 consists of 135 amino acids and is the largest RET CLD. It shows the greatest structural divergence of all CLDs (~5 Å RMSD) compared with the smaller canonical cadherin domains (Figure S1B) (Shapiro and Weis, 2009). Additional elements

within CLD3 include a loop insertion between $\beta2$ and $\beta3$ adjacent to the calcium-binding site, an α helix between $\beta3$ and $\beta4$, and a much longer pair of antiparallel β strands, $\beta4$ and $\beta5$. Unusually, CLD3 lacks any disulfide bonds and its CLD4 interface is offset at one side of the domain giving a pronounced curvature to the entire CLD(1-4) module. CLD3 has five potential glycosylation sites, two of which were removed by site-directed mutagenesis in zCLD(1-4)$^{red.sug}$ and three are visible in the electron density (Figure S1A). These features collectively ensure that CLD3 plays a crucial role in the stability and curvature of the zCLD(1-4) module.

The CLD(3-4) interface diverges substantially from classical cadherins and has previously confounded efforts to predict the precise CLD(3-4) domain boundaries (Anders et al., 2001). It lacks calcium ions and has a predominantly hydrophobic character, with peripheral hydrophilic interface contacts (Figure 1D). Hydrophobic contacts include CLD3 side chains F270 and V349 that pack against CLD4 F418 and I421 side chains and are tethered by V384 from a rigid connecting linker with sequence P383-V384-P385. An exception to the hydrophobic character of the interface is the buried R272 side chain from the CLD3-$\beta1$-$\beta2$ loop (Figure 1D). The aliphatic portion of R272 packs against V349, V384, and I421, while its guanidinium head engages main-chain carbonyls on the CLD3-$\beta5$-$\beta6$ loop and the CLD3-CLD4 linker (Figure 1D). This residue is equivalent to R287 in humans, a known site of mutation in a severe form of Hirschsprung's disease (R287Q), highlighting the crucial nature of this residue for folding (Attie et al., 1995; Pelet et al., 1998).

Differences in the CLD interface size indicate flexibility between CLD2 and CLD3 but rigidity between CLD3 and CLD4. This is supported by superpositions of the two independent molecules of zCLD(1-4)$^{red.sug}$ demonstrating plasticity in the tapered CLD(2-3) interface (Figure 1E). Superimposing chain B onto chain A, aligned through CLD(1-2), reveals that the rigid CLD(3-4) module pivots about the CLD(2-3) calcium-binding site interface with a variation of 12.3°, which leads to a difference of 18.4 Å at the furthest point from the CLD(2-3) interface (Figure 1E). Subtle angular differences proximal to the calcium ions, propagating down the module lead to a tightening of the C-shaped structure between chain A and chain B.

Cryo-EM structure of the ternary zebrafish GDNF-GFRα1a-RET^{ECD} complex

A reconstituted complex was assembled consisting of the zRET^{ECD} (residues 1–627), a C-terminal truncated zGFRα1a (zGFRα1a^{D1-3}) covering residues 1–353, and an N-terminal truncated zGDNF, residues 135–235 (zGDNF$^{mat.}$), defined hereafter as zRGα1a from RET-GDNF-GFRα1a (Figures 2A and S2). The zRGα1a complex homogeneity and stability was improved by crosslinking using the GraFix technique (Kastner et al., 2008). An initial cryo-EM dataset (dataset 1) collected on the reconstituted zRGα1a yielded a 3D cryo-EM map that confirmed a 2:2:2 stoichiometry (see Figure S3), consistent with size-exclusion multi-angle laser light scattering data (Figure S2) and similar to recently published human RET complexes (Bigalke et al., 2019; Li et al., 2019). The map displayed substantial anisotropic resolution due to particle orientation bias on the grids. To overcome this, a second dataset was collected with a sample grid tilted at an angle of 30° (dataset 2) (see Figure S3). The combined particles from both datasets were used to generate an initial 3D volume with C2 symmetry applied in CryoSPARC-2 (Punjani et al., 2017). Additional processing with symmetry expansion in RELION-3 (Kimanius et al., 2016; Scheres, 2012; Zivanov et al., 2018), improved the anisotropy and resolution of the map by addressing flexibility at the 2-fold symmetry axis, to produce a map with a nominal resolution of 3.5 Å (Figures 2C, S4, S5A, and S5B). Subsequent analysis of this final map with 3DFSC indicated that there were a limited number of particles contributing to the Z direction of the 3D reconstruction, which resulted in the resolution in that direction being limited to ~10 Å (Figure S4) (Tan et al., 2017).

The zRGα1a cryo-EM map resembles a figure-of-eight with a molecular 2-fold centered at the crossover point (Figure 2B). To enable building of a full structure into the map, we determined a crystal structure of zGDNF^{mat}-zGFRα1a$^{151-353}$ lacking domain D1 (referred to hereafter as zGFRα1a$^{\Delta D1}$) at 2.2 Å (see the STAR Methods and Figure S5C). We then fitted crystal structures for zRET CLD(1-4) and zGDNF^{mat}-zGFRα1a$^{\Delta D1}$ into the symmetry-expanded map (Figure S5C) together with homology models for the zRET^{CRD} and zGFRα1a^{D1}. An initial model for zRET^{CRD} was generated from the hRET^{ECD}-hGFRα2-NRTN structure (Li et al., 2019) and for zGFRα1a^{D1} from the hGFRα2-NRTN (Sandmark et al., 2018) structure by substituting zebrafish sequences followed by model optimization using Swiss-Model (Schwede et al., 2003) and Modeller (Webb and Sali, 2016), respectively. The initial structure was refined against the symmetry-expanded map and rebuilt, before placing it into the C2-averaged map for further refinement in PHENIX (Adams et al., 2010) (Table 2; Figures S5A and S5B). The final near complete structural model has a crosscorrelation of 0.63 against this map with highest-resolution features close to GDNF and zRET$^{CLD4-CRD}$ (Figure S5). N-Acetylglucosamine (GlcNAcβ1-Asn) glycan rings linked to asparagine sites were also evident in the map. Density was also evident for zGFRα1a^{D1}, sandwiched between zGFRα1a^{D3} and zRET^{CLD1}, at a similar position to GFRα2^{D1} (Bigalke et al., 2019; Li et al., 2019; Sandmark et al., 2018) (Figure 2D).

The final structure shows zGDNF at the core of the complex flanked by two zGFRα1a^{D1-3} co-receptors, both of which are further enveloped by two "G"-shaped RET^{ECD} molecules (Figure 2D). The spur of the RET^{ECD} G shape is formed by the CRD domain making contacts with both GDNF protomers and zGFRα1a, as first predicted from lower-resolution negative stain EM analysis (Goodman et al., 2014) as well as other structures (Bigalke et al., 2019; Li et al., 2019). There are two major interfaces between zRET^{ECD} and its ligand-co-receptor at opposite ends of zRET^{ECD}, each is well defined in the cryo-EM map with side-chain level information (Figure 2D). The dominant interaction is between zCLD(1-3) and GFRα1^{D3} (defined hereafter as the site 1), with a key second site between zCRD and a concave surface presented by the GDNF dimer and a loop from GFRα1 (defined hereafter as site 2) (Figure 2D). Site 2 shows a close equivalence to the "low" affinity TGF-β receptor I binding site for TGF-β (Groppe et al., 2008; Kirsch et al., 2000) and is also used by other TGF-β superfamily ligands (Hinck et al., 2016).

Site 1 on zRET involves elements from the CLD(1-2) clamshell structure and the CLD(2-3) calcium-binding region (Figure 2D). Both contacts engage the zGFRα1 domain D3 (zGFRα1^{D3}) close to helix α4, its preceding loop and helix α1 . Together these

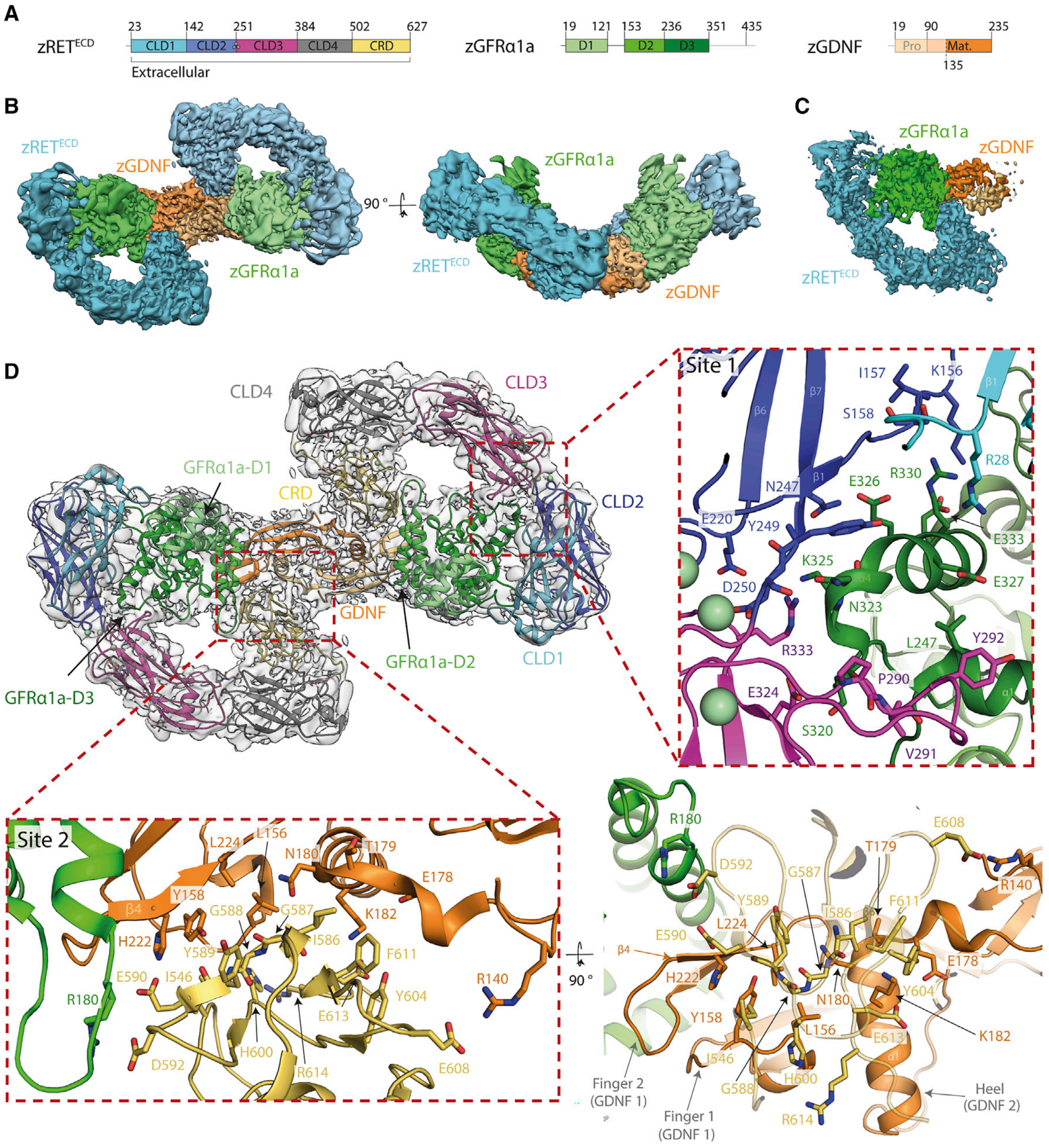

Figure 2. Cryo-EM structure of the zRET^{ECD}-zGFRα1a^{D1-3}-zGDNF$^{mat.}$ (zRGα1a) complex

(A) Schematic of zRET^{ECD}, zGFRα1a^{D1-3}, and zGDNF$^{mat.}$, color coded according to Figure 1A.

(B) Orthogonal views of the reconstituted zRGα1a complex cryo-EM map, projecting down the approximate molecular dyad or perpendicular to it. The cryo-EM map is segmented and colored by protein, with zRET^{ECD} cyan, zGFRα1a^{D1-3} green, and zGDNF$^{mat.}$ orange.

(C) Symmetry-expanded map of zRGα1a half-complex, with the map segmented and colored by protein as in (B).

(D) The final model of the zRGα1a complex built into the C2 symmetry map, colored light gray. The domains are colored as in Figure 1 with zRET^{ECD}: for GFRα1a domains D1-3 are pale green, green, and dark green, respectively; the two molecules of zGDNF$^{mat.}$ are orange and pale orange. Two sites of interaction within the zRGα1a complex are highlighted in red dashed boxes, labeled as site 1 (zGFRα1a-zRET) and site 2 (zGDNF-zRET). Interaction residues are highlighted as sticks and the backbone represented as a cartoon.

zGFRα1 elements form a wedge-shaped surface to access the calcium-binding region of zRET$^{CLD(2-3)}$. This interface covers a total area of 846 Å^2 and comprises both hydrophilic and electrostatic interactions, as calculated by PDBePISA (Krissinel and Henrick, 2007). The isolated CLD2-β1 strand bridges between the CLD1-CLD2 interface, running antiparallel to the zGFRα1^{D3} helix α4. Hydrophilic side chains from helix α4 interact with CLD2-β1 main chain as well as two proximal strands; CLD1-β1 and CLD1-β7 (Figure 2D). The side chain from R330 of zGFRα1^{D3} helix α4, lies close to the main-chain carbonyl of I157 from CLD2-

Table 2. EM data acquisition and processing statistics

	zRGα1a C2 map	zRGα1a symmetry-expanded map	zR15AL negative stain map
EMDB:	EMD-11822	EMD-11822	EMD-11777
PDB:	7AML		
Magnification	46,296	46,296	40,719
Voltage (kV)	300	300	120
Electron exposure (e^{-}/Å^2)	48.6	48.6	–
Defocus range (μm)	1.4–3.5	1.4–3.5	1.0–1.5
Pixel size (Å)	1.08	1.08	3.44
Symmetry imposed	C2	C1	C2
Initial particle images	2,424,600 (dataset 1), 1,393,023 (dataset 2)	–	27,551
Final particle images	382,547 (360,189 dataset 1 and 22,358 dataset 2)	765,094	6,519
Map resolution (Å)	3.3	3.5	26
FSC threshold	0.143	0.143	0.143
Map resolution range (Å)	12–3.3	11–3.5	
Refinement			
Initial model, PDB:			7AMK, 7AB8
Model resolution (Å)			4.2
FSC threshold			0.5
Map sharpening B factor (Å^2)			−75
Non-hydrogen atoms			16,020
Protein residues			1,996
Ligands			8
N-Glycans			16
Protein			122.4
Ligands			111.6
Bond lengths (Å)			0.004 (0)
Bond angles (°)			0.646 (6)
Validation			
MolProbity score			1.85
Clashscore			9.45
Poor rotamer (%)			0.89
Favored			94.94
Allowed			5.06
Disallowed			0.0

RMSD, root-mean-square deviation.

β1 and the side chain of E326 is positioned near the side chains of N247 and Y249 (hydroxyl). The loop preceding helix α4 of zGFRα1a^{D3} is anchored between the CLD3-β2-β3 loop and the CLD3-β4-β5 loop; main chain-main chain interactions form between P290 from the CLD3-β2-β3 loop and S321 of zGFRα1a^{D1-3} (Figure 2D). The main chain of N323 from the loop preceding α4 of zGFRα1a^{D1-3} appears to interact with the guanidinium head of R333 from CLD3-β4, and the side chain of N323 interacts with the main chain of D250 at the calcium-binding site (Figure 2D).

Site 2 interaction involves the zRET^{CRD} and a concave "saddle"-shaped surface formed by both protomers of the zGDNF$^{mat.}$ dimer and a loop from zGFRα1a^{D2} (Figure 2D). This agrees with our previous assignment of this site as a "shared" site (Goodman et al., 2014). The interface is mainly hydrophobic in character and has a surface area of 598 Å^2. The surface contains three main elements; a β-turn from zGFRα1^{D2} centered on R180, residues 156–159 (LGYR) and residues 222–224 (HTL) from the fingers of one GDNF protomer (GDNF1) and residues 176–179 (DATN) with the "heel" helix of the second protomer (GDNF2). These residues engage G588 and Y589 from the CRD-β3-β4 loop (Figure 2D) and make van der Waals's contacts to the I546 side chain from the CRD-β1-β2 loop (Figure 2D). A hydrophobic interaction is evident between I586 from the CRD-β3-β4 loop and the T179 from the loop preceding the zGDNF2 heel (Figure 2D). The remaining contacts are mainly hydrophilic in nature between the heel of GDNF2 and the CRD. From the heel of zGDNF2; N180^{GDNF} interfaces with the amide of G587, and K182 of GDNF2 interacts with E613. This contact is consistent with the absence of a crosslink in the XL-MS data (Figure S6). The zRET^{CRD} β5-β6 β turn is 2 amino acids shorter than hRET^{CRD} allowing it to engage amino-terminal residues 138–140 of zGDNF2 with a likely salt bridge between E607 and R140. Also, H222 from zGDNF1 is likely to contact E590 (equivalent to E595 in human RET, a known Hirschprung's mutation site) (So et al., 2011).

Two further contacts with zRET are indicated but are less well defined in the map. A limited interface between zRET^{CLD1} and GFRα1^{D1} is observed allowing zRET^{CLD1} and GFRα1^{D1} domains to be placed and the interaction is very similar to that seen in the RET^{ECD}-NRTN-GFRα2 structure (Li et al., 2019). Second, residues immediately after the CRD from residues 615 to 627 are poorly ordered. This acidic stretch includes 12 residues likely to pass beneath the highly basic GDNF ligand (pI of 9.3 for mature zGDNF) before entering the plasma membrane. The final residue in RET^{ECD} observed is P617, which is separated by a distance of 40.9 Å from the dimer equivalent residue. A lower map contour shows density for these residues beneath the GDNF molecular 2-fold axis consistent with RET^{ECD}-NRTN-GFRα2 (Bigalke et al., 2019).

Clade-specific features influence ligand binding affinity

Comparison of site 1 of zRET in both the crystal and cryo-EM structure reveals differences in the conformation of residues 288–298 from a CLD3 loop (Figure 3A). In the absence of ligand, this loop packs against CLD3 core (loop "down" position) interacting with the β4 strand. In the presence of the ligand, this loop forms a central part of the interface with zGFRα1a^{D3} and is repositioned upward (loop-"up") toward the calcium ions and engages L247 of helix α1 of zGFRα1a^{D2} (Figure 3A). No equivalent interaction is observed for the human RET CLD3 structure (Figure 3B). The cryo-EM map clearly shows zGFRα1a^{D3} side-chain contacts with Y292 and how this residue shifts substantially

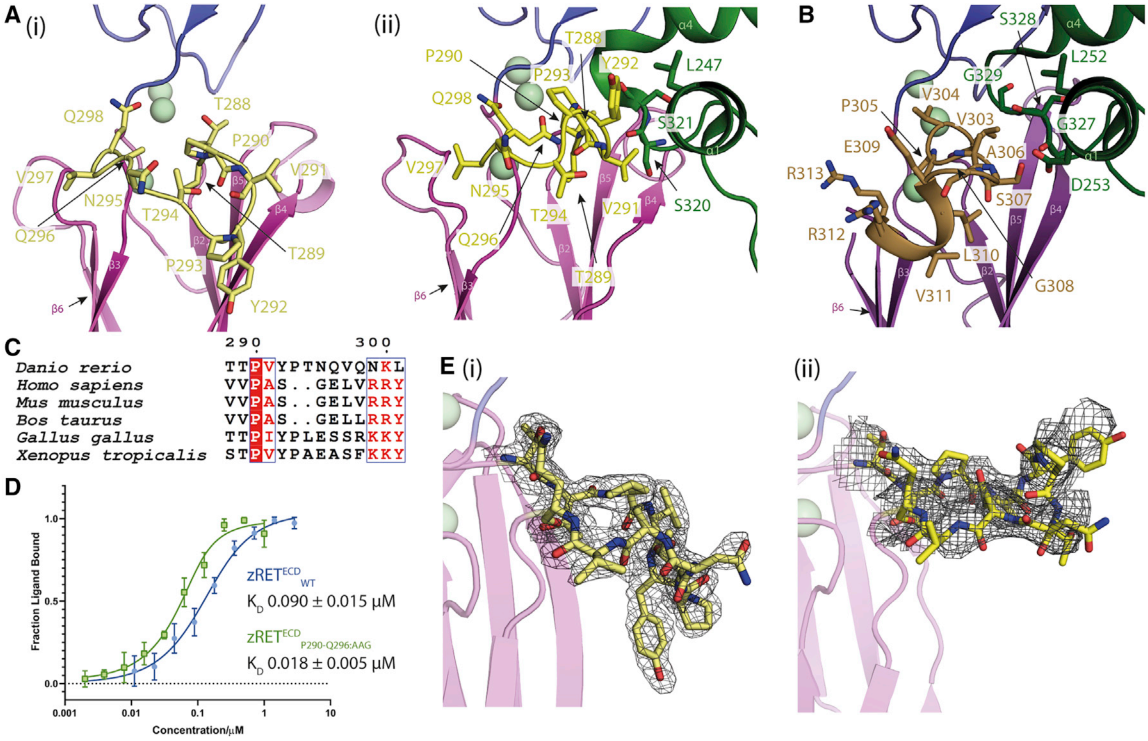

Figure 3. Ligand-co-receptor-induced conformational changes in $zRET^{ECD}$

(A) The CLD3-β2-β3 loop is shown in yellow as sticks (i) projects "downward" in the view shown for zRET CLD(1-4) (see the orientation of Y292 side chain) and (ii) projects "upward" to engage the $GFR\alpha1^{D2}$ α1 helix (green sticks) in the zRGα1a structure.

(B) The shorter CLD3-β2-β3 loop and extra helix from the human RET^{ECD}-NRTN-GFRα2 structure (PDB: 6Q2O) shown as olive colored sticks, domains colored as in Figure 1.

(C) Sequence alignment of RET CLD3-β2-β3 loop sequences by Espript (http://espript.ibcp.fr) (Robert and Gouet, 2014).

(D) Binding curves and calculated K_D values for $zRET^{ECD}_{wt}$ and mutant ($zRET^{ECD}_{P291-Q296:AAG}$) binding to $zGFR\alpha1a_2$-$zGDNF_2$ measured by MST, with a minimum of n = 3 repeats for the WT and the mutations with the SE represented.

(E) (i) Electron density map calculated using m2Fo-DFc coefficients over the CLD3-β2-β3 loop, yellow sticks and contoured at 1.0σ. (ii) Coulombic potential cryo-EM map for CLD3-β2-β3 loop from the zRGα1a complex (black mesh). Calcium ions are represented as pale green spheres.

relative its unliganded position (Figure 3C). This movement of 18.5 Å (hydroxyl-hydroxyl) or 8.3 Å (Cα-Cα) also results in main chain amides from P290 and V291 of the CLD3-β2-β3 loop lying close to the main-chain carbonyl of S320 from $zGFR\alpha1a^{D3}$, forming a pseudo-β sheet interaction (Figure 3A).

In view of the critical role of this loop in the zRET co-receptor recognition, it is surprising that loop CLD3-β2-β3 contains an "indel" of two extra amino acids Y292 and P293, unique to lower vertebrates (Figure 3D). The equivalent shorter loop in human RET adopts a helical turn connecting the two β strands (Figure 3C) (Li et al., 2019). To probe the contribution of the CLD3-β2-β3 loop to zGDNF-zGFRα1a binding, we truncated the residues P290-Q296 to AAG and assessed its ligand binding properties by microscale thermophoresis (MST). Surprisingly loop truncation improved binding affinity for the ligand-co-receptor by 5-fold compared with wild-type (WT) $zRET^{ECD}$, with a dissociation constant (K_D) of 18 nM (±5 nM) compared with 90 nM (±15 nM) for WT zRET (Figure 3E). This increase in affinity implies either that higher vertebrates RET^{ECD} have a higher affinity for ligand than their lower vertebrate counterparts or that the loop contributes to an auto-inhibitory function in lower vertebrates. Taken together, our structural results show an unexpected conformational change in a clade-specific loop proximal to the CLD(2-3) calcium sites.

Comparisons of interfaces within ternary RET complexes either between species (human and zebrafish GDNF-GFRα1) or paralogs (Neurturin-GFRα2 and GDF15-GFRAL) reveal considerable variation in contacts at site 1 and nearly identical contacts at site 2. This translates into a substantial variation in the size of these interfaces (Table S1).

One contributing factor to these variations is the additional contacts seen between helix α1 of $zGFR\alpha1^{D3}$ and residues 288–298 of zRET. Another example is GFRAL, which makes multiple additional contacts through residues 247–266, centered on the disulfide C252-C258. These contacts engage residues flanking the β hairpin at Y76/R77 and R144/Y146 on the CLD1 β7 strand. Both elements are unique to higher-vertebrate RET and contribute to the ligand-free RET dimer interface (Kjær et al., 2010; Li et al., 2019).

Comparison of all available liganded RET^{ECD} structures at site 2 consistently show a spacing of 44.2–47.0 Å between each pair of CRD C termini (measured at residue E613/620 in zRET/hRET) within an RET dimer (Figures 4A–4C). This suggests a stringent requirement for CRD spacing to couple the transmembrane and intracellular modules. We note this distance is defined by

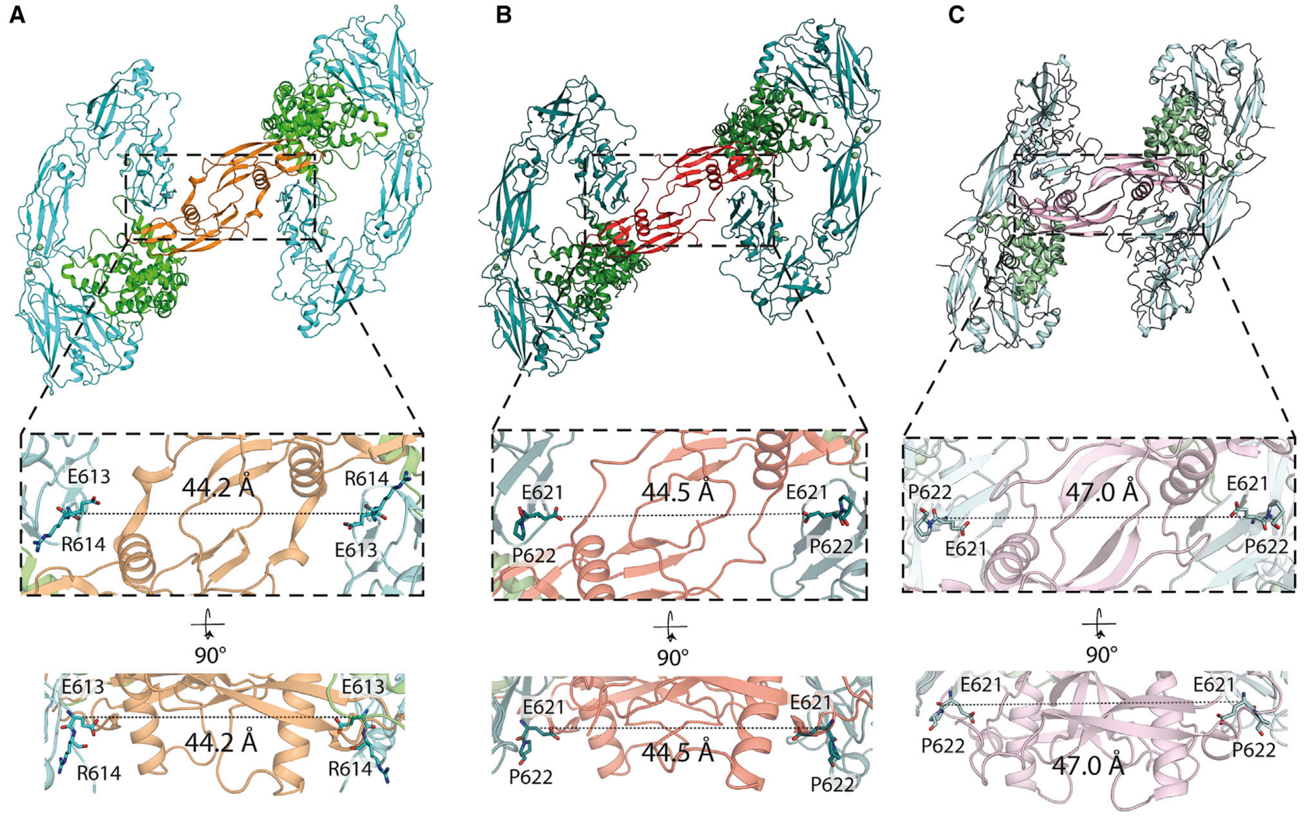

Figure 4. Different GFL ligands establish a conserved spacing between RET CRD-CRD pairs in their respective ternary complexes
(A) Separation between the Cα of E613 (equivalent to E620 of hRET) from both molecules of $zRET^{ECD}$ within the zRGα1a structure.
(B) Equivalent distance between the Cα E620 from both molecules of $hRET^{ECD}$ from the $hRET^{ECD}$-NRTN-GFRα2 (PDB: 6Q2O) structure.
(C) Equivalent separation between the Cα E620 from the two molecules of $hRET^{ECD}$ from the $hRET^{ECD}$-GDF15-GFRAL (PDB: 6Q2J) structure. The overall structure is represented as a cartoon and the Ca^{2+} ions are represented as spheres. RET is colored cyan, teal, and pale cyan in zRGα1a, $hRET^{ECD}$-NRTN-GFRα2, and $hRET^{ECD}$-GDF15-GFRAL structures, respectively. GFRα1a, GFRα2, and GFRAL are colored green, dark green, and pale green, respectively. GDNF, NRTN, and GDF15 are colored orange, red, and light pink, respectively.

the geometric length of a GFL ligand dimer and the position of the CRD relative to the dyad-axis of GDNF, presumed to sit above the RET transmembrane region.

Structure-function analysis of zRET-GDNF-GFRα1a interaction sites

The importance of each zRET interaction site on ligand-complex assembly was probed by mutation of the GDNF co-receptor at site 1 or GDNF at site 2. Surface residue heatmaps identified the loop-helix α4 element of $zGFR\alpha1a^{D3}$ contributes residues N323, E326, and E327 to the RET-co-receptor interface and are present in most GFRα sequences (Figure 5A). These residues were mutated to alanine, both individually and as a triple mutant. Using solution-based MST, affinity measurements of $zGDNF^{mat.}{}_{WT}$-$zGFR1\alpha1a^{D1-3}{}_{N323A}$ and $zGDNF^{mat.}{}_{WT}$-$zGFR1\alpha1a^{D1-3}{}_{E326A,E327A}$ complexes binding to fluorescently labeled $zRET^{ECD}$ indicated only a modest impact, with a 2-fold decrease in affinity of E326A-E327A, corresponding to a K_D of 0.17 ± 0.039 μM versus 0.090 ± 0.015 μM for $zGDNF^{mat.}{}_{WT}$-$zGFR1\alpha1a^{D1-3}{}_{WT}$ (Figure 5B). However, when combined as a triple mutation, $zGDNF_{wt}$-$zGFR1\alpha1a^{D1-3}{}_{N323A,E326A,E327A}$, a 25-fold reduction in affinity was observed (K_D of 2.35 ± 0.653 μM) (Figure 5B).

To probe the contribution of site 2 interface residues (Figure 5C) L156, Y158, L224, and E220/H222 of $zGDNF^{mat.}$ were selected for mutation to alanine and prepared using insect cells co-expressed with WT $zGFR\alpha1a^{D1-3}$. The L224A and E220A/H222A mutations adversely affected the expression of $zGDNF^{mat.}$ and could not be evaluated. MST was used to test the affinity of $zGDNF^{mat.}{}_{L156A}$-$zGFR\alpha1a^{D1-3}{}_{WT}$ and $zGDNF^{mat.}{}_{Y158A}$-$zGFR\alpha1a^{D1-3}{}_{WT}$ toward $zRET^{ECD}$. A 2-fold decrease in affinity observed for $zGDNF^{mat.}{}_{Y158A}$ toward $zRET^{ECD}$, whereas no substantial loss in affinity was observed for $zGDNF^{mat.}{}_{L156A}$ (Figure 5D). We interpret the minimal effect of these mutations to $zGDNF^{mat.}$ on $zRET^{ECD}$ binding is indicative of a low-affinity interaction site relative to the zCLD(1-3)-$zGFR\alpha1a^{D3}$ site 1. Taken together, the data for zRET loop deletion and targeted zGFR1α and zGDNF mutations point to site 1 being the dominant high-affinity binding site despite both sites being required for ternary complex assembly.

Different D1 domain orientation between GDNF and GDF15 co-receptor complexes

In the zRGα1a cryo-EM structure, the $GFR\alpha1^{D1}$ domain packs against $GFR\alpha1^{D3}$ using a linker with a conserved SPYE motif

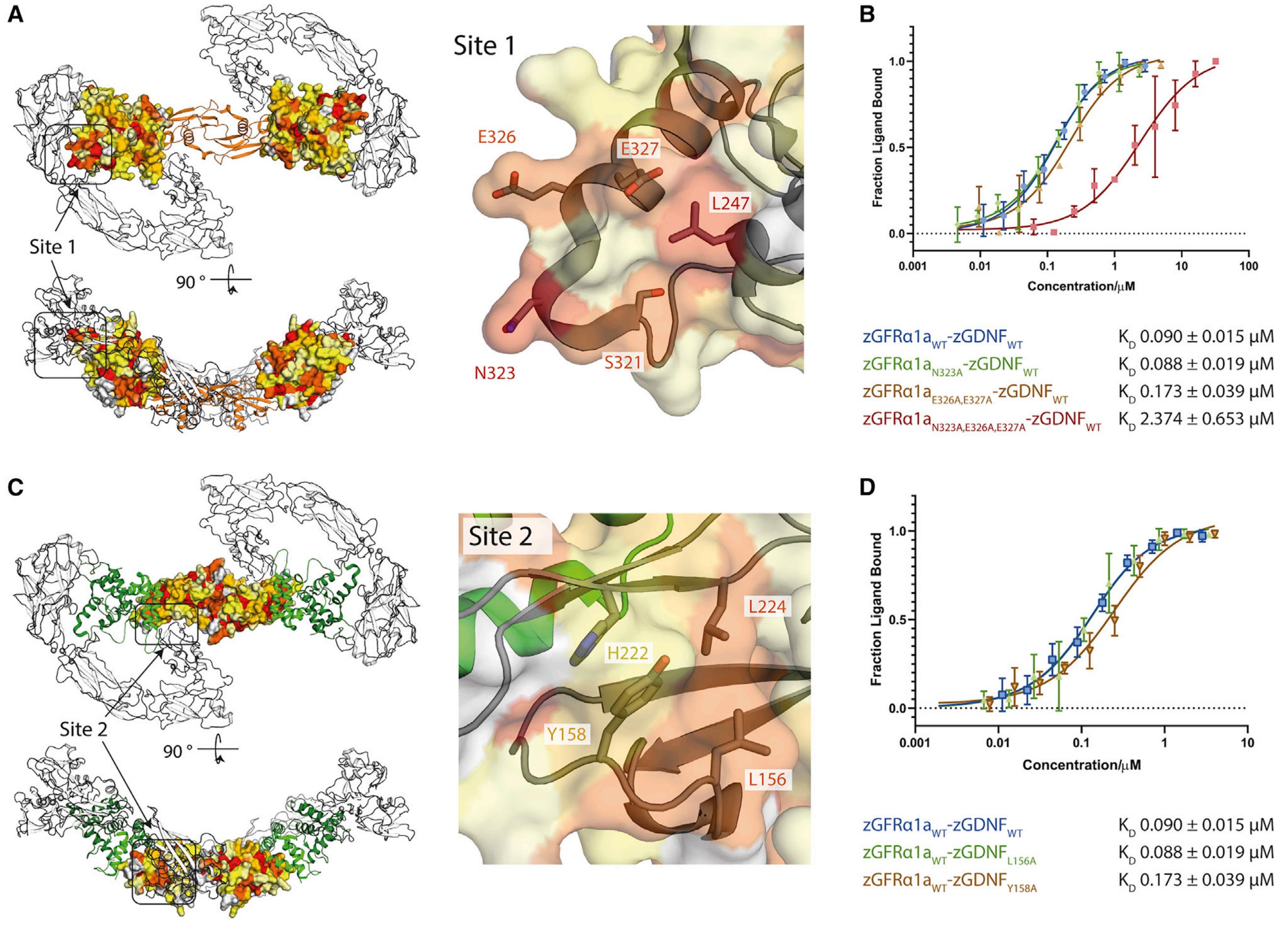

Figure 5. Mutational analysis of zGDNF and zGFRα1 site 1 and 2 interactions with zRET^{ECD}

(A) Heatmap of the sequence conservation between hGFRα paralogs, and zGFRα1a mapped onto the structure of zGFRα1a D2-D3 domains reported here. Residues are colored by similarity (red highly similar to yellow through to white, least similar). Two orthogonal views are shown. Right panel, close-up of site 1 and conserved zGFRα1a residues.

(B) Binding curves and K_D values obtained using MST for zGFRα1a^{D1-3} and mutations assessed in complex with zGDNF$^{mat.}$, with a minimum of n = 3 repeats for the WT and the mutations with the SE represented.

(C) Heatmap of the sequence similarity between GDNF paralogs depicted as a surface representation, mapped onto zGDNF$^{138-235}$. Right panel, close-up of site 2 contact between RET^{CRD} and zGDNF dimer.

(D) MST binding curves and K_D values for zGDNF and mutations L156A and Y158A probed in complex with WT zGFRα1a binding to zRET^{ECD}.

that is retained in all co-receptor sequences except GFRα4 and GFRAL (Figure 6A). We therefore hypothesized that GFRAL^{D1} may require different contacts with RET through a distinctive D1-D2 linker sequence. To explore this possibility, a ternary complex was assembled comprising the hRET^{ECD}, hGDF15$^{mat.}$ (hGDF15$^{195-380}$), and hGFRAL^{D1-3} (hGFRAL$^{18-318}$) (referred to hereafter as hR15AL) (Figure 6B) and crosslinked using GraFix (Figure S7). A low-resolution negative stain envelope was produced with a total of 6,519 particles with C2 symmetry averaging applied (Figures 6C and S7). While the overall shape of the envelope is similar to that of the zRGα1a map with a winged figure-of-eight appearance, it was evident that the wings are at a more acute angle to one another than in the zRGα1a cryo-EM map corresponding to a more "upright" hR15AL complex than the zRGα1a complex (Figure 6C).

Docking the recently published hRET^{ECD}GDF15$^{mat.}$ GFRAL$^{129-318}$ cryo-EM structure (PDB: 6Q2J) (Li et al., 2019) into the low-resolution envelope corroborated the more upright position of both hRET copies. It also revealed substantial density not accounted for by the fitted model, located beneath CLD(1-2) and flanking domains 2 and 3 (D2 and D3) of GFRAL (Figure 6C). The lack of domain 1 (D1) in the fitted model indicates that the area of unoccupied density is most likely the location of GFRAL^{D1} (Figure 6C). Such a position is in marked contrast to that of zGFRα1a^{D1} in zRGα1a (Figure 6D). This indicates a substantial plasticity in GFRAL as the most divergent GFR family member, explaining its lack of sequence conservation within the D1-D2 linker. It also emphasizes further the ability of RET^{ECD} to accommodate a variety of ligand-co-receptor geometries from the flatter ARTN-GFRα3 to the upright GDF15-GFRAL complex, as shown by Li et al. (2019). Some flexibility was apparent within the zGFRα1a$^{\Delta 1}$-zGDNF$^{mat.}$ co-receptor-ligand complex itself and had been noted previously (Parkash and Goldman, 2009). The 2:2 complex X-ray crystal

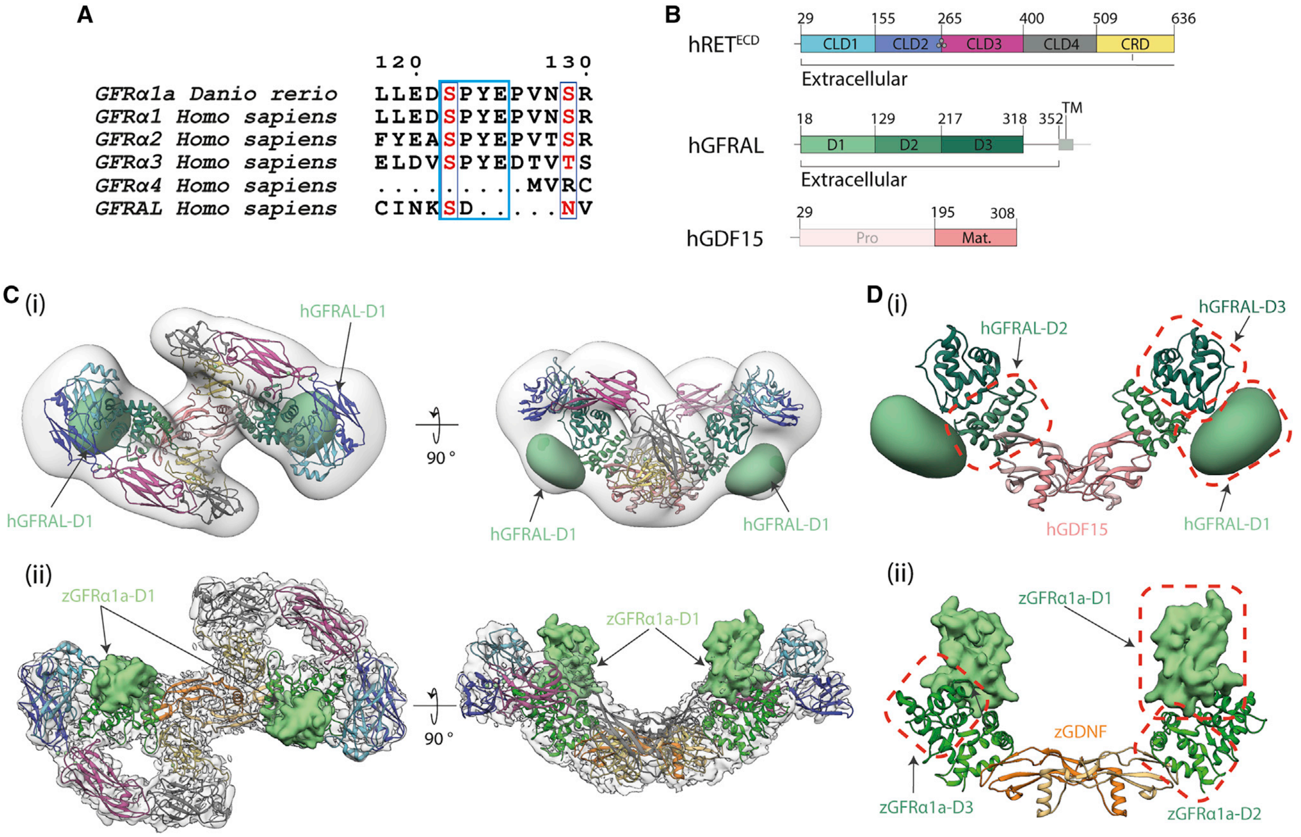

Figure 6. Divergent GFRα1/GFRAL co-receptor D1 domain positions within the RET^{ECD} ternary complex

(A) The D1-D2 domain linker motif (SPYE), highlighted in cyan is conserved between zGFRα1a, GFRα1, GFRα2, and GFRα3. It is missing from the shorter GFRα4 that lacks a D1 domain altogether and from the divergent GFRAL.

(B) Schematic diagram of human RET^{ECD}, GFRAL, and GDF15 construct boundaries used and individual domains annotated as in Figure 1.

(C) (i) Negative stain EM envelope of a reconstituted hRET$^{ECD}_2$-hGDF15$_2$-hGFRAL$_2$ (hR15AL) complex docked with hR15AL (PDB: 6Q2J) revealing additional map potential indicated by a green Gaussian volume (generated from a D1 domain homology model). (ii) Cryo-EM map of zRGα1a (light gray) superposed with the final model (colored as in Figure 2) with GFRα1a^{D1} shown (light green Gaussian volume at 5 Å^2).

(D) Comparison of co-receptor D1 domain position and interfaces (i) GFRAL^{D1} makes different contacts to domains D2-D3 (green), GFRAL^{D1} shown as a 30 Å^2 Gaussian volume (light green), and GDF15 (salmon). (ii) zGFRα1a^{D1} contacts and colored as in Figure 2. zGFRα1a^{D1} represented as a 5 Å^2 Gaussian volume (light green).

structure in the absence of RET^{ECD} has a distance of 127.9 Å between K325 of each GFRα1a protomer whereas in the presence of RET^{ECD} this distance increases to 131.3 Å (Figure S8). Further studies are required to map in detail the additional interactions provided by GFRAL^{D1} to bind RET. We conclude that plasticity is not only evident within RET^{ECD} in accepting different GFL ligand-co-receptor geometries but also points to different roles for domain D1 between paralogs.

Linear arrays of RET^{ECD}-GDNF-GFRα1a observed on cryo-EM grids

Cryo-EM micrographs of the non-crosslinked sample of zRGα1a revealed significant orientation bias of the zRGα1a particles and a single predominant view projecting down the zRGα1a molecular by 2-fold (Figure 7A). Upon closer inspection using RELION particle reposition (Zivanov et al., 2018) a homotypic interaction between zRGα1a particles was observed throughout the grids resulting in linear arrays of complexes (Figure 7A). These arrays can consist of between two and four particles in length. We analyzed 3,756 randomly picked particles from 14 micrographs. Using an interparticle distance of 214.2 Å (170 pixels) from the centroid of one particle to the centroid of neighboring particles (x, y coordinates from the star file), 4,132 particle pairs were defined. A 3D surface distribution plot of the difference in psi angles (Δψ) for pairs of particles against the distance between their centroids was calculated (Figure 7B), the ψ angles are generated in RELION 2D classification (Kimanius et al., 2016; Zivanov et al., 2018). An error of 3° exists within the plot due to the angular sampling value used during 2D classification. The 3D plot revealed that particles at a distance of 181 ± 3 Å from one another have an average Δψ of 4.5° ± 2.3°, using a minimal frequency of Δψ to average distance of the more than 0.5 (Figure 7B). The recurrent and repetitive nature of this end-to-end contact for neighboring particle pairs was further captured in a 2D class average, which used 1,194 particle pairs (2,388 individual particles) (Figure 7C).

Using the information gathered from the particle pair analysis, two copies of the zRGα1a complex structure were aligned with

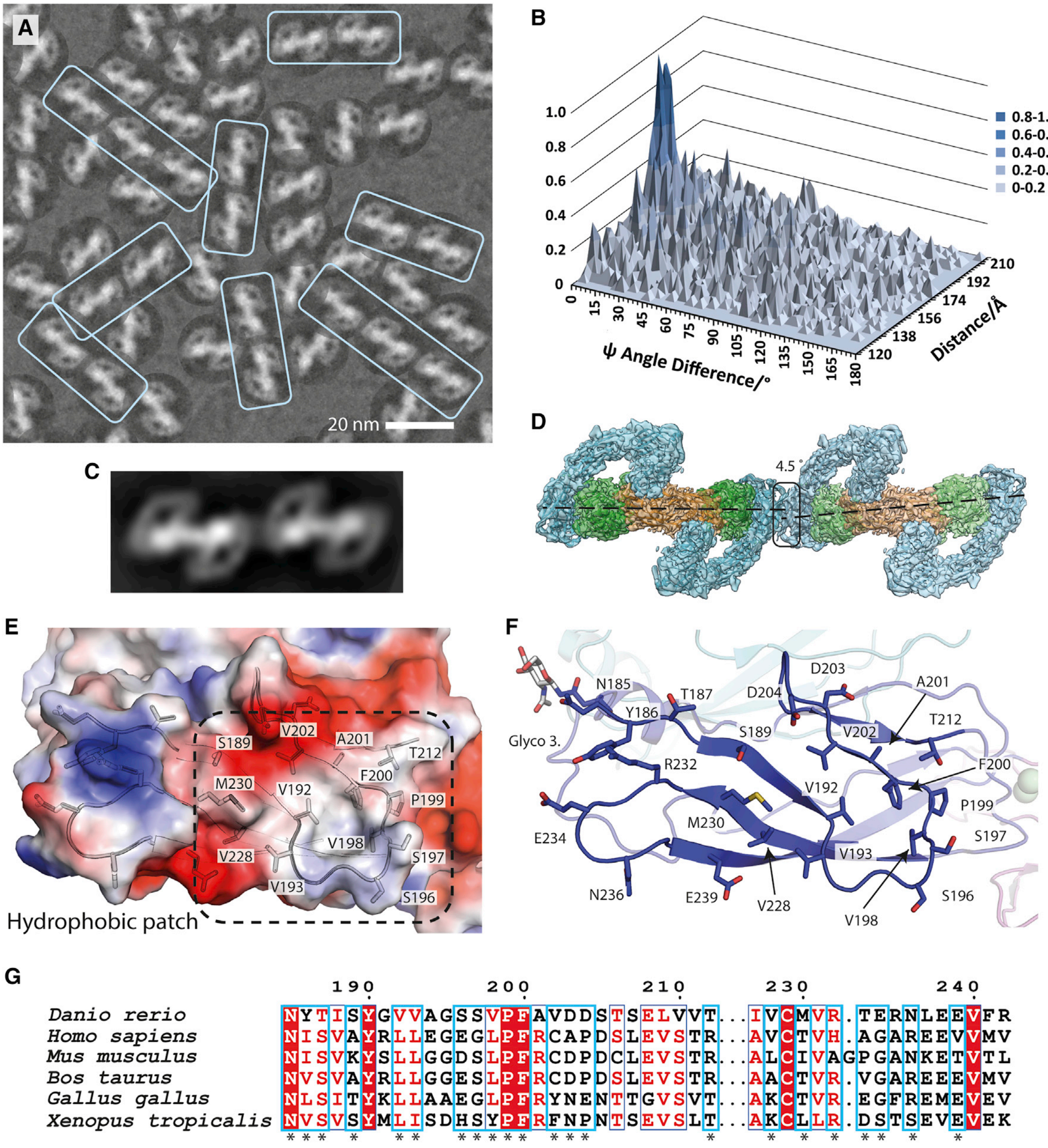

Figure 7. Evidence for linear arrays of zRGα1a particles on cryo-EM grids

(A) Close-up of a representative micrograph for zRGα1a showing the particle orientation bias by fitting the dominant 2D class average view into picked particles and a recurring linear array of particles highlighted within pale cyan boxes.

(B) Statistical distribution of the difference between the angle psi (Δψ) between two particles and their separation distance. Here the angle ψ is defined for each particle as the angle of rotation of each particle required to align it onto the 2D class average.

(C) 2D class average from automated particle picking containing two adjacent zRGα1a particles.

(D) The zRGα1a-zRGα1a interface highlighted with a black box. The angle and separation between each complex is based on the peak maxima coordinates from (B) assuming both particles are at the same Z height.

(E) An electrostatic potential surface with selected side chains for the homotypic zRET^{CLD2} interface.

(F) Close-up of the CLD2 contact, highlighting interface residues.

(G) Conservation of representative RET sequences at the CLD2-CLD2 interface shown with an asterix.

an interparticle distance ~180 Å apart and an angle of 4.5° between the two copies (Figure 7D). Observations of both the single particle as well as the 2D class averages generated for a pair of zRGα1a complexes show that the two wings of the figure-of-eight structure do not appear to be symmetrical, with a slightly more acute angle appearing between zGFRα1a and zGDNF on the sides in contact with one another in the neighboring particles (Figures 7A and 7C). The interparticle interaction site observed

CellPress
OPEN ACCESS

on cryo-EM grids maps to a predominantly hydrophobic surface of CLD2, centered on V192 and V202 (Figures 7E and 7F). This hydrophobic patch is conserved between lower and higher vertebrates and is flanked by both basic (R232) and acidic clusters (D203, D204, and E239) that reciprocally neutralize each other across the zRGα1a-zRGα1a interface (Figure 7G). We note a highly conserved glycosylation site at N185 of CLD2 (found in both higher and lower vertebrates) is situated on the periphery of the multimerization interface (Figure 7G, glyco-site 3). In a linear array context, this glycan could potentially interact with calcium ion Ca-1 (near the CLD(2-3) junction) of an adjacent ternary complex to complete its coordination shell in *trans*. Further analyses are required to demonstrate a functional role for this multimeric interaction for full-length RET in a cellular context. Nevertheless, the high-sequence conservation within the interface points to an important role beyond ligand-co-receptor interaction.

DISCUSSION

Here, we establish principles for understanding the assembly of RET ligand-co-receptor complexes. We rationalize how RET can accept a range of activating GFL-co-receptor binary complexes through conformational adaptations between RET and co-receptor. By using crystallography and cryo-EM we define the architecture and ligand recognition properties of zebrafish RET^{ECD} and compare this with the human RET^{ECD}. Our results provide four main insights; (1) there is conformational flexibility at the CLD(2-3) interface of RET^{ECD} that contributes to optimized adaptations at the co-receptor binding site; (2) there are conformational differences between unliganded and liganded RET centered on a clade-specific RET loop; (3) a strict spatial separation of RET^{ECD} C termini within the ternary complex is imposed by each CRD interaction with a GFL dimer; (4) differences in co-receptor engagement and putative higher-order multimers of ligand-bound RET suggest divergent interactions at each level of receptor engagement.

Previous insights into GFL-co-receptor recognition from negative stain and cryo-EM have revealed two main contact sites in RET (Bigalke et al., 2019; Goodman et al., 2014; Li et al., 2019). These structures explained why an intact calcium-loaded RET^{ECD} is required for GDNF-GFRα1 binding as the $GFR\alpha1^{D3}$ loop-helix α4/$GFR\alpha1^{D2}$ helix α1 wedge targets the calcium-dependent CLD(2-3) hinge while the GDNF dimer targets the CRD. The GFRα1 wedge may act as a sensor for calcium bound to RET implicating calcium not only in promoting RET folding but also proper recognition by co-receptor for signaling (Nozaki et al., 1998). The RET^{CRD} interaction with both protomers of a GDNF dimer is directly equivalent to the binding site of "low-affinity" TGF-β/BMP family of type 1 receptors for TGF-β ("knuckles" and "thumb") (Hinck et al., 2016). Whereas the TGF-β "fingers" engage the high-affinity TGF-β receptor, equivalent to GFRα co-receptors binding to GFL fingers.

Several studies identified a role for site 1 contacts close to N323 in RET ternary complex formation (Goodman et al., 2014; Bigalke et al., 2019; Li et al., 2019). The strikingly distinctive contacts made by different GFRα homologs at site 1 suggest conformational adaptations enable the recognition of multiple GFRα co-receptors and different GFR_2GFL_2 geometries. Our findings suggest that engagement of ligand-co-receptor through the calcium-dependent CLD(2-3) hinge promotes a remodeling of the lower-vertebrate-specific loop and may precede site 2 RET^{CRD} engagement. This could involve either a pre-assembled RET-GFRα complex or presentation of GFRα after dimerization by GFL, before RET^{CRD} interaction. We show here from substitution of zGDNF residues in site 2 (L156A and Y158A) that these contacts do not appear to play a dominant role in ternary complex assembly. This contrasts with a study showing mutation of Y119 to E in Neurturin (equivalent to Y158 of zGDNF) disrupted ternary complex formation and signaling (Bigalke et al., 2019). Given the analogous RET^{CRD} contacts at site 2 for each GFL dimer are proximal to the RET transmembrane segment, this suggests an organizing role for signal transduction in addition to contributing to ligand recognition.

The D1 domain is missing from previous structures of GDNF-GFRα1 and GDF15-GFRAL, but had been observed for NRTN-GFRα2 alone or bound to $hRET^{ECD}$ (Bigalke et al., 2019; Li et al., 2019). We were able to place the GFRα1 domain D1 adjacent to $zRET^{CLD1}$, consistent with previous negative stain EM models (Goodman et al., 2014). As previously shown, the D1 proximity to RET^{CLD1} is not essential for ternary complex formation. We present evidence for a quite different contact position for the GFRAL D1 domain adjacent to GFRAL D2 and D3 domains, on the outside of RET and underneath its "wings." This explains the absence of the otherwise conserved SPYE motif common to GFRα1/2/3 motifs at the D1 and D3 interface. This position for the GFRAL D1 domain arises from a more upright position for GFRAL observed than GDNF-GFRα1 complexes (Li et al., 2019). While the functional significance of this difference is yet to be understood, it could impact on ligand-biased signaling outputs or the assembly of higher-order multimers, such as those observed for zRGα1a.

We and others have provided structural evidence for RET dimers in the absence of a ligand-co-receptor through a CLD1-2 dimer interface involving R77 and R144 side chains (Kjær et al., 2010; Li et al., 2019). Here, we describe a ligand-dependent linear array of zGDNF-zGFRα1a-$zRET^{ECD}$ complexes observed throughout the cryo-EM micrographs. This dominant mode of multimerization observed on micrographs is mediated by a homotypic hydrophobic patch on an exposed part of CLD2 (CLD2-CLD2 interface). The arrangement is distinct from but compatible with the "stacked" interaction observed between two hNRTN-hGFRα2-$hRET^{ECD}$ ternary complexes by Li et al., 2019. The stacked interaction involved contacts between $hRET^{CLD4}$ and NRTN and was reported to influence the rate of receptor endocytosis. We do observe occasional stacked particles packed in this manner but we cannot conclude their significance at this point and the $zRET^{CLD4}$ stacked interface is not conserved. These findings suggest that a signaling-competent RET^{ECD} conformation is likely to involve higher-order multimers consistent with findings for other RTKs, such as EphR (Seiradake et al., 2010), EGFR (Needham et al., 2016), and DDR1 (Corcoran et al., 2019) RTKs. Therefore, a crucial aspect of receptor activation beyond the positioning of the RET transmembrane regions within a dimeric assembly may prove to be their arrangement within higher-order clusters.

In summary, this study reveals several under-appreciated aspects of GFL-co-receptor binding to RET, including receptor

flexibility, clade-specific adaptations, and conformational changes. All these features reveal a substantial tolerance within RET to accommodate different GFL-co-receptors using a flexible arm. It also suggests that a key requirement for coupling ligand binding to RET activation is a strict spatial separation between CRD C termini within RET dimers imposed by the geometric dimensions of each GDNF family ligand. The next challenge will be to visualize such arrangements of a full-length RET multimer in a membrane context and to use this knowledge in the design of both antagonist and agonist biologicals that with therapeutic utility.

STAR★METHODS

Detailed methods are provided in the online version of this paper and include the following:

- KEY RESOURCES TABLE
- RESOURCE AVAILABILITY
  - Lead contact
  - Materials availability
  - Data and code availability
- EXPERIMENTAL MODEL AND SUBJECT DETAILS
- METHOD DETAILS
  - Zebrafish RET CLD(1-4) expression and purification
  - zCLD(1-4)$^{red.sug.}$ crystallisation and X-ray data collection
  - zCLD(1-4)$^{red.sug.}$ x-ray data processing and structure determination
  - zGDNF$^{mat.}$-zGFRα1a$^{D1\text{-}3}$ expression and purification
  - zGDNF$^{mat.}$-zGFRα1a$^{D1\text{-}3}$ crystallisation and structure determination
  - zRET^{ECD}-zGDNF$^{mat.}$-zGFRα1a$^{D1\text{-}3}$- (zRGα1a) complex expression and purification
  - zRGα1a cryo-electron microscopy sample preparation
  - Cryo-EM data acquisition: Datasets 1 to 3
  - Cryo-EM data processing of crosslinked zRGα1a (dataset 1)
  - Cryo-EM data processing of crosslinked zRGα1a (tilted dataset 2)
  - Combining and processing cryo-EM datasets 1 and 2 for crosslinked zRGα1a
  - Building the zRGα1a complex into the final cryo-EM map
  - Cryo-EM data processing for a non-crosslinked zRGα1a sample (dataset 3)
  - Analysis of zRGα1a multimer formation on cryo-EM grids
  - Human RET^{ECD} expression and purification
  - Human GDF15$^{mat.}$-GFRAL$^{D1\text{-}3}$ complex expression and purification
  - hRET^{ECD}-hGDF15$^{mat.}$-hGFRAL$^{D1\text{-}3}$ (hR15AL) complex assembly and purification
  - hR15AL negative stain preparation, data acquisition and processing
  - Microscale thermophoresis (MST) measurement of zRET^{ECD} binding affinity
  - Surface conservation analysis and heatmaps for different GFL-GFR ligand-coreceptor pairs
- QUANTIFICATION AND STATISTICAL ANALYSIS

SUPPLEMENTAL INFORMATION

Supplemental information can be found online at https://doi.org/10.1016/j.str.2020.12.012.

ACKNOWLEDGMENTS

We thank members of the McDonald laboratory for helpful comments. We thank Simone Kunzelmann for help with MST experiments. We thank Raffaella Carzaniga and Lucy Collinson for EM training. We thank Alessandro Costa and Tom Miller for their advice on the particle replacement analysis. We acknowledge the advice and support of Peter Rosenthal. We thank Carlos Ibanez for critical reading. We thank Diamond Light Source for beamtime (proposals mx13775) and the staff of beamlines I03 and I04 for their assistance with data collection. N.Q.McD. acknowledges support from the Francis Crick Institute, which receives its core funding from Cancer Research UK (FC001115), the UK Medical Research Council (FC001115), and the Wellcome Trust (FC001115). For the purpose of Open Access, the author has applied a CC BY public copyright licence to any Author Accepted Manuscript version arising from this submission.

AUTHOR CONTRIBUTIONS

S.E.A. prepared the zRGα1a complex, carried out EM data processing, crystallized the GDNF-GFRα1a complex, and performed the MST assays. A.G.P. phased the zRET$^{CLD1\text{-}4}$ X-ray crystal structure and built the structural model. P.P.K. grew and optimized the CLD(1-4) crystals. K.M.G. performed initial CLD(1-4) expression and crystallization and A. Nawrotek performed CLD(1-4) crystallization optimization. A. Nans collected the zRGα1a Krios datasets. A. Borg and S.K. prepared the hRET^{ECD}. D.C.B. expressed and purified the hRET^{ECD}-hGDF15-hGFRAL complex. C.P.E. assisted in cryo-EM sample optimization, EM data processing, and model refinement. S.E.A. crosslinked the hR15AL complex, and collected and processed the EM data. A.J. Borg performed the XL-MS experiments. F.M.H. expressed some of the zGFRα1 mutants used for MST. P.B.McI. collected the native non-crosslinked zRGα1a dataset.

DECLARATION OF INTERESTS

The authors declare no competing financial interests.

Received: October 13, 2020
Revised: December 7, 2020
Accepted: December 18, 2020
Published: January 22, 2021

REFERENCES

Abagyan, R., Totrov, M., and Kuznetsov, D. (1994). ICM—a new method for protein modeling and design: applications to docking and structure prediction from the distorted native conformation. J. Comput. Chem. *15*, 488–506.

Adams, P.D., Afonine, P.V., Bunkóczi, G., Chen, V.B., Davis, I.W., Echols, N., Headd, J.J., Hung, L.W., Kapral, G.J., Grosse-Kunstleve, R.W., et al. (2010). PHENIX: a comprehensive Python-based system for macromolecular structure solution. Acta Crystallogr. D Biol. Crystallogr. *66*, 213–221.

Afonine, P.V., Grosse-Kunstleve, R.W., Echols, N., Headd, J.J., Moriarty, N.W., Mustyakimov, M., Terwilliger, T.C., Urzhumtsev, A., Zwart, P.H., and Adams, P.D. (2012). Towards automated crystallographic structure refinement with phenix.refine. Acta Crystallogr. D Biol. Crystallogr. *68*, 352–367.

Afonine, P.V., Poon, B.K., Read, R.J., Sobolev, O.V., Terwilliger, T.C., Urzhumtsev, A., and Adams, P.D. (2018). Real-space refinement in PHENIX for cryo-EM and crystallography. Acta Crystallogr. D Struct. Biol. *74*, 531–544.

Agirre, J., Iglesias-Fernández, J., Rovira, C., Davies, G.J., Wilson, K.S., and Cowtan, K.D. (2015). Privateer: software for the conformational validation of carbohydrate structures. Nat. Struct. Mol. Biol. *22*, 833–834.

Airaksinen, M.S., and Saarma, M. (2002). The GDNF family: signalling, biological functions and therapeutic value. Nat. Rev. Neurosci. *3*, 383–394.

CellPress
OPEN ACCESS

Allen, S.J., Watson, J.J., Shoemark, D.K., Barua, N.U., and Patel, N.K. (2013). GDNF, NGF and BDNF as therapeutic options for neurodegeneration. Pharmacol. Ther. *138*, 155–175.

An, J., Totrov, M., and Abagyan, R. (2005). Pocketome via comprehensive identification and classification of ligand binding envelopes. Mol. Cell Proteomics *4*, 752–761.

Anders, J., Kjær, S., and Ibáñez, C.F. (2001). Molecular modeling of the extracellular domain of the RET receptor tyrosine kinase reveals multiple cadherin-like domains and a calcium-binding site. J. Biol. Chem. *276*, 35808–35817.

Attie, T., Pelet, A., Edery, P., Eng, C., Mulligan, L.M., Amiel, J., Boutrand, L., Beldjord, C., Nihoul-Fekete, C., Munnich, A., et al. (1995). Diversity of RET proto-oncogene mutations in familial and sporadic Hirschsprung disease. Hum. Mol. Genet. *4*, 1381–1386.

Baloh, R.H., Tansey, M.G., Golden, J.P., Creedon, D.J., Heuckeroth, R.O., Keck, C.L., Zimonjic, D.B., Popescu, N.C., Johnson, E.M., Jr., and Milbrandt, J. (1997). TrnR2, a novel receptor that mediates neurturin and GDNF signaling through Ret. Neuron *18*, 793–802.

Baloh, R.H., Gorodinsky, A., Golden, J.P., Tansey, M.G., Keck, C.L., Popescu, N.C., Johnson, E.M., Jr., and Milbrandt, J. (1998a). GFRα3 is an orphan member of the GDNF/neurturin/persephin receptor family. Proc. Natl. Acad. Sci. U S A *95*, 5801–5806.

Baloh, R.H., Tansey, M.G., Lampe, P.A., Fahrner, T.J., Enomoto, H., Simburger, K.S., Leitner, M.L., Araki, T., Johnson, E.M., Jr., and Milbrandt, J. (1998b). Artemin, a novel member of the GDNF ligand family, supports peripheral and central neurons and signals through the GFRα3-RET receptor complex. Neuron *21*, 1291–1302.

Bigalke, J.M., Aibara, S., Roth, R., Dahl, G., Gordon, E., Dorbéus, S., Amunts, A., and Sandmark, J. (2019). Cryo-EM structure of the activated RET signaling complex reveals the importance of its cysteine-rich domain. Sci. Adv. *5*, eaau4202.

Boggon, T.J., Murray, J., Chappuis-Flament, S., Wong, E., Gumbiner, B.M., and Shapiro, L. (2002). C-Cadherin ectodomain structure and implications for cell adhesion mechanisms. Science *296*, 1308–1313.

Brasch, J., Harrison, O.J., Honig, B., and Shapiro, L. (2012). Thinking outside the cell: how cadherins drive adhesion. Trends Cell Biol. *22*, 299–310.

Cacalano, G., Fariñas, I., Wang, L.C., Hagler, K., Forgie, A., Moore, M., Armanini, M., Phillips, H., Ryan, A.M., Reichardt, L.F., et al. (1998). GFRα1 is an essential receptor component for GDNF in the developing nervous system and kidney. Neuron *21*, 53–62.

Collaborative Computational Project, Number 4 (1994). The CCP4 suite: programs for protein crystallography. Acta Crystallogr. D Biol. Crystallogr. *50*, 760–763.

Combe, C.W., Fischer, L., and Rappsilber, J. (2015). xiNET: cross-link network maps with residue resolution. Mol. Cell Proteomics *14*, 1137–1147.

Corcoran, D.S., Juskaite, V., Xu, Y., Gorlitz, F., Alexandrov, Y., Dunsby, C., French, P.M.W., and Leitinger, B. (2019). DDR1 autophosphorylation is a result of aggregation into dense clusters. Sci. Rep. *9*, 17104.

Durbec, P., Marcos-Gutierrez, C.V., Kilkenny, C., Grigoriou, M., Wartiowaara, K., Suvanto, P., Smith, D., Ponder, B., Costantini, F., Saarma, M., et al. (1996). GDNF signalling through the Ret receptor tyrosine kinase. Nature *381*, 789–793.

Emmerson, P.J., Wang, F., Du, Y., Liu, Q., Pickard, R.T., Gonciarz, M.D., Coskun, T., Hamang, M.J., Sindelar, D.K., Ballman, K.K., et al. (2017). The metabolic effects of GDF15 are mediated by the orphan receptor GFRAL. Nat. Med. *23*, 1215–1219.

Emsley, P., and Cowtan, K. (2004). Coot: model-building tools for molecular graphics. Acta Crystallogr. D Biol. Crystallogr. *60*, 2126–2132.

Emsley, P., Lohkamp, B., Scott, W.G., and Cowtan, K. (2010). Features and development of Coot. Acta Crystallogr. D Biol. Crystallogr. *66*, 486–501.

Fernandez-Recio, J., Totrov, M., Skorodumov, C., and Abagyan, R. (2005). Optimal docking area: a new method for predicting protein-protein interaction sites. Proteins Struct. Funct. Genet. *58*, 134–143.

Goodman, K.M., Kjær, S., Beuron, F., Knowles, P.P., Nawrotek, A., Burns, E.M., Purkiss, A.G., George, R., Santoro, M., Morris, E.P., et al. (2014). RET recognition of GDNF-GFRα1 ligand by a composite binding site promotes membrane-proximal self-association. Cell Rep. *8*, 1894–1904.

Groppe, J., Hinck, C.S., Samavarchi-Tehrani, P., Zubieta, C., Schuermann, J.P., Taylor, A.B., Schwarz, P.M., Wrana, J.L., and Hinck, A.P. (2008). Cooperative assembly of TGF-β superfamily signaling complexes is mediated by two disparate mechanisms and distinct modes of receptor binding. Mol. Cell *29*, 157–168.

Henderson, C.E., Phillips, H.S., Pollock, R.A., Davies, A.M., Lemeulle, C., Armanini, M., Simpson, L.C., Moffet, B., Vandlen, R.A., Koliatsos, V.E., et al. (1994). GDNF: a potent survival factor for motoneurons present in peripheral nerve and muscle. Science *266*, 1062–1064.

Hinck, A.P., Mueller, T.D., and Springer, T.A. (2016). Structural biology and evolution of the TGF-β family. Cold Spring Harb. Perspect. Biol. *8*, a022103.

Hsu, J.Y., Crawley, S., Chen, M., Ayupova, D.A., Lindhout, D.A., Higbee, J., Kutach, A., Joo, W., Gao, Z., Fu, D., et al. (2017). Non-homeostatic body weight regulation through a brainstem-restricted receptor for GDF15. Nature *550*, 255–259.

Ibáñez, C.F. (2013). Structure and physiology of the RET receptor tyrosine kinase. Cold Spring Harb. Perspect. Biol. *5*, a009134.

Jing, S., Wen, D., Yu, Y., Holst, P.L., Luo, Y., Fang, M., Tamir, R., Antonio, L., Hu, Z., Cupples, R., et al. (1996). GDNF-induced activation of the Ret protein tyrosine kinase is mediated by GDNFR-α, a novel receptor for GDNF. Cell *85*, 1113–1124.

Kao, A., Chiu, C.L., Vellucci, D., Yang, Y., Patel, V.R., Guan, S., Randall, A., Baldi, P., Rychnovsky, S.D., and Huang, L. (2011). Development of a novel cross-linking strategy for fast and accurate identification of cross-linked peptides of protein complexes. Mol. Cell Proteomics *10*, M110.002212.

Kastner, B., Fischer, N., Golas, M.M., Sander, B., Dube, P., Boehringer, D., Hartmuth, K., Deckert, J., Hauer, F., Wolf, E., et al. (2008). GraFix: sample preparation for single-particle electron cryomicroscopy. Nat. Methods *5*, 53–55.

Kimanius, D., Forsberg, B.O., Scheres, S.H.W., and Lindahl, E. (2016). Accelerated cryo-EM structure determination with parallelisation using GPUs in RELION-2. eLife *5*, e18722.

Kirsch, T., Sebald, W., and Dreyer, M.K. (2000). Crystal structure of the BMP-2-BRIA ectodomain complex. Nat. Struct. Biol. *7*, 492–496.

Kjær, S., and Ibáñez, C.F. (2003). Identification of a surface for binding to the GDNF-GFRα1 complex in the first cadherin-like domain of RET. J. Biol. Chem. *278*, 47898–47904.

Kjær, S., Hanrahan, S., Totty, N., and McDonald, N.Q. (2010). Mammal-restricted elements predispose human RET to folding impairment by HSCR mutations. Nat. Struct. Mol. Biol. *17*, 726–731.

Koch, A.W., Farooq, A., Shan, W., Zeng, L., Colman, D.R., and Zhou, M.M. (2004). Structure of the neural (N-)cadherin prodomain reveals a cadherin extracellular domain-like fold without adhesive characteristics. Structure *12*, 793–805.

Kohfeldt, E., Maurer, P., Vannahme, C., and Timpl, R. (1997). Properties of the extracellular calcium binding module of the proteoglycan testican. FEBS Lett. *414*, 557–561.

Kotzbauer, P.T., Lampe, P.A., Heuckeroth, R.O., Golden, J.P., Creedon, D.J., Johnson, E.M., Jr., and Milbrandt, J. (1996). Neurturin, a relative of glial-cell-line-derived neurotrophic factor. Nature *384*, 467–470.

Krissinel, E., and Henrick, K. (2007). Inference of macromolecular assemblies from crystalline state. J. Mol. Biol. *372*, 774–797.

Lemmon, M.A., and Schlessinger, J. (2010). Cell signaling by receptor tyrosine kinases. Cell *141*, 1117–1134.

Li, J., Shang, G., Chen, Y.J., Brautigam, C.A., Liou, J., Zhang, X., and Bai, X.C. (2019). Cryo-EM analyses reveal the common mechanism and diversification in the activation of RET by different ligands. eLife *8*, e47650.

Liu, F., Rijkers, D.T.S., Post, H., and Heck, A.J.R. (2015). Proteome-wide profiling of protein assemblies by cross-linking mass spectrometry. Nat. Methods *12*, 1179–1184.

McCoy, A.J., Grosse-Kunstleve, R.W., Adams, P.D., Winn, M.D., Storoni, L.C., and Read, R.J. (2007). Phaser crystallographic software. J. Appl. Crystallogr. *40*, 658–674.

Milbrandt, J., De Sauvage, F.J., Fahrner, T.J., Baloh, R.H., Leitner, M.L., Tansey, M.G., Lampe, P.A., Heuckeroth, R.O., Kotzbauer, P.T., Simburger, K.S., et al. (1998). Persephin, a novel neurotrophic factor related to GDNF and neurturin. Neuron *20*, 245–253.

Mullican, S.E., Lin-Schmidt, X., Chin, C.N., Chavez, J.A., Furman, J.L., Armstrong, A.A., Beck, S.C., South, V.J., Dinh, T.Q., Cash-Mason, T.D., et al. (2017). GFRAL is the receptor for GDF15 and the ligand promotes weight loss in mice and nonhuman primates. Nat. Med. *23*, 1150–1157.

Mulligan, L.M. (2014). RET revisited: expanding the oncogenic portfolio. Nat. Rev. Cancer *14*, 173–186.

Nagar, B., Overduin, M., Ikura, M., and Rini, J.M. (1996). Structural basis of calcium-induced E-cadherin rigidification and dimerization. Nature *380*, 360–364.

Needham, S.R., Roberts, S.K., Arkhipov, A., Mysore, V.P., Tynan, C.J., Zanetti-Domingues, L.C., Kim, E.T., Losasso, V., Korovesis, D., Hirsch, M., et al. (2016). EGFR oligomerization organizes kinase-active dimers into competent signalling platforms. Nat. Commun. *7*, 13307.

Nozaki, C., Asai, N., Murakami, H., Iwashita, T., Iwata, Y., Horibe, K., Klein, R.D., Rosenthal, A., and Takahashi, M. (1998). Calcium-dependent Ret activation by GDNF and neurturin. Oncogene *16*, 293–299.

Parkash, V., and Goldman, A. (2009). Comparison of GFL-GFRα complexes: further evidence relating GFL bend angle to RET signalling. Acta Crystallogr. Sect. F Struct. Biol. Cryst. Commun. *F65*, 551–558.

Pelet, A., Geneste, O., Edery, P., Pasini, A., Chappuis, S., Attié, T., Munnich, A., Lenoir, G., Lyonnet, S., and Billaud, M. (1998). Various mechanisms cause RET-mediated signaling defects in Hirschsprung's disease. J. Clin. Invest. *101*, 1415–1423.

Pettersen, E.F., Goddard, T.D., Huang, C.C., Couch, G.S., Greenblatt, D.M., Meng, E.C., and Ferrin, T.E. (2004). UCSF Chimera—a visualization system for exploratory research and analysis. J. Comput. Chem. *25*, 1605–1612.

Punjani, A., Rubinstein, J.L., Fleet, D.J., and Brubaker, M.A. (2017). CryoSPARC: algorithms for rapid unsupervised cryo-EM structure determination. Nat. Methods *14*, 290–296.

Robert, X., and Gouet, P. (2014). Deciphering key features in protein structures with the new ENDscript server. Nucleic Acids Res. *42*, W320–W324.

Rohou, A., and Grigorieff, N. (2015). CTFFIND4: fast and accurate defocus estimation from electron micrographs. J. Struct. Biol. *192*, 216–221.

de la Rosa-Trevín, J.M., Otón, J., Marabini, R., Zaldívar, A., Vargas, J., Carazo, J.M., and Sorzano, C.O.S. (2013). Xmipp 3.0: an improved software suite for image processing in electron microscopy. J. Struct. Biol. *184*, 321–328.

de la Rosa-Trevín, J.M., Quintana, A., del Cano, L., Zaldívar, A., Foche, I., Gutiérrez, J., Gómez-Blanco, J., Burguet-Castell, J., Cuenca-Alba, J., Abrishami, V., et al. (2016). Scipion: a software framework toward integration, reproducibility and validation in 3D electron microscopy. J. Struct. Biol. *195*, 93–99.

Rubinstein, R., Thu, C.A., Goodman, K.M., Wolcott, H.N., Bahna, F., Mannepalli, S., Ahlsen, G., Chevee, M., Halim, A., Clausen, H., et al. (2015). Molecular logic of neuronal self-recognition through protocadherin domain interactions. Cell *163*, 629–642.

Sandmark, J., Dahl, G., Öster, L., Xu, B., Johansson, P., Akerud, T., Aagaard, A., Davidsson, P., Bigalke, J.M., Winzell, M.S., et al. (2018). Structure and biophysical characterization of the human full-length neurturin-GFRα2 complex: a role for heparan sulfate in signaling. J. Biol. Chem. *293*, 5492–5508.

Scheres, S.H.W. (2012). RELION: implementation of a Bayesian approach to cryo-EM structure determination. J. Struct. Biol. *180*, 519–530.

Schrodinger, LLC. (2015). The PyMOL Molecular Graphics System, Version 1.8.

Schwede, T., Kopp, J., Guex, N., and Peitsch, M.C. (2003). SWISS-MODEL: an automated protein homology-modeling server. Nucleic Acids Res. *31*, 3381–3385.

Seiradake, E., Harlos, K., Sutton, G., Aricescu, A.R., and Jones, E.Y. (2010). An extracellular steric seeding mechanism for Eph-ephrin signaling platform assembly. Nat. Struct. Mol. Biol. *17*, 398–402.

Shapiro, L., and Weis, W.I. (2009). Structure and biochemistry of cadherins and catenins. Cold Spring Harb. Perspect. Biol. *1*, a003053.

Shapiro, L., Fannon, A.M., Kwong, P.D., Thompson, A., Lehmann, M.S., Gerhard, G., Legrand, J.F., Als-Nielsen, J., Colman, D.R., and Hendrickson, W.A. (1995). Structural basis of cell-cell adhesion by cadherins. Nature *374*, 327–337.

Sievers, F., Wilm, A., Dineen, D., Gibson, T.J., Karplus, K., Li, W., Lopez, R., McWilliam, H., Remmert, M., Söding, J., et al. (2011). Fast, scalable generation of high-quality protein multiple sequence alignments using Clustal Omega. Mol. Syst. Biol. *7*, 539.

So, M.T., LeonThomas, Y.Y., Cheng, G., TangClara, S.M., Miao, X.P., Cornes, B.K., Ngo, D.N., Cui, L., NganElly, S.W., LuiVincent, C.H., et al. (2011). RET mutational spectrum in Hirschsprung disease: evaluation of 601 Chinese patients. PLoS One *6*, e28986.

Tan, Y.Z., Baldwin, P.R., Davis, J.H., Williamson, J.R., Potter, C.S., Carragher, B., and Lyumkis, D. (2017). Addressing preferred specimen orientation in single-particle cryo-EM through tilting. Nat. Methods *14*, 793–796.

Terwilliger, T.C., Grosse-Kunstleve, R.W., Afonine, P.V., Moriarty, N.W., Zwart, P.H., Hung, L.W., Read, R.J., and Adams, P.D. (2007). Iterative model building, structure refinement and density modification with the PHENIX AutoBuild wizard. Acta Crystallogr. D Biol. Crystallogr. *64*, 61–69.

Thompson, J., Doxakis, E., Piñón, L.G.P., Strachan, P., Buj-Bello, A., Wyatt, S., Buchman, V.L., and Davies, A.M. (1998). GFRα-4, a new GDNF family receptor. Mol. Cell Neurosci. *11*, 117–126.

Tickle, I.J., Flensburg, C., Keller, P., Paciorek, W., Sharff, A., Vonrhein, C., and Bricogne, G. (2018). STARANISO (Global Phasing Ltd). http://staraniso.globalphasing.org/cgi-bin/staraniso.cgi.

Treanor, J.J.S., Goodman, L., De Sauvage, F., Stone, D.M., Poulsen, K.T., Beck, C.D., Gray, C., Armanini, M.P., Pollock, R.A., Hefti, F., et al. (1996). Characterization of a multicomponent receptor for GDNF. Nature *382*, 80–83.

Waterman, D.G., Winter, G., Gildea, R.J., Parkhurst, J.M., Brewster, A.S., Sauter, N.K., and Evans, G. (2016). Diffraction-geometry refinement in the DIALS framework. Acta Crystallogr. D Struct. Biol. *72*, 558–575.

Webb, B., and Sali, A. (2016). Comparative protein structure modeling using MODELLER. Curr. Protoc. Protein Sci. *2016*, 2.9.1–2.9.37.

van Weering, D.H., Moen, T.C., Braakman, I., Baas, P.D., and Bos, J.L. (1998). Expression of the receptor tyrosine kinase Ret on the plasma membrane is dependent on calcium. J. Biol. Chem. *273*, 12077–12081.

Winn, M.D., Ballard, C.C., Cowtan, K.D., Dodson, E.J., Emsley, P., Evans, P.R., Keegan, R.M., Krissinel, E.B., Leslie, A.G.W., McCoy, A., et al. (2011). Overview of the CCP4 suite and current developments. Acta Crystallogr. D Biol. Crystallogr. *67*, 235–242.

Winter, G., Waterman, D.G., Parkhurst, J.M., Brewster, A.S., Gildea, R.J., Gerstel, M., Fuentes-Montero, L., Vollmar, M., Michels-Clark, T., Young, I.D., et al. (2018). DIALS: implementation and evaluation of a new integration package. Acta Crystallogr. D Struct. Biol. *74*, 85–97.

Yang, L., Chang, C.C., Sun, Z., Madsen, D., Zhu, H., Padkjær, S.B., Wu, X., Huang, T., Hultman, K., Paulsen, S.J., et al. (2017). GFRAL is the receptor for GDF15 and is required for the anti-obesity effects of the ligand. Nat. Med. *23*, 1158–1166.

Zhang, K. (2016). Gctf: real-time CTF determination and correction. J. Struct. Biol. *193*, 1–12.

Zheng, S.Q., Palovcak, E., Armache, J.P., Verba, K.A., Cheng, Y., and Agard, D.A. (2017). MotionCor2: anisotropic correction of beam-induced motion for improved cryo-electron microscopy. Nat. Methods *14*, 331–332.

Zivanov, J., Nakane, T., Forsberg, B.O., Kimanius, D., Hagen, W.J.H., Lindahl, E., and Scheres, S.H.W. (2018). New tools for automated high-resolution cryo-EM structure determination in RELION-3. eLife *7*, e42166.

STAR★METHODS

KEY RESOURCES TABLE

REAGENT or RESOURCE	SOURCE	IDENTIFIER
**Chemicals, peptides, and recombinant proteins**		
Gibco ™ Sf-900-III SFM	Thermo Fisher	Cat# 12658019
Lactalbumin	Sigma Aldrich	Cat# 58901C-100ML
Yeastolate	Sigma Aldrich	Cat# 58902C
Ex-Cell420 media	Sigma Aldrich	Cat# 14420C-1000ML
Fetal Bovine Serum	Thermo Fisher	Cat# 10082147
ExpreS^2-Insect TR	Expression Systems	Cat# 95-055-075
Gibco ™ FreeStyle™ 293 Expression Medium	Thermo Fisher	Cat# 12338026
Polyethylimine	Polysciences	Cat# 19850
Optimem	Thermo Fisher	Cat# 31985062
FuGENE® HD Transfection Reagent	Promega	Cat# E2311
FlashBAC Gold Baculovirus Expression System	2B Scientific	Cat# GWB-67B0AE
Glutaraldehyde (50 % v/v)	Sigma Aldrich	Cat# 49628
Recombinant protein: zebrafish RET (aa 1-504, ref# A8E7C6)	This paper	N/A
Recombinant protein: zebrafish RET (aa 1-626, ref# A8E7C6)	This paper	N/A
Recombinant protein: zebrafish GFRα1a (aa 1-353, ref# Q98TT9)	This paper	N/A
Recombinant protein: zebrafish GDNF (aa 135-235, ref# Q98TU0)	This paper	N/A
Recombinant protein: human RET (aa 1-635, ref# P07949)	This paper	N/A
Recombinant protein: human GFRAL (aa ref# Q6UXV0)	This paper	N/A
Recombinant protein: human GDF15 (aa ref# Q99988)	This paper	N/A
**Critical commercial assays**		
Monolith Protein Labeling RED-NHS 2nd generation (Amine Reactive)	Nanotemper	Cat# MO-L001
Nanotemper hydrophobic capillaries	Nanotemper	Cat# MO-KO23
**Deposited data**		
zRET^{CLD1-4} crystal structure	This paper	PDB: 7AMK
zGFRα1$^{150-353}$-zGDNF$^{135-235}$ complex crystal structure	This paper	PDB: 7AB8
zRET$^{22-595}$- zGFRα1$^{29-353}$-zGDNF$^{135-235}$ complex cryo-EM structure	This paper	PDB: 7AML
The zRGα1a C2 symmetry / the zRGα1a symmetry expanded cryo-EM maps	This paper	EMD11822
The hR15AL negative stain EM map	This paper	EMD11777
hRET^{CLD1-2} crystal structure	Kjær et al., 2010	PDB: 2X2U
hRET^{ECD}-GDF15^{mat}·GFRAL$^{129-318}$ cryo-EM structure	Li et al., 2019	PDB: 6Q2J
hGDNF-hGFRα1^{D2-D3} crystal structure	Parkash and Goldman (2009)	PDB: 3FUB
hGFRα2-neurturin crystal structure	Sandmark et al., 2018	PDB: 5MR4
hRET^{ECD}-GFRα2-neurturin cryo-EM structure	Li et al., 2019	PDB: 6Q2O
C-cadherin ectodomain crystal structure	Boggon et al., 2002	PDB: 1L3W
N-cadherin EC1 domain crystal structure	Shapiro et al., 1995	PDB: 1NCI
N-cadherin EC1 domain solution structure	Koch et al., 2004	PDB: 1OP4
Protocadherin Beta 1 EC1-3 crystal structure	Rubinstein et al., 2015	PDB: 4ZPL
Protocadherin Alpha C2 EC1-3 crystal structure	Rubinstein et al., 2015	PDB: 4ZPM
Protocadherin Gamma C5 EC1-3 crystal structure	Rubinstein et al., 2015	PDB: 4ZPO
Protocadherin Gamma A8 EC1-3 crystal structure	Rubinstein et al., 2015	PDB: 4ZPS

(*Continued on next page*)

Continued		
REAGENT or RESOURCE	SOURCE	IDENTIFIER
E-cadherin domains 1 and 2	Nagar et al., 1996	PDB: 1EDH
**Experimental models: cell lines**		
Human: Expi293 cells	Thermo Fisher	Cat# A14527
Insect: Sf21 cells	Thermo Fisher	Cat# 11497013
Insect: Hi Five cells	Thermo Fisher	Cat# B85502
Insect: *Drosophila* S2 cells	Thermo Fisher	Cat# R69007
**Recombinant DNA**		
Plasmid: pCEP	Kohfeldt et al., 1997	N/A
Plasmid: pCEP-hGFRAL-Histag	This paper	N/A
Plasmid: pCEP-hGDF15-Histag	This paper	N/A
Plasmid: pExpreS2.1	Expres2ion Biotech	Cat# S2-11A-001
Plasmid: pExpreS2.1-hRET$^{1-635}$-Tev-Avi-Ctag	This paper	N/A
Plasmid: pBacPak	Clontech Laboratories, Inc.	Cat# 631410
Plasmid: pBacPak-Mellitin1-20-zGDNF$^{135-235}$	This paper	N/A
Plasmid: pBacPak-zGFRα1a^{1-35n3}-3C-Tev-ProteinA	This paper	N/A
Plasmid: pBacPak-zRET$^{1-619}$-3C-TEV-ProteinA	This paper	N/A
Plasmid: pBacPak-zRET$^{1-504}$(N259Q, N308Q, N309Q, N433Q) - 3C-TEV-ProteinA	This paper	N/A
**Software and algorithms**		
NanoTemper analysis software	NanoTemper	v1.2.231
GraphPad Prism	GraphPad Software Inc.	http://www.graphpad.com/scientific-software/prism/
DIALS	Waterman et al., 2016, Winter et al., 2018	https://dials.github.io/
iMosflm & SCALA	Winn et al., 2011	www.ccp4.ac.uk/
PHASER	McCoy et al., 2007	http://www.phaser.cimr.cam.ac.uk/
PHENIX	Adams et al., 2010	http://www.phenix-online.org/
Coot	Emsley et al., 2010	http://www2.mrc-lmb.cam.ac.uk/Personal/pemsley/coot/
RELION	Scheres, 2012, Kimanius et al., 2016, Zivanov et al., 2018	https://github.com/3dem/relion
Scipion	de la Rosa-Trevín et al., 2016	http://scipion.i2pc.es/
Xmipp	de la Rosa-Trevín et al., 2013	http://scipion.i2pc.es/
Gautomatch	K. Zhang, MRC LMB	https://www.lmb.cam.ac.uk/kzhang/
CryoSparc	Structa Biotechnology Inc. Punjani et al., 2017	https://cryosparc.com/
3DFSC	Tan et al., 2017	https://3dfsc.salk.edu/, https://github.com/LyumkisLab/3DFSC
PyMOL	DeLano Scientific LLC	http://www.pymol.org/
Chimera	Pettersen et al., 2004	https://www.cgl.ucsf.edu/chimera/
Proteome Discoverer v.2.3 with XlinkX node	Thermo Fisher, Liu et al., 2015, Kao et al., 2011	Cat# OPTON-30795/30799
xiNET	Combe et al., 2015	Crosslinkviewer.org

RESOURCE AVAILABILITY

Lead contact

Further information and requests for resources and reagents should be directed to and will be fulfilled by the Lead Contact N.Q.M (neil.mcdonald@crick.ac.uk).

Materials availability

The study did not generate new unique reagents.

Data and code availability

The coordinates for the zRET-CLD(1-4), zGDNF-zGFRα1a and zRGα1a are available in the PDB with the primary accession code 7AMK, 7AB8 and 7AML, respectively. The zRGα1a C2 symmetry applied map, the zRGα1a symmetry expanded map and the hR15AL negative stain envelopes are available on the EMDB with accession codes EMD-11822 and EMD-11777, respectively.

EXPERIMENTAL MODEL AND SUBJECT DETAILS

Expi293 cells were used in this study and were grown in suspension in Freestyle 293 Expression media. SF21 and Hi Five insect cells were also used in this study and were grown in serum-free media. Finally Drosophila S2 cells were used in this study and were grown in Ex-Cell420 medium. Additional details are provided in the Method Details section.

METHOD DETAILS

Zebrafish RET CLD(1-4) expression and purification

Zebrafish RET$^{1-504}$ (zCLD(1-4)$^{red.sug.}$) was designed with glycosylation site mutations N259Q, N308Q, N390Q and N433Q to aid in crystallisation. This construct was cloned into a pBacPAK-LL-vector together with a 3C-cleavable C-terminal Protein A tag. A recombinant baculovirus was prepared using the FlashBAC system (2B Scientific). For protein production, SF21 cells were grown to a cell density of 1×10^6 and incubated with recombinant virus for 112 hours at 27°C. The media was harvested and incubated with IgG sepharose (Sigma), with 1 ml of resin slurry to 1 l of media, whilst rolling at 4°C for 18 hrs. The resin was recovered and washed with 5 column volumes (c.v.) of 20 mM Tris (pH 7.5), 200 mM NaCl, 1 mM $CaCl_2$ then incubated with 1:50 (w/w) PreScission Protease (GE Healthcare) for 18 hrs at 4°C. The eluted zCLD(1-4)$^{red.sug.}$ was further purified using a SuperDex 200 (GE Healthcare).

zCLD(1-4)$^{red.sug.}$ crystallisation and X-ray data collection

The purified zCLD(1-4)$^{red.sug.}$ was concentrated to 12 mg/ml. Vapour diffusion drops were set up with 2 μl of protein and 2 μl of precipitant; 50 mM MES (pH 6.2), 31.5 % PEG MME 350 (v/v), against 90 μl of precipitant. After 24 hrs of equilibration seeding was performed using Crystal probe (Hampton Scientific). Crystals grew over 14 days at which point they were harvested and flash frozen in liquid nitrogen.

zCLD(1-4)$^{red.sug.}$ x-ray data processing and structure determination

Data from these crystals was collected at the Diamond Light Source, initially on beamline I04 and finally on beamline I03. The data was processed with XIA2 utilising DIALS (Winter et al., 2018), before further processing through STARANISO (Tickle et al., 2018) for anisotropy correction to give a 2.08 Å dataset (cut to 2.20 Å for refinement owing to low completeness in the outer shells). Crystals belonged to the triclinic space group P1 with cell dimensions shown in Table 1. Molecular replacement was used as implemented in PHASER (McCoy et al., 2007) to initially locate two copies of CLD1-2 (PDB code 2X2U). The positions of the two associated copies of CLD4 were then determined, utilising an ensemble of the following seven models (superposed by secondary structure matching in COOT): 1L3W (resid A 6-99)(Boggon et al., 2002), 1NCI (resid A 6-99)(Shapiro et al., 1995), 1OP4 (resid A 40-123)(Koch et al., 2004), 4ZPL (resid A 206-314)(Rubinstein et al., 2015), 4ZPM (resid B 207-317)(Rubinstein et al., 2015), 4ZPO (resid A 205-311)(Rubinstein et al., 2015) and 4ZPS (resid A 205-313)(Rubinstein et al., 2015). Initial refinement with PHENIX.REFINE was followed by automated model building with PHENIX.AUTOBUILD (Terwilliger et al., 2007) which completed most of the two polypeptide chains present. Cycles of manual model building with COOT and refinement with PHENIX.REFINE (Afonine et al., 2012) followed. Insect cell glycosylation sites were modelled and checked using PRIVATEER (Agirre et al., 2015), with additional libraries, describing the linkages between monomers generated, and used initially in refinement to maintain a reasonable geometry.

zGDNF$^{mat.}$-zGFRα1a^{D1-3} expression and purification

Baculoviruses for zebrafish GFRα1a$^{1-353}$ (zGFRα1a^{D1-3}) and zebrafish GDNF$^{135-235}$ (zGDNF$^{mat.}$) were produced using the pBacPAK-LL-zGFRα1a^{D1-3}-3C-ProteinA construct and the pBacPAK-LL-melittin$^{1-20}$-zGDNF$^{mat.}$-3C-ProteinA respectively and FlashBacGold viral DNA (2B Scientific) using standard protocols (2B Scientific). Recombinant baculoviruses producing either zGDNF$^{mat.}$ or zGFRα1^{D1-3} were used with SF21 insect cells. The protein was expressed one of two methods. (1) 6 x 2 L flasks containing 500 ml of SF21 cells grown to a cell density of 1 x 10^6 in SFIII media (Gibco, ThermoFisher), were each infected with 10 ml of the zGDNF$_{mat.}$ baculovirus stock and 2 ml of the zGFRα1^{D1-3} baculovirus stock for 86 hrs at 27°C. (2) 4 x 2L flasks containing 300 ml of SF21 cells grown to a cell density of 5 x 10^6 in SFIII media, were each infected with 30 ml of the zGDNF$^{mat.}$ baculovirus stock and 6 ml of the zGFRα1^{D1-3} baculovirus stock, with 12 ml of yeastolate (50 x stock, Sigma Aldrich), 12 ml lactalbumin (50 x stock, Sigma-Aldrich) and 6 ml glucose (5 M) for 86 hrs at 27°C. Cells were pelleted at 3500 xg and the media containing the secreted 2:2 zGFRα1a^{D1-3}-zGDNF$^{mat.}$ complex was pooled. A 1 ml slurry of IgG sepharose resin (GE Healthcare) was added to 1 l of media and incubated at 4°C for 18 hrs. The resin was recovered and washed with 5 column volumes of 20 mM Tris (pH 7.0), 150 mM NaCl and 1 mM $CaCl_2$, resuspended in 2 column volumes of the same buffer and incubated with GST-3C (20 μl at 8 mg/ml) for 16 hours.

zGDNF$^{mat.}$-zGFRα1^{D1-3} was further purified using size exclusion chromatography using a Superdex 200 (16/600) (GE Healthcare) in 20 mM Tris (pH 7.0), 100 mM NaCl and 1 mM $CaCl_2$.

zGDNF$^{mat.}$-zGFRα1a^{D1-3} crystallisation and structure determination

Purified zGDNF$^{mat.}$-zGFRα1^{D1-3} was concentrated to 2.5 mg/ml. 100 nl of protein was dispensed with 100 nl of precipitant onto sitting well trays (MRC-2 drop trays) which comprised 100 mM Tris (pH 8.0), 5 % (w/v) PEG 20,000, 3.7 % (v/v) acetonitrile and 100 mM NaCl. A volume of 90 μl of precipitant solution was dispensed into the well and the trays were then incubated at 22°C. Crystals of zGDNF$^{mat.}$-zGFRα1^{D1-3} formed after 30 days. Crystals were harvested after 55 days and frozen in liquid N_2 with 30 % (v/v) ethylene glycol used as a cryo-protectant. Data was collected on I04 at Diamond using PILATUS 6M Prosport+ detector. The X-ray diffraction data collected was reduced and integrated using DIALS (Waterman et al., 2016; Winter et al., 2018) at the Diamond Light Source. The structure was phased by molecular replacement in PHASER (McCoy et al., 2007) and in CCP4 (The CCP4 Suite, 1994; Winn et al., 2011) using the human GDNF-GFRα1 starting model (PDB 3FUB) (Parkash & Goldman (2009)). Model refinement was performed using COOT (Emsley and Cowtan, 2004; Emsley et al., 2010) and PHENIX.REFINE (Adams et al., 2010; Afonine et al., 2012) against the dataset that was reduced and integrated using the STARANISO (Tickle et al., 2018) at a resolution of 2.2 Å. Glycosylation sites were validated using PRIVATEER (Agirre et al., 2015).

zRET^{ECD}-zGDNF$^{mat.}$-zGFRα1a^{D1-3}- (zRGα1a) complex expression and purification

A recombinant baculovirus was prepared to produce zRET^{ECD} (residues 1-626) using the pBacPAK-LL-zRET^{ECD}-3C-Protein A construct and FlashBac viral DNA (2B Scientific) using standard protocols and as described above. To produce zRET^{ECD} either one of two separate protocols were used; (1) SF21 insect cells grown using SFIII media in 6×500 ml flasks to a cell density of 1×10^6 were then infected with 2 ml of the baculovirus that contained zRET^{ECD} for 86 hrs at 27°C, (2) 4 x 2L flasks containing 300 ml of SF21 cells grown to a cell density of 5.5 x 10^6 in SFIII media, were each infected with 6 ml of the zRET^{ECD} baculovirus stock, with 12 ml of yeastolate (50 x stock, Sigma-Aldrich), 12 ml lactalbumin (50 x stock, Sigma-Aldrich) and 6 ml glucose (5 M) for 86 hrs at 27°C. Cells were pelleted at 3500 g and the media containing secreted zRET^{ECD} was pooled and 1 ml of IgG sepharose resin (GE Healthcare) was added to 1 l of media and incubated at 4°C for 18 hrs. The resin was recovered and washed with 5 column volumes of 20 mM Tris (pH 7.0), 150 mM NaCl and 1 mM $CaCl_2$, then resuspended in 2 column volumes of the same buffer. Purified 2:2 zGFRα1a^{D1-3}-zGDNF$^{mat.}$ complex was then added directly. The sample was incubated for 45 min at 4°C. The resin with the zRGα1a complex was then recovered and washed with 5 c.v. of 20 mM Tris (pH 7.0), 150 mM NaCl and 1 mM $CaCl_2$ buffer, resuspended in 2 column volumes of buffer and incubated with GST-3C (20 μl at 8 mg/ml) for 18 hours at 4°C. The eluted zRGα1a complex was further purified using size exclusion chromatography using a Superdex 200 (16/600) (GE Healthcare) in 20 mM HEPES (pH 7.0), 150 mM, NaCl and 1 mM $CaCl_2$.

To prepare a cross-linked sample, 100 μl of purified zRGα1a (4 mg/ml) was applied on top of a 5-20 % (w/v) sucrose gradient which contained a 0-0.1 % (v/v) glutaraldehyde gradient, the gradient was buffered with 20 mM HEPES (pH 7.0), 150 mM NaCl and 1 mM $CaCl_2$. Ultracentrifugation was performed at 33,000 r.p.m (SW55 rotor) for 16 hours at 4°C. The sucrose gradient was fractionated in 125 μl fractions, the glutaraldehyde was quenched with 1 M Tris (pH 7.0), to a final concentration 100 mM. The fractions that contained cross-linked zRGα1a were pooled and further purified by Superdex200inc 10/300 (GE Healthcare) in a buffer of 20 mM Tris (pH 7.0), 150 mM NaCl and 1 mM $CaCl_2$, in order to remove the sucrose from the crosslinked zRGα1a complex.

zRGα1a cryo-electron microscopy sample preparation

To prepare cryo-EM grids, 1.2/1.3 300 mesh Cu Quantifoil™ grids 300 mesh grids were glow discharged using 45 mA for 30 s using a Quorum Emitech K100X. For the untilted dataset (Dataset 1), 4 μl of crosslinked zRGα1a sample, at 0.1 mg/ml, was applied to the grids, using a Vitrobot Mark IV (Thermo Fisher) with the parameters; 90 s wait time, 5 s blot time at 22°C with 100 % humidity. The same glow discharge parameters were used for the grids for the tilted dataset (dataset 2), 4 ul was applied to the grid at 4°C and a 20 s wait with 3 s blot time under 100 % humidity. For the non-crosslinked zRGα1a sample, the same glow discharge parameters were used for 1.2/1.3 300 mesh Cu Quantifoil™ grids 300 mesh grids. 4 μl of non-crosslinked zRGα1a at 0.1 mg/ml was applied to the grids with the same parameters as those used for the grids prepared for dataset 1, these grids were used for dataset 3.

Cryo-EM data acquisition: Datasets 1 to 3

Frozen-hydrated grids of the crosslinked zRGα1a sample were imaged on a Titan Krios electron microscope (Thermo Fisher) operating at 300 kV at the Francis Crick Institute. Movies were captured on a BioQuantum K2 detector (Gatan) in counting mode at 1.08 Å/pixel and with an energy filter slit width of 20 eV. Dataset 1 was collected with a 0° tilt angle, a defocus range of 1.4-3.5 μm and comprised a total of 6105 movies. For dataset 2, 6375 movies were collected in total using a tilt angle of 30° and the same defocus range used for dataset 1. Movies from datasets 1 and 2 had an exposure of 1.62 $e^-/Å^2$ per frame for a total electron exposure of 48.6 $e^-/Å^2$. The dose rate was 6.4 e^-/pixel/sec and exposure time was 9 seconds/movie. For dataset 3, frozen-hydrated grids of non-crosslinked zRGα1a were collected on a Talos Arctica microscope (Thermo Fisher) operating at 200 kV at the Francis Crick Institute. A total of 1705 movies were captured on a Falcon 3 detector in integrating mode at 1.26 Å/pix and a defocus range of 1.5-3.0 μm. Movies from dataset 3 had an exposure of 6.07 $e^-/Å^2$ per frame which led to a total exposure of 60.66 $e^-/Å^2$. All datasets were collected using EPU version 1.9.0 (Thermo Fisher).

Cryo-EM data processing of crosslinked zRGα1a (dataset 1)

MotionCorr2 (Zheng et al., 2017) was used to correct for motion in the movie frames in Scipion 1.2 (de la Rosa-Trevín et al., 2016). The contrast transfer function was estimated using CTFfind4.1(Rohou and Grigorieff, 2015). 5855 micrographs were selected from dataset 1 and initial particle picking was performed with RELION-2.1 manual picking, 4899 particles were extracted with RELION-2.1 (Kimanius et al., 2016) particle extract function (de la Rosa-Trevín et al., 2016) with a box size of 340 and binned two-fold. 2D classification was performed using RELION 2D classification, with 20 initial classes. Six classes were used to pick a subset of 3000 micrographs using RELION-2.1 autopicking in Scipion 1.2, giving 638,000 particles with box size 340, binned 2 fold. These were classified using 2D classification in RELION-2.1. Twelve classes were selected for picking using Gautomatch [K. Zhang, MRC LMB (www.mrc-lmb.cam.ac.uk/kzhang/)] to pick 2,424,600 particles, which were extracted with a box size of 340 pixels and binned 2-fold using RELION-2.1 2D class averaging was performed in CryoSPARC-2 (Punjani et al., 2017) leading to 1,156,517 particles which were extracted using RELION-2.1 (Kimanius et al., 2016; Scheres, 2012) with a box size of 320 pixels.

Cryo-EM data processing of crosslinked zRGα1a (tilted dataset 2)

Dataset 2 was processed and corrected for motion correction and CTF estimation as described above. A total of 4848 micrographs were used to pick particles semi-automatically with Xmipp and 69,386 particles were extracted with a box size of 360 pixels using RELION-2.1 (Kimanius et al., 2016; Scheres, 2012) in Scipion1.2 (de la Rosa-Trevín et al., 2016). 2D classification was then performed using RELION automatic picking leading to 1,183,686 particles being extracted using RELION-2.1 (Kimanius et al., 2016; Scheres, 2012; Zivanov et al., 2018) with a box size of 340 binned 2-fold. Subsequent 2D classification in RELION-2.1 (Kimanius et al., 2016; Scheres, 2012) lead to 12 classes which were used by Gautomatch [K. Zhang, MRC LMB (www.mrc-lmb.cam.ac.uk/kzhang/)] to pick 1,393,023 particles. The particles were extracted with RELION-2.1 (Kimanius et al., 2016; Scheres, 2012) with a box size 320, 2-fold binned, were imported into CryoSPARC-2 (Punjani et al., 2017) and 2D classification generated 208,057 particles from 3175 micrographs. These particles were re-extracted with a box size of 320 and per-particle CTF estimation was performed using GCTF (Zhang, 2016).

Combining and processing cryo-EM datasets 1 and 2 for crosslinked zRGα1a

Dataset 1 and 2 were combined and an initial 2D classification was performed in CryoSPARC-2 on the 1,364,574 particles (Afonine et al., 2018). Following this, 1,242,546 particles underwent two heterogeneous refinements using 5 classes with strict C2 symmetry applied in CryoSPARC-2 (Punjani et al., 2017) lead to a homogeneous refinement with 468,922 particles. Once re-imported into Scipion1.2, RELION 2D class averaging was implemented to generate 364,158 and 22,358 particles from dataset 1 and dataset 2, respectively (Kimanius et al., 2016; Scheres, 2012). Particle polishing was performed in RELION-2.1 (Kimanius et al., 2016). Once imported into CryoSPARC-2, 2D class averaging removed any further particles, yielding 382,547 particles used for a homogeneous refinement followed by a non-uniform refinement with C2 symmetry applied. This final reconstruction gave a resolution of 3.3 Å as calculated using the 'gold' standard (FSC=0.143) (Punjani et al. 2017). Symmetry expansion was performed in RELION-2.1 and 3D-refinement with masking was performed with no symmetry applied (Kimanius et al., 2016; Scheres, 2012). Postprocessing in RELION-2.1 of the final symmetry expanded reconstruction with a resolution 3.5 Å (Figure S4) (Kimanius et al., 2016; Scheres, 2012).

Building the zRGα1a complex into the final cryo-EM map

To build a full ligand-co-receptor complex, the zGDNF$^{mat.}$-zGFRα1$^{\Delta D1}$ crystal structure described here was used together with a homology model of domain D1 (zGFRα1$^{29-121}$) generated by MODELLER from the GFRα2-neurturin crystal structure (PDB 5MR4) (Sandmark et al., 2018; Webb and Sali, 2016). For zRET, chain A of the CLD(1-4) module described here was used together with a CRD model generated with SwissPROT (Schwede et al., 2003) using the structure of hRET^{ECD} in complex with GFRα2-neurturin (PDB 6Q2O)(Li et al., 2019; Webb and Sali, 2016). The zGDNF-zGFRα1 and zRET^{ECD} structures were then docked into the symmetry expanded map using PHENIX (Adams et al., 2010). The model was refined at 4.2 Å against the sharpened map using PHENIX_REAL_SPACE_REFINE (Afonine et al., 2018) and manual model building and model refinement was done in COOT (Emsley and Cowtan, 2004; Emsley et al., 2010). The final symmetry expanded model was used to generate the 2:2:2 zRGα1a model, which was placed in the C2 averaged map using PHENIX (Adams et al., 2010) using PHENIX_REAL_SPACE_REFINE (Afonine et al., 2018). Glycosylation sites were validated using PRIVATEER (Agirre et al., 2015). Protein-protein interface areas were calculated using PDBePISA (Krissinel and Henrick, 2007). All images of maps were produced in Chimera (Pettersen et al., 2004) and structure-based figures were rendered in PyMOL (Schrodinger, 2015).

Cryo-EM data processing for a non-crosslinked zRGα1a sample (dataset 3)

MotionCorr2 (Zheng et al., 2017) was used to correct for motion in the movie frames in RELION-3 (Zivanov et al., 2018). The contrast transfer function was estimated using CTFfind4.1 (Rohou and Grigorieff, 2015). 384 micrographs were selected from and initial particle picking was performed with RELION-3 manual picking, 951 particles were extracted with RELION-3 (Zivanov et al., 2018) particles extract with a box size of 320 and binned 2 fold. 2D classification was performed using RELION-3 2D classification, with 10 initial classes (Zivanov et al., 2018). One class, due to the orientation bias, was selected and used by RELION autopick to pick from a subset of 81 micrographs. This gave 19,715 particles picked and extracted with a box size of 320 pixels using RELION-3. These particles were sorted in RELION-3 and 15,519 were then were classified using RELION 2D classification. A total of 11070 particles were used from 81 micrographs to explore the linear particle arrays observed for the zRGα1a complex.

For the 2D classification of the isolated zRGα1a particle pairs initial picking performed with RELION-3 manual picking yielded 239 particles that were extracted with a box size of 420 pixels. 2D classification was performed and one class was used to for RELION autopick, yielding 4567 particles that were extracted using RELION extract with a box size of 400 pixels from 81 micrographs. RELION 2D classification produced the final 2D of the isolated zRGα1a pair with 1194 particle pairs (2388 individual particles) (Zivanov et al., 2018).

Analysis of zRGα1a multimer formation on cryo-EM grids

Following 2D class averaging in RELION-3, the final 11070 particles were repositioned onto 81 micrographs collected from cryo-grids prepared from the non-crosslinked zRGα1a sample using RELION particle reposition. A Python script was written to extract the particle number, psi angle (ψ) and Cartesian coordinates of particle pairs from the 2D class average STAR file. Particle pairs were detected through analysing each single particle and locating surrounding particles within 214.2 Å (170 pixels), using their extracted Cartesian coordinates. A subset of 14 micrographs was used, where a total of 3756 individual particles lead to 4132 particle pairs. The distance between each particle pair was determined using their X and Y coordinates. The ψ angles were corrected to positive integers, and were permitted to be within the 180° range due to the C2 symmetry of the complex. The difference between the two positive ψ angles from the particle pairs ($\Delta\psi$) was calculated as an absolute value. Distance between the particles and the $\Delta\psi$ between particle pairs was calculated and plotted on a 3D surface plot with the bins every 2 Å and every 2.6°, respectively.

Human RET^{ECD} expression and purification

A codon-optimised human RET^{ECD} ($hRET^{ECD}$) cDNA encoding residues 1-635 followed by a TEV-cleavable Avi and C-tag was cloned into a pExpreS2.1 vector (ExpreS2ion Biotechnologies, Hørsholm, Denmark) with Zeocin resistance. A stable pool of S2 cells, secreting $hRET^{ECD}$, was generated by transfecting 25 ml of S2 cells grown in Ex-Cell420 medium (Sigma) with 10 % (v/v) FBS at a density of 5×10^6 cells/ml using 12.5 μg of DNA and 50 μl of $ExpresS^2$-Insect TR (5×). Stably transfected cells were selected with 2 mg/ml Zeocin with repeated medium exchange. The culture was expanded to 1 litre in a 5L glass-flask and the supernatants collected after 7 days.

For purification, 1 ml of C-tag capture resin (ThermoFisher) was added to a cleared and filtered S2 supernatant and incubated for 18 hrs at 4°C. The resin was pelleted and washed several times with PBS before eluting bound $hRET^{ECD}$ by competition with PBS containing 200 μg/ml SEPEA peptide. At this point, the affinity and biotinylation tags were removed by digestion with TEV (a 1:10 ratio of TEV protease:RET). The purified $hRET^{ECD}$ was further purified by size-exclusion using a Superdex200 10/300 with a 50 mM Tris (pH 7.5), 100 mM NaCl buffer.

Human $GDF15^{mat.}$-$GFRAL^{D1-3}$ complex expression and purification

Both human $GFRAL^{21-352}$ (referred to hereafter as $hGFRAL^{D1-3}$) and $hGDF15^{198-308}$ (referred to hereafter as $hGDF15^{mat.}$) were cloned into a pCEP vector with an N-terminal BM40 secretion sequence. The hGFRAL construct had a C-terminal 6 His tag. The constructs were co-transfected into Expi293 cells (Life Tech) using polyethylimine. The transfected cells were incubated in Freestyle media at 37°C, 8 % CO_2 with 125 rpm shaking. Conditioned media was harvested after 5 days, and Tris pH 8.0 and imidazole added to a final concentration of 10 mM and 20 mM respectively. The media was incubated with Ni-NTA agarose beads whilst rolling at 4°C for 2 hours. The beads were recovered and washed with 20 mM Tris (pH 7.4), 137 mM NaCl and the protein was eluted with 20 mM HEPES (pH 7.4), 137 mM NaCl and 500mM imidazole. The protein was concentrated to ~5 mg/ml. This protein was further purified by Superdex 200 increase size exclusion chromatography in buffer 20 mM HEPES (pH 7.4), 137 mM NaCl to give a pure 2:2 GDF15-GFRAL complex.

$hRET^{ECD}$-$hGDF15^{mat.}$-$hGFRAL^{D1-3}$ (hR15AL) complex assembly and purification

An excess of purified $hRET^{ECD}$ (300 μl, 1.1 mg/ml) was incubated with purified hGDF15-hGFRAL (300 μl, 0.75 mg/ml) for 1 hr whilst mixing at 4°C in the presence of 10-fold excess heparan sulfate DP-10 (20 μM) (Iduron, UK). The hR15AL complex was further purified by size exclusion chromatography using a Superdex 200 increase in to 20 mM HEPES (pH 7.0), 150 mM NaCl and 1 mM $CaCl_2$. For sample crosslinking, 100 μl of the hR15AL complex (0.75 mg/ml) was applied on top of a 5-20 % (w/v) sucrose gradient which contained a 0-0.1 % (v/v) glutaraldehyde gradient, the gradient was buffered with 20mM HEPES (pH 7.0), 150 mM NaCl and 1 mM $CaCl_2$. Ultracentrifugation was performed at 33,000 rpm for 16 hours at 4°C. The sucrose gradient was fractionated in 125 μl fractions, the glutaraldehyde was quenched with 1M Tris (pH 7.0), to a final concentration 100 mM. The fractions were assessed using SDS-PAGE and fractions that contained the complex were used for negative stain.

hR15AL negative stain preparation, data acquisition and processing

Cu 200 mesh carbon coated grids were glow discharged under vacuum using 45 mA for 30 s. A sample of 4 μl of the crosslinked hR15AL undiluted from the GraFix column was applied to the charged grid and left for 30 s and the excess removed by blotting and placing the grid, sample side facing the solution, in 10 μl of 2 % (w/v) uranyl acetate solution in d.H_2O, and blotting immediately twice, followed by placing the grid in the 3rd 10 μl drop sample side facing down and leaving it in solution for 1 min, followed by a final blot until almost all the solution has been wicked off. The grid was then left to dry for 5 mins.

Micrographs were collected on a BMUltrascan 1000 2048x2048 CCD detector using a Tecnai Twin T12 (Thermo Fisher) at 120 kV with a defocus range of 1-1.5 μm and with a 1 s exposure time. A total of 299 micrographs were collected and particles were picked

using Xmipp (de la Rosa-Trevín et al., 2013) semi-automated picking, in Scipion1.2 (de la Rosa-Trevín et al., 2016). This gave 27,551 particles were extracted with RELION-2.0 particle extraction (Kimanius et al., 2016; Scheres, 2012). 2D class averaging was performed with RELION-2.0 (Kimanius et al., 2016; Scheres, 2012). The resulting 16,159 particles were used to generate an initial model using RELION 3D ab-initio model. 3D classifications with 5 classes were performed using RELION-2.0 3D classification (Kimanius et al., 2016; Scheres, 2012). 6519 particles were taken forward into the final reconstruction a resolution of 25.8 Å using RELION-2.0 3D refinement (Kimanius et al., 2016; Scheres, 2012). The data processing was done in Scipion1.2 (de la Rosa-Trevín et al., 2016).

Microscale thermophoresis (MST) measurement of zRET^{ECD} binding affinity

MST measurements were performed at 25°C in 20 mM HEPES (pH 7.0), 150 mM NaCl, 1 mM $CaCl_2$ and 0.05 % (v/v) Tween-20 using a Nanotemper Monolith NT.115 (Nanotemper). To measure the affinity of zGFRα1^{D1-3}-zGDNF$^{mat.}$ towards zRET^{ECD}; zRET^{ECD} was labelled with NHS-RED 2nd generation dye (Amine Reactive) using the labelling kit (Nanotemper). A 1:1 serial dilution of unlabelled zGFRα1^{D1-3}-zGDNF$^{mat.}$ (WT and mutants) was performed. The samples were incubated with the labelled zRET^{ECD}-NHS-RED (50 nM, fluorophore, 83.7 nM zRET^{ECD}) for 10 mins at 22°C. Hydrophobic treated capillaries were filled with the serially diluted samples (Nanotemper). The MST run was performed using a Monolith 1.115 with the LED power and MST both set to 20 %, with a measurement time of 20 sec. To measure the affinity of zGFRα1^{D1-3}-zGDNF$^{mat.}$ towards zRET$^{ECD}_{P291-Q296;AAG}$; zRET$^{ECD}_{P291-Q296;AAG}$ was labelled with NHS-RED 2nd generation dye (Amine Reactive) using the labelling kit (Nanotemper), and the procedure was carried out as above with zRET$^{ECD}_{P291-Q296;AAG}$-NHS-RED (50 nM, fluorophore, 80.7 nM zRET^{ECD}).

Surface conservation analysis and heatmaps for different GFL-GFR ligand-coreceptor pairs

The sequence for the globular domains of zGFRα1a (Uniprot Q98TT9) was aligned to hGFRα1 (Uniprot P56159), hGFRα2 (Uniprot O00451), hGFRα3 (Uniprot O60609), hGFRα4 (Uniprot Q9GZZ7), and hGFRAL (Uniprot Q6UXV0), using Clustal Omega.(Sievers et al., 2011) The sequence of the mature zGDNF (Uniprot Q98TU0) was aligned to hGDNF (Uniprot P39905), hNRTN (Uniprot Q99748), hARTN (Uniprot Q5T4W7), hPSPN (Uniprot O60542), and hGDF15 (Uniprot Q99988) using Clustal Omega (Sievers et al., 2011). Using these alignments, residues were categorised based on residue type and a heat map was generated and values mapped onto a surface representation on the zGFRα1a^{D2-D3}. D1 was excluded from the analysis due to the major differences between each of the co-receptors; which is missing hGFRα4 and is located in a completely different position in hGFRAL. Each of the categories for residue type are as follows; aromatic residues (F, W, and Y), aliphatic residues (A, I, L, and V), residues containing an alcohol functional group (S and T), positively charged residues (R and K), negatively charged residues (D and E), and residues containing an amide bond in the side chain (N and Q), and C, G, H and M were counted individually. The sequence similarity was numbered from 0-1, 0 indicating no similarity at all and 1 indicating the residue type was identical between the GFR or GFL family members respectively. The value for each residue in the sequence were represented as a surface colour coded with the highest residue similarity in red (1) through yellow (0.5) to white (0).

QUANTIFICATION AND STATISTICAL ANALYSIS

Binding kinetics were derived from the MST binding curves using the NanoTemper analysis software version 1.2.231, with each point determined by averaging data obtained between 10 and 15 sec on the MST curve for each capillary. Fractional binding values from an entire concentration range were derived by normalising the values from 0 to 1 in Microsoft Excel, with visual inspection to check these concentrations corresponded to the plateau in MST signal at low and high ligand concentrations. Data for at least three such binding experiments were imported into Graphpad Prism 8.0.0 and, due to the proximity of the apparent binding constant and fluorescently-labelled RET receptor concentration, subjected to a non-linear regression fit using a quadratic equation to determine the K_D.

Structure, Volume *29*

Supplemental Information

A two-site flexible clamp mechanism for RET-GDNF-GFRα1 assembly reveals both conformational adaptation and strict geometric spacing

Sarah E. Adams, Andrew G. Purkiss, Phillip P. Knowles, Andrea Nans, David C. Briggs, Annabel Borg, Christopher P. Earl, Kerry M. Goodman, Agata Nawrotek, Aaron J. Borg, Pauline B. McIntosh, Francesca M. Houghton, Svend Kjær, and Neil Q. McDonald

SUPPLEMENTARY FIGURES

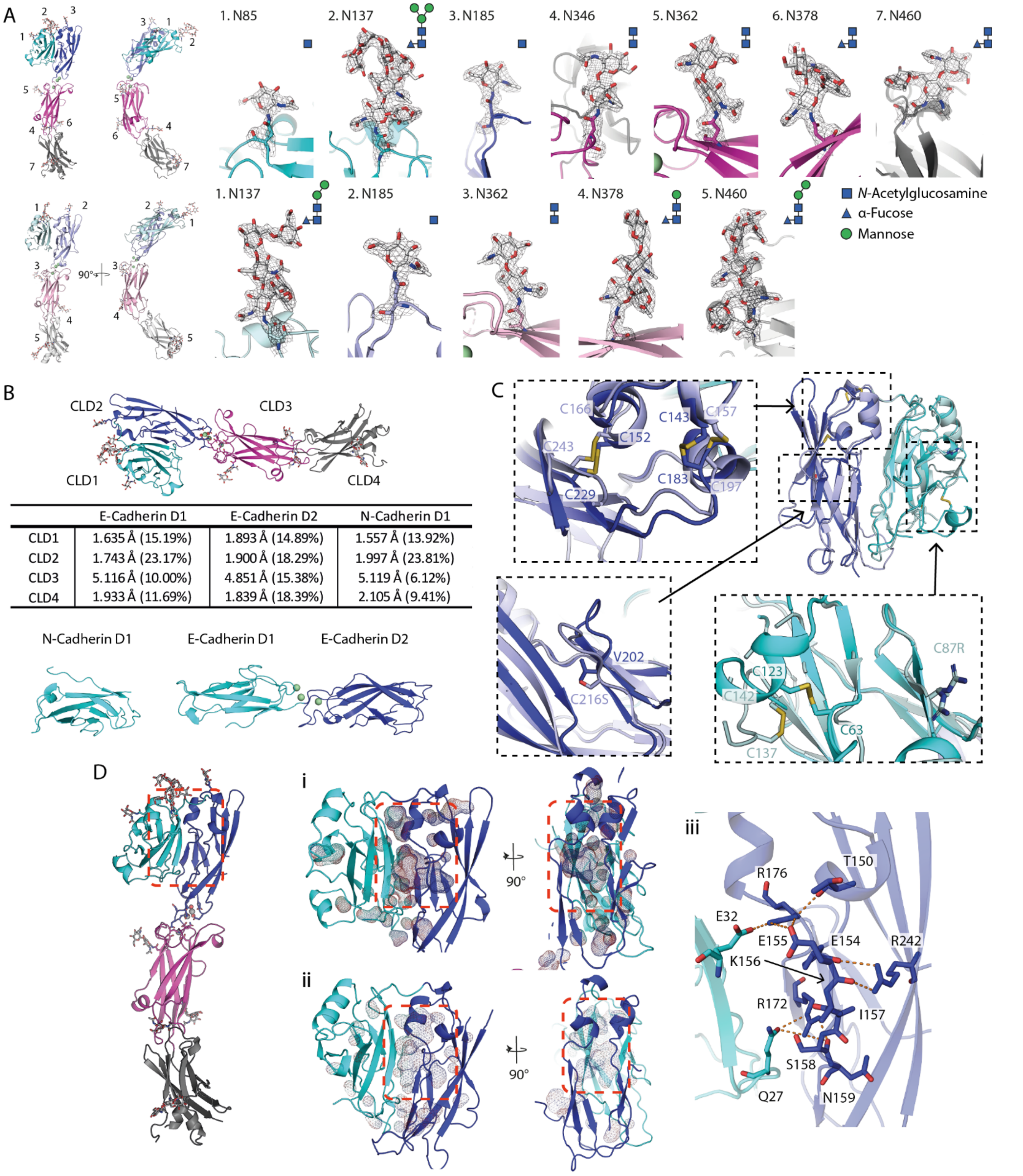

	E-Cadherin D1	E-Cadherin D2	N-Cadherin D1
CLD1	1.635 Å (15.19%)	1.893 Å (14.89%)	1.557 Å (13.92%)
CLD2	1.743 Å (23.17%)	1.900 Å (18.29%)	1.997 Å (23.81%)
CLD3	5.116 Å (10.00%)	4.851 Å (15.38%)	5.119 Å (6.12%)
CLD4	1.933 Å (11.69%)	1.839 Å (18.39%)	2.105 Å (9.41%)

Supplementary Figure 1: Structural analysis of human RET CLD1-4$^{red.Sug.}$ A) Asparagine-linked sites (N-linked) within the zRET^{CLD1-4} module and final electron density map calculated using m2Fo-DFc coefficients contoured at 1.0 σ corresponding to each N-linked glycosylation site are shown. B) Calculated RMSD values (and sequence identity) between each of the cadherin like domains from zCLD1-4$^{red.sug.}$ and E-cadherin domains 1 and 2 (PDB 1EDH)(Nagar et al., 1996) and N-cadherin domain 1 (PDB 1NCI)(Shapiro et al., 1995). C) Disulfide swapping evident between higher and lower vertebrates; zCLD1-2 (in cyan and blue) aligned with the hCLD12 structure (in pale cyan and pale blue, PDB 2X2U), with close-ups of the cysteine within the structure. The two unpaired cysteines unique to hRET were mutated to arginine (C87R) and a serine (C216S) to aid structure determination of hRET^{CLD1-2} (Kjær et al., 2010). D) Analysis of the zCLD1-2 clamshell interface; orthogonal views of the cavity (shown in mesh) within CLD1-2 for both zebrafish (i) and human (ii) (PDB 2X2U)(Kjær et al., 2010). (iii) The residues incorporated into the CLD1-2 clamshell interface; CLD2-β1 stabilised with R242 (CLD2-β6), R172 and R176 (CLD2-β2) the latter of which also interact with Q27 and E32 (CLD1-β1). In all cases the structures are represented as a cartoon and the residues are represented as sticks with individual domains are coloured as follows; CLD1 in cyan, CLD2 in blue, CLD3 in magenta and CLD4 in grey. All images were rendered in PyMOL (Schrodinger, 2015). Related to Figure 1.

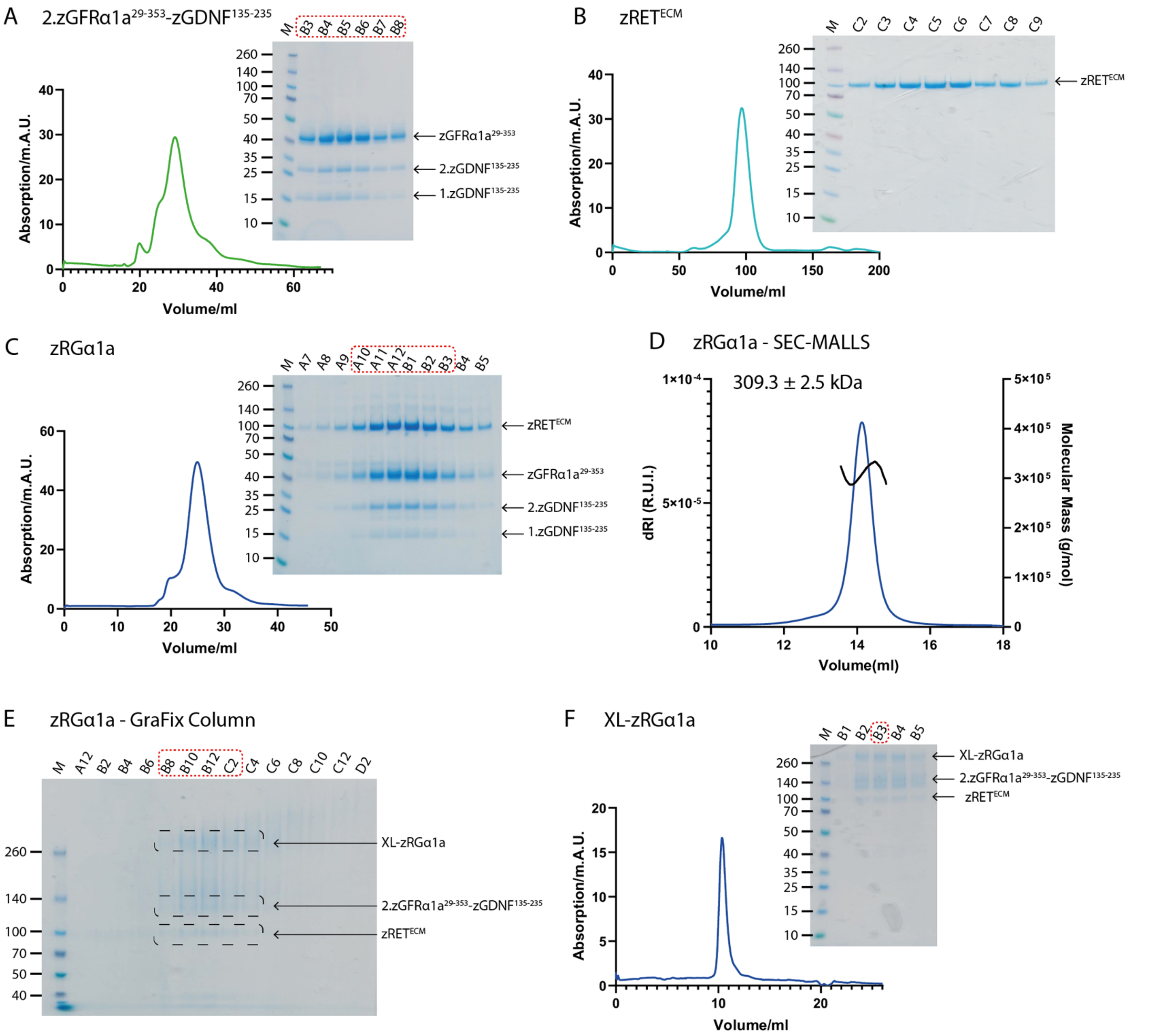

Supplementary Figure 2: Purification of individual zRGα1a components and crosslinking of the zRGα1a ternary complex. Size exclusion profiles of A) 2.zGFRα1a$^{29\text{-}353}$-zGDNF$^{135\text{-}235}$, B) zRET^{ECD} (shown here as zRET^{ECM} for extracellular module) and C) zRGα1a D) SEC-MALLS trace of the purified zRGα1a E) SDS-PAGE of fractionated GraFix stabilised zRGα1a sample. F) Size exclusion profile of the crosslinked zRGα1a sample.Related to STAR methods.

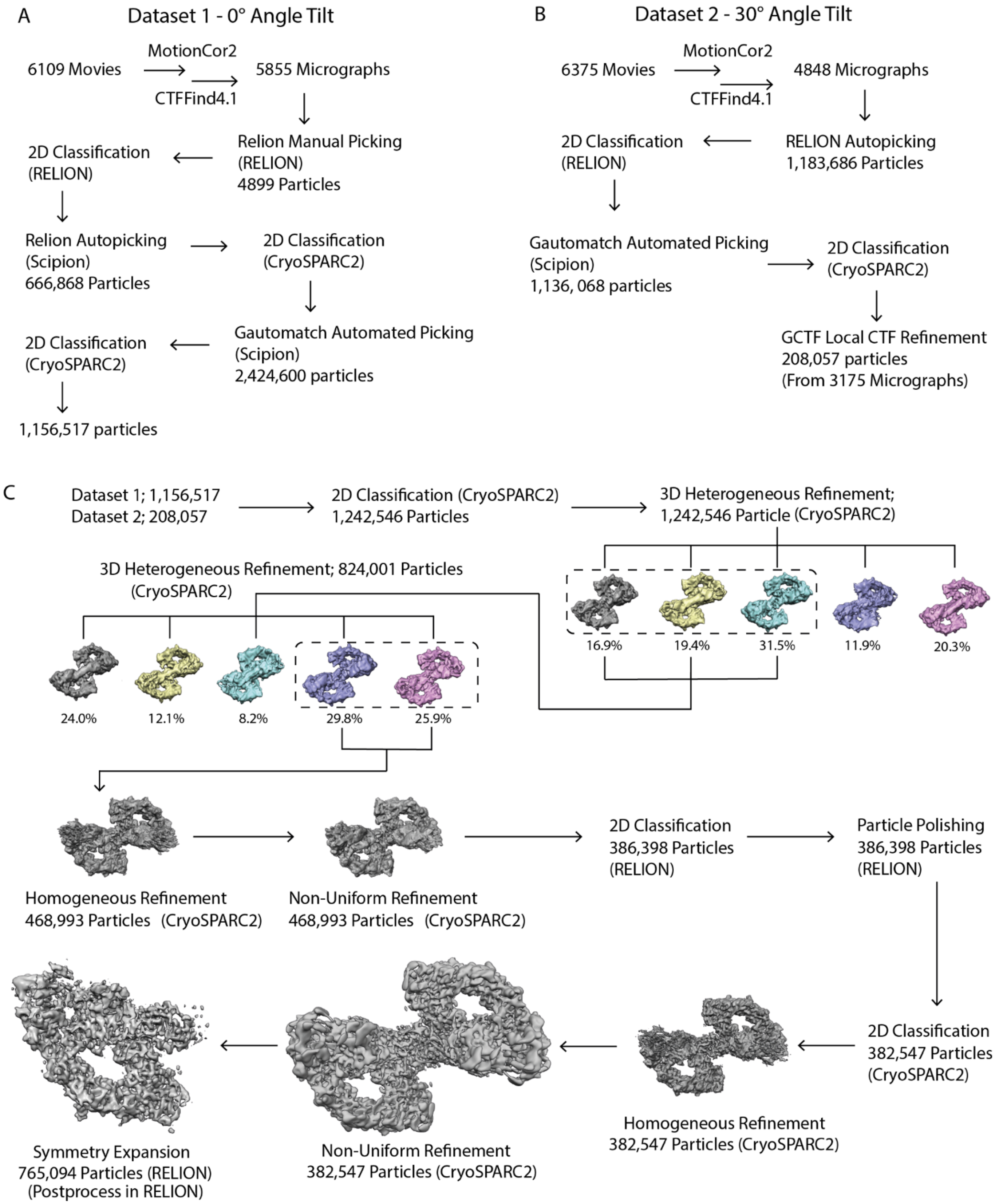

Supplementary Figure 3: zRGα1a cryo-EM data processing workflow. A) Non-tilted particles dataset 1 and B) tilted particles dataset 2 were processed independently. C) Combined particles from both datasets 1 and 2 and workflow. Software packages used; CryoSPARC2 (Punjani et al., 2017), CTFFind4.1 (Rohou and Grigorieff, 2015), Gautomatch [K. Zhang, MRC LMB (www.mrc-lmb.cam.ac.uk/kzhang/)], GCTF (Zhang, 2016), MotionCor2 (Zheng et al., 2017) RELION (Kimanius et al., 2016; Scheres, 2012; Zivanov et al., 2018), Scipion (de la Rosa-Trevín et al., 2016), Related to Figure 2.

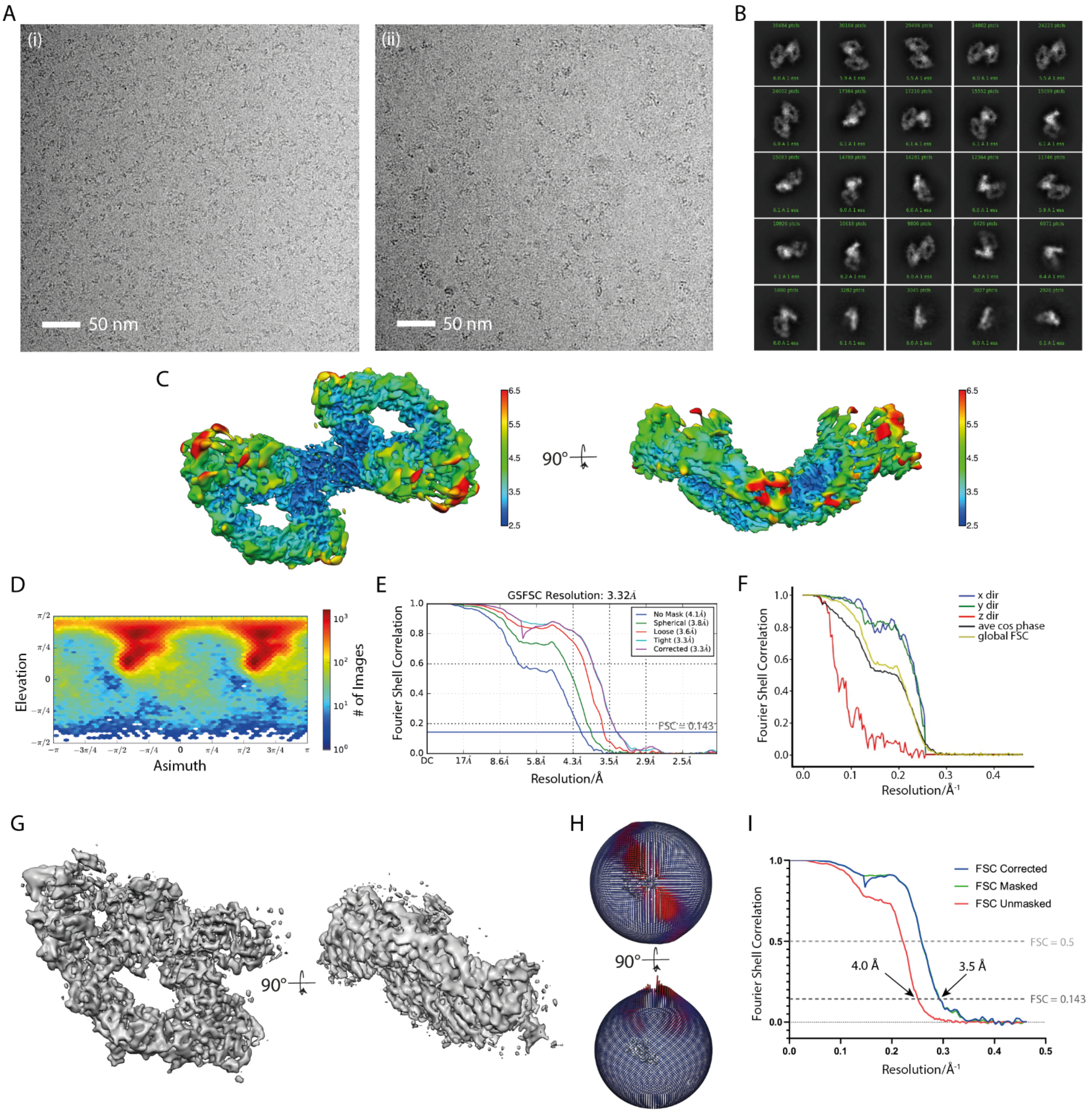

Supplementary Figure 4: zRGα1a single particle cryo-EM reconstruction. A) Representative micrographs from the non-tilted dataset (i) and the dataset collected with a tilt angle of 30° (ii). B) Twenty-five 2D class averages from all the particles in the final reconstruction. C) The C2 averaged cryo-EM map is coloured by local resolution and was generated by the locRes option in CryoSPARC2 (Punjani et al., 2017) using blue for 2.5 Å resolution areas, green for 4.5 Å and red for 6.5 Å resolution. A total of 382,574 particles were generated using CryoSPARC2 (Punjani et al., 2017), non-uniform refinement and postprocessed in RELION (Kimanius et al., 2016; Scheres, 2012; Zivanov et al., 2018). Two orthogonal views of the zRGα1a complex are shown. D) Angular distribution of the particles in the C2 averaged map. E) Fourier shell correlation curve of the C2 averaged map. F) A 3DFSC (Tan et al., 2017) plot generated from the C2 averaged map. G) Two views orthogonal of the symmetry expanded map, generated using particles expansion in RELION (Kimanius et al., 2016; Scheres, 2012; Zivanov et al., 2018). H) Two orthogonal views of the angular distribution from the symmetry expanded map. I) The Fourier shell correlation curves from the symmetry expanded map showing an overall resolution of 3.5 Å. Images of the maps were rendered using Chimera (Pettersen et al., 2004). Related to Figure 2.

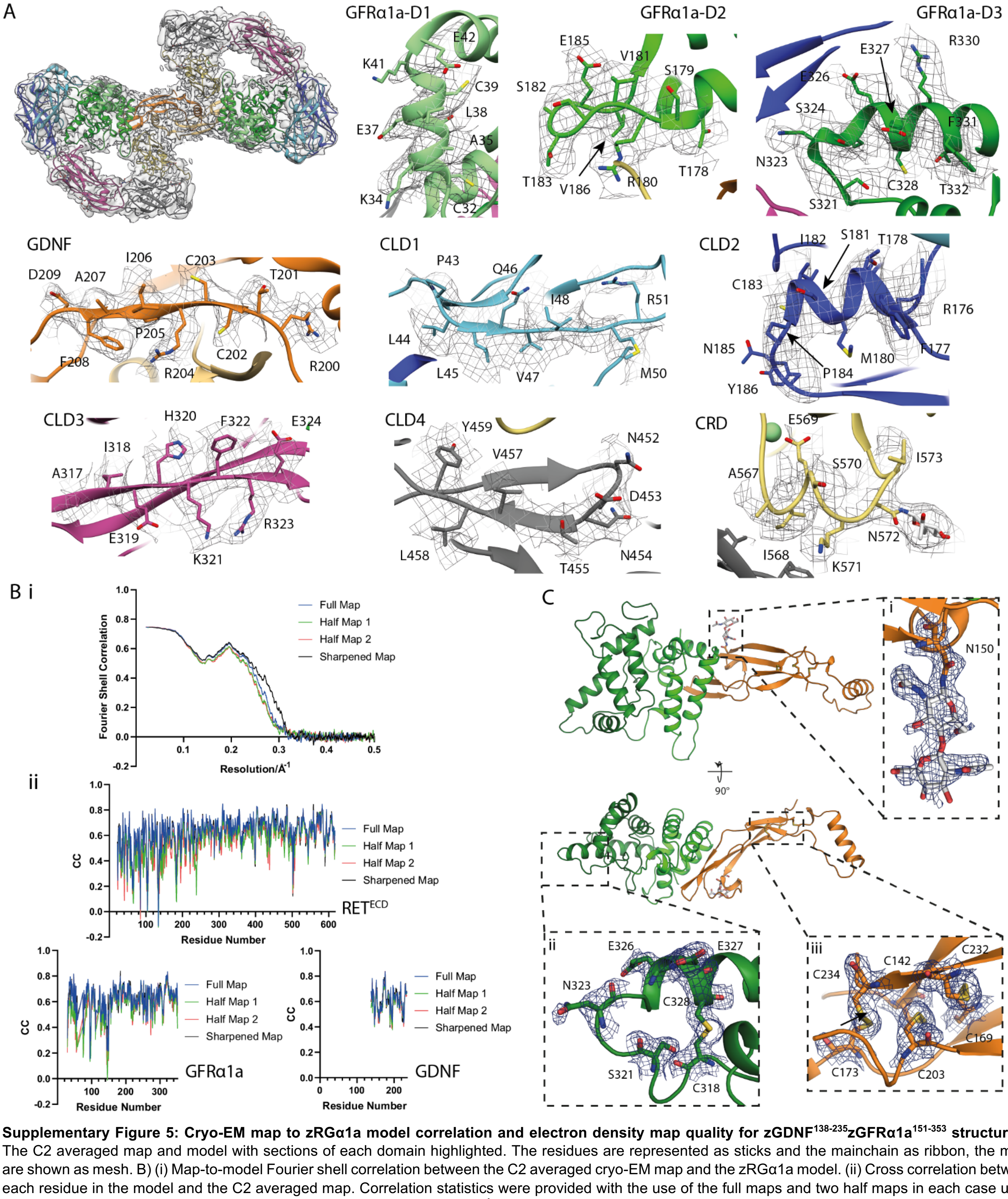

Supplementary Figure 5: Cryo-EM map to zRGα1a model correlation and electron density map quality for zGDNF$^{138\text{-}235}$zGFRα1a$^{151\text{-}353}$ structure A) The C2 averaged map and model with sections of each domain highlighted. The residues are represented as sticks and the mainchain as ribbon, the maps are shown as mesh. B) (i) Map-to-model Fourier shell correlation between the C2 averaged cryo-EM map and the zRGα1a model. (ii) Cross correlation between each residue in the model and the C2 averaged map. Correlation statistics were provided with the use of the full maps and two half maps in each case using Phenix cryo-EM model validation tools (Afonine et al., 2018). C) Final 2.2 Å zGDNF-zGFRα1a structure showing the crystallographic asymmetric unit contains a single copy of zGDNF$^{138\text{-}235}$zGFRα1a$^{151\text{-}353}$. The insets reveal the final electron density calculated for different areas of the structure using m2Fo-DFc coefficients and contoured at 1.0 σ. The glycosylation site located on N150 of GDNF (i), the binding site of zGFRα1a that interacts with RET CLD(2-3) calcium site (ii), and the disulfide bond network in zGDNF (iii). Images were rendered in Chimera (Pettersen et al., 2004) or PyMOL (Schrodinger, 2015).Related to Figure 2.

A

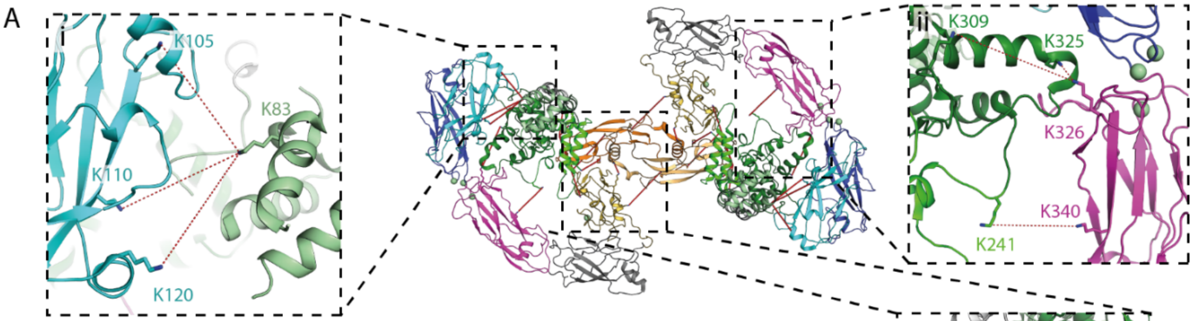

Score	Protein1	PepPos1	PepSeq1	LinkPos1	Protein2	PepPos2	PepSeq2	LinkPos2	
212.03	GDNF	192	KLDKDTPSR	1	RETECD	544	DKGISKR	2	
182.35	GFRa1	309	SPKISVSPFCDCSSSGNSKEECDR	3	RETECD	326	FREEKAIFGNVR	5	
169.05	RETECD	609	AGHGTCYCFEGKCFCER	12	GDNF	226	KHSAK	1	
163.62	GFRa1	325	ISVSPFCDCSSSGNSKEECDRFTEFFTDNACLR	16	RETECD	326	FREEKAIFGNVR	5	
155.38	GDNF	161	TKEELIFR	2	RETECD	544	DKGISKR	2	
147.34	RETECD	517	TDEPRFPACAEKR	12	GDNF	136	VKGQGR	2	*
140.5	RETECD	597	DLYGIKAGHGTCYCFEGK	6	GDNF	161	TKEELIFR	2	
127.49	GDNF	195	KLDKDTPSR	4	RETECD	511	FPACAEKR	7	
121.09	GFRa1	158	ENNCLNAAKACNLNDTCKK	9	GDNF	161	TKEELIFR	2	
118.13	GFRa1	83	LVLDALKQSPLYNCR	7	RETECD	105	SPKDLTLK	3	
118.11	GFRa1	149	LAPIYSGEPALAKENNCLNAAK	13	GDNF	161	TKEELIFR	2	
112.24	GDNF	225	TCCRPIAFDDDISFLDDSLEYHTLKK	25	GFRa1	191	RKCHK	2	
111.66	RETECD	120	VGVSSTPPMKVMCTILPTVEVK	10	GFRa1	83	LVLDALKQSPLYNCR	7	
99.02	GFRa1	48	ANELCLKEPGCSSKYR	14	GDNF	231	KCACV	1	#
92.69	GFRa1	241	RQTIVPACSYEDKERPNCLTLQVSCK	13	RETECD	340	GTVHEYKLK	7	
82.45	GFRa1	83	LVLDALKQSPLYNCR	7	RETECD	110	DLTLKVGVSSTPPMK	5	
79.74	GFRa1	34	LDCVKANELCLK	5	GDNF	136	VKGQGR	2	*

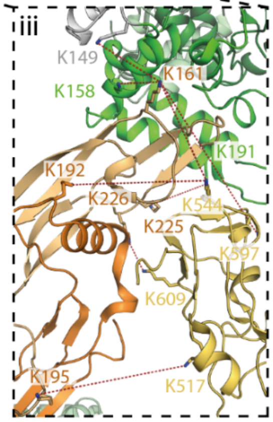

B

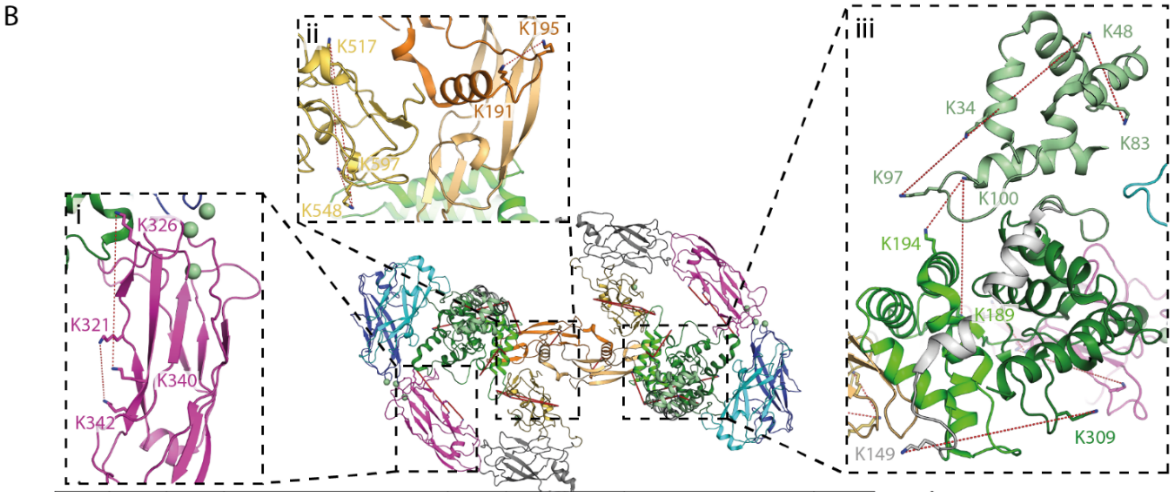

Score	Protein1	PepPos1	PepSeq1	LinkPos1	Protein2	PepPos2	PepSeq2	LinkPos2	
214.96	GDNF	195	KLDKDTPSR	4	GDNF	136	VKGQGR	2	*
171.33	GFRa1	189	VSTAEVCNKR	9	GFRa1	100	EKNCLR	2	
166.9	GFRa1	71	ESNFSMATGMEAKDECR	13	GFRa1	48	EPGCSSKYR	7	*
163.04	GDNF	161	TKEELIFR	2	GDNF	136	VKGQGR	2	*
159.98	GFRa1	309	SPKISVSPFCDCSSSGNSKEECDR	3	GFRa1	149	LAPIYSGEPALAKENNCLNAAK	13	
147.39	RETECD	326	FREEKAIFGNVR	5	RETECD	340	GTVHEYKLK	7	
144.03	GDNF	191	ILNNLTHNKK	9	GDNF	136	VKGQGR	2	*
133.6	RETECD	517	FPACAEKR	7	RETECD	548	DKGISKR	6	
119.14	GDNF	191	YCSGPCHDAETNYDKILNNLTHNKK	24	GDNF	195	LDKDTPSR	3	
118.75	RETECD	597	DLYGIKAGHGTCYCFEGK	6	RETECD	548	DKGISKR	6	
117.99	RETECD	342	LKLSQNLSVTEQR	2	RETECD	320	NNFAIEHKFR	8	
104	GFRa1	34	LDCVKANELCLK	5	GFRa1	97	RGMKK	4	
100.67	GFRa1	194	KCHKALR	4	GFRa1	100	EKNCLR	2	
99.02	GFRa1	47	ANELCLKEPGCSSKYR	14	GFRa1	83	LVLDALKQSPLYNCR	7	
98.46	RETECD	597	DLYGIKAGHGTCYCFEGK	6	RETECD	517	FPACAEKR	7	
88.56	GFRa1	71	ESNFSMATGMEAKDECR	13	GFRa1	83	LVLDALKQSPLYNCR	7	*
81.28	GFRa1	34	LDCVKANELCLK	5	GFRa1	47	EPGCSSKYR	7	
70.4	RETECD	340	GTVHEYKLK	7	RETECD	105	SPKDLTLK	3	

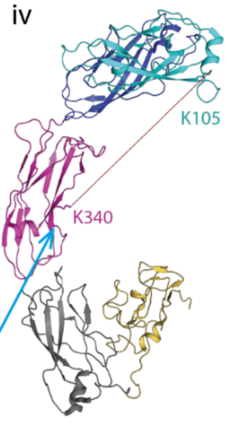

Supplementary Figure 6: Mapping interactions within the zRGα1a complex by XL-MS (Cross-Linking Mass Spectrometry). A) Intermolecular crosslinks identified between lysine side-chains from RET^{ECD}, $zGFR\alpha1a^{D1\text{-}D3}$ and $zGDNF^{mat.}$ highlighted in the C2 zRGα1a model, close-ups of the residues in the insets; RET^{CLD1}-$zGFR\alpha1a^{D1}$(i), RET^{CLD3}-$zGFR\alpha1a^{D3}$(ii) and RET^{CRD}-$zGFR\alpha1a^{D2}$-$zGDNF^{mat}$ (iii). B) Intramolecular crosslinks between lysine side-chains from RET^{ECD}, $zGFR\alpha1a^{D1\text{-}D3}$ and $zGDNF^{mat.}$ highlighted in the C2 zRGα1a model, close-ups of the residues in the insets; RET^{CLD3} (i), RET^{CRD}/ $zGDNF^{mat.}$ (ii) $zGFR\alpha1a^{D1\text{-}D3}$ (iii). (iv) Shows an intramolecular crosslink between CLD1 and CLD3 indicating flexibility at the calcium binding site. The crosslinked peptides highlighted with * do not have any structural model therefore are not shown in the model. The crosslinked peptide highlighted with # represents domain 1 of zGFRα1a cross-linked to zGDNF which has a distance of ~59 Å. This may indicate that $zGFR\alpha1a^{D1}$ is quite mobile consistent with the poorer quality of the map for this domain. The overall C2 zRGα1a model is represented as a cartoon with the crosslinks formed between lysines using disuccinimidyl sulfoxide (DSSO) represented as red lines between crosslinked lysines represented as sticks. The model is coloured according to its domains; CLD1 in cyan, CLD2 in blue, CLD3 in magenta, CLD4 in grey, CRD in yellow, $zGFR\alpha1a^{D1}$ in pale green, $zGFR\alpha1a^{D2}$ in green, $zGFR\alpha1a^{D3}$ in dark green and zGDNF in orange. All images were rendered in PyMOL (Schrodinger, 2015). Related to STAR methods.

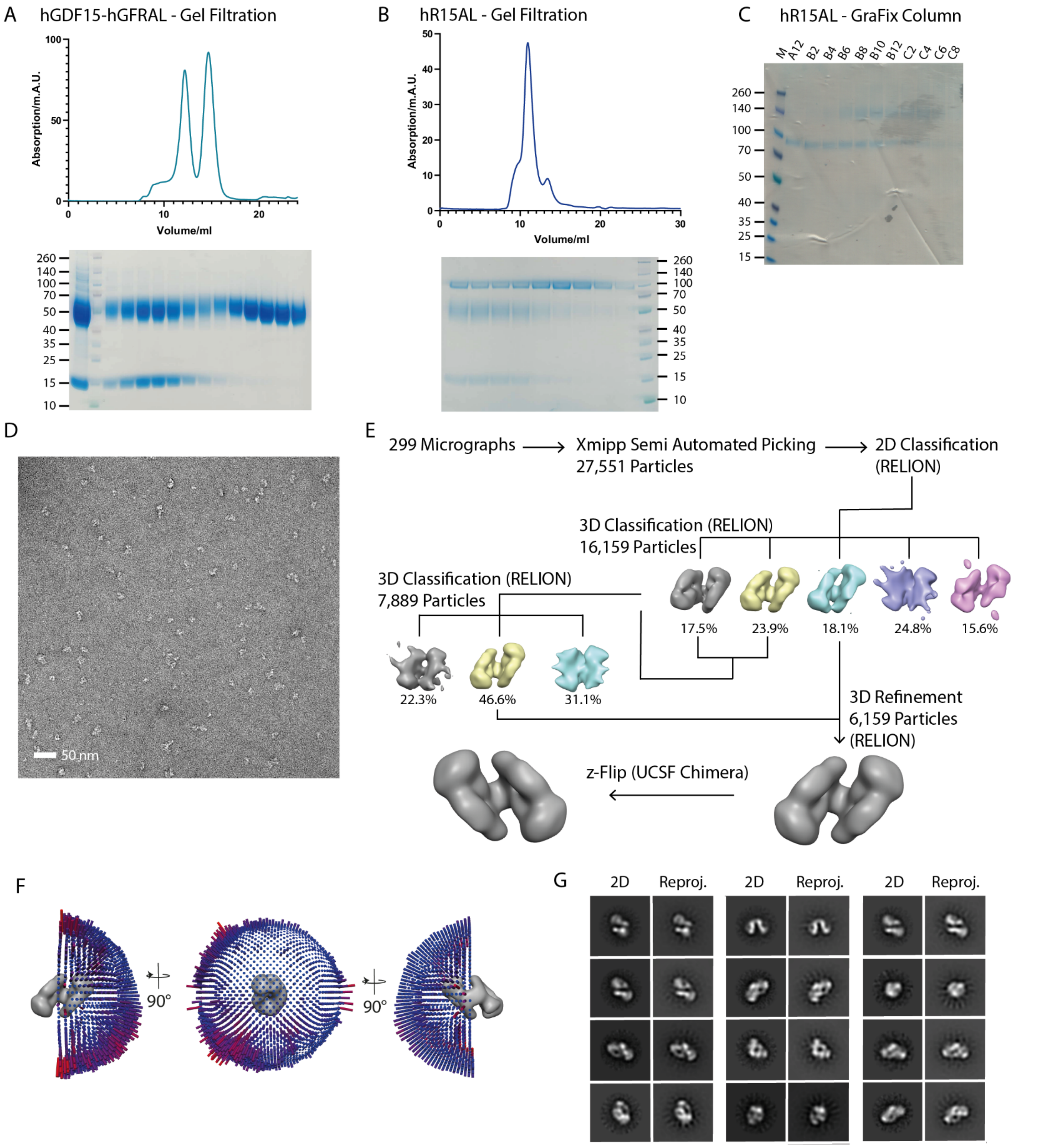

Supplementary Figure 7: hR15AL sample preparation and EM data processing. Size exclusion profiles and Coomassie-stained SDS-PAGE gels of A) hGDF15-hGFRAL and B) hRET-hGDF15-hGFRAL (hR15AL). C) fractions from the GraFix (Gradient Fixation) of hR15AL. D) A representative negative stain micrograph of the hR15AL-XL complex. E) The data processing pipeline leading to the final negative stain envelope of hR15AL. F) The particle distribution in the negative stain envelope with C2 symmetry applied. G) Projection matching, performed using Xmipp projection match (De la Rosa-Trevín et al., 2013), between the RELION (Kimanius et al., 2016; Scheres, 2012; Zivanov et al., 2018) showing 2D class averages from the particles that comprise the final reconstruction and different views of the 3D envelope. Related to Figure 6.

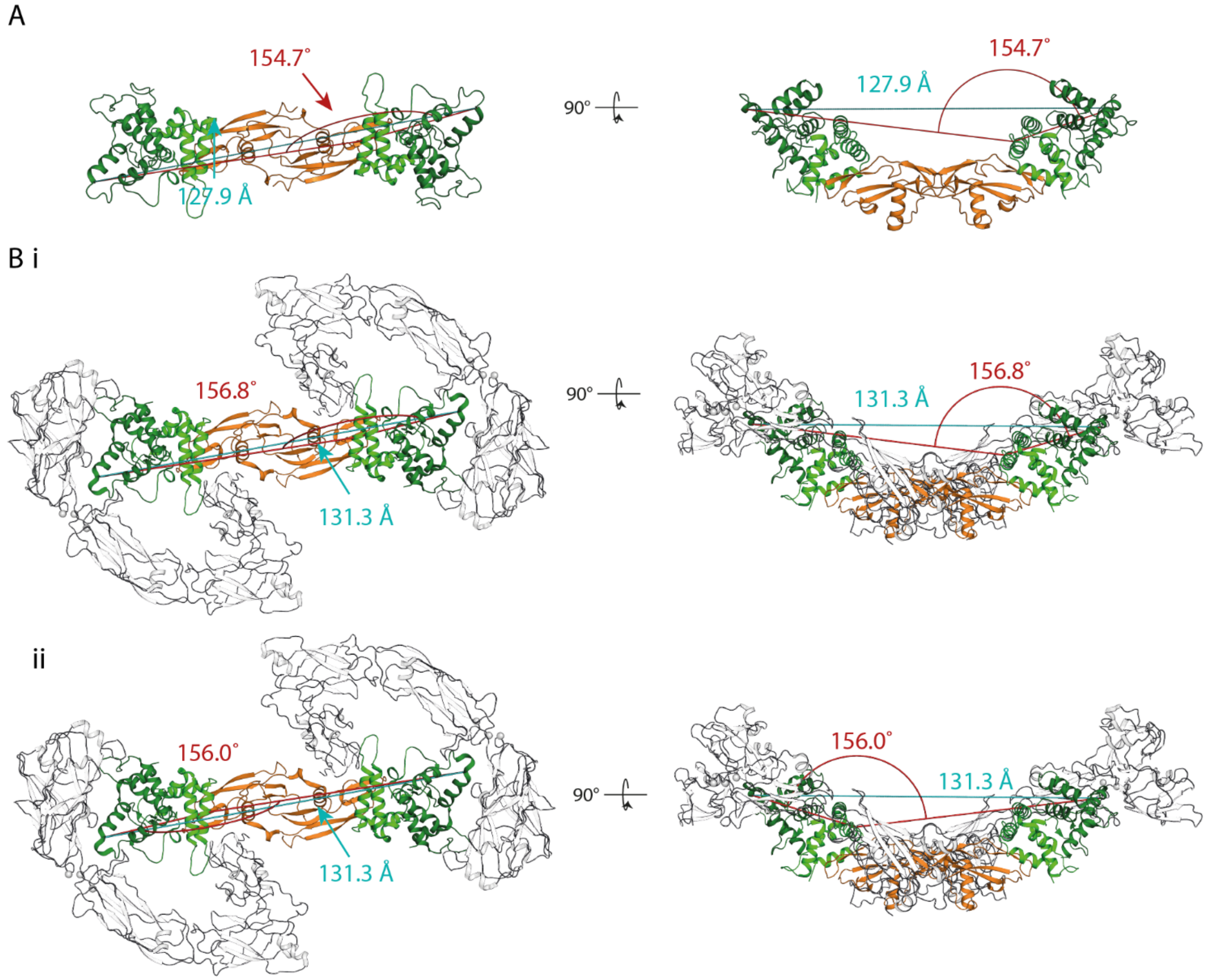

Supplementary Figure 8: Evidence for limited conformational flexing of zGDNF-zGFRα1a in the presence or absence of zRET^{ECD}. A) The crystal structure of a 2:2 zGDNF-zGFRα1a is shown as a cartoon, with GDNF in light orange, zGFRα1a^{D2} in light green and zGFRα1a^{D3} in green. The distance between K325 from symmetry-related molecules of zGFRα1a is highlighted in cyan and the angle between the K325-A172 from one molecule of zGFRα1 and K325 in the second molecule of zGFRα1a in red. B) 2:2 ligand:co-receptor zGDNF-zGFRα1a^{D2-D3} built into the cryo-EM zRGα1a structure, represented as a cartoon, with zGFRα1a^{D2}, zGFRα1a^{D3} and zGDNF in green, forest green and orange, respectively, and zRET^{ECD} in grey. The distance between K325 from symmetry-related molecules of zGFRα1a highlighted in cyan and the angle between the K325-A172 from one molecule of zGFRα1 and K325 in the second molecule of zGFRα1a in red, there are two angles calculated using these residues in the C2 averaged structure highlighted in parts (i) and (ii) of the figure. All images were rendered in PyMOL (Schrodinger, 2015). Related to Figure 4.

SUPPLEMENTARY TABLES

	LIGAND				CO-RECEPTOR				RET	
	zGDNF	hGDNF	hNRTN	hGDF15	zGFRα1	hGFRα1	hGFRα2	hGFRAL	zRET	hRET
**LIGAND**										
zGDNF	1530Å				866Å				347Å	
zGDNF*									251Å	
hGDNF		1003Å				961Å				187Å
hGDNF*										148Å
hNRTN			2006Å				843Å			296Å
hNRTN*										218Å
hGDF15				1398Å				577Å		407Å
hGDF15*										369Å
**CO-RECEPTOR**										
zGFRα1									846Å	
hGFRα1										872Å
hGFRα2										960Å
hGFRAL										1094Å

Interface sizes (averaged over both protomers) calculated by PDBePISA

* Contact surface to the second protomer of GFL dimer

Supplementary Table 1: Major interface sizes for ternary complexes of hRET/zRET, GFRα1/α2/GFRAL and GDNF/NRTN/GDF15. Related to STAR methods.

SUPPLEMENTARY DOCUMENT S1

XL-MS analysis of the zRET^{ECD}-GFRα1-GDNF complex:

All chemicals were purchased from Sigma at the highest purity unless otherwise stated. A total of 200 ng protein in 20 mM HEPES (pH 7.5), 150 mM NaCl, 1 mM $CaCl_2$ was cross-linked using 1 mM disuccinimidyl sulfoxide (DSSO)(Kao et al., 2011) (Thermo Fisher Scientific) with mild shaking for 15 min at 37 °C. The reaction was quenched using a final concentration of 5% hydroxylamine for a further 15 min at 37 °C. The sample was subsequently alkylated, reduced and proteolysed. To do this, the sample was dried to completion using vacuum centrifugation and resolubilised with sonication into 8 M urea. Cysteine reduction was carried out using 2.5 mM TCEP for 30 min at 37 °C and alkylated in the dark using 5 mM iodoacetamide at room temperature for 30 min. The urea was diluted to 1 M using 50 mM triethylammonium bicarbonate and proteins were proteolysed using trypsin (Pierce) at 1:50 w/w trypsin:protein overnight at 37 °C. The solution was acidified to pH 2-3 using trifluoroacetic acid (TFA) and desalted using in house built STAGE tips made using Empore SPE C18 disks (3M, 66883-U). The eluent was then dried to completion.

Liquid Chromatography Mass Spectrometry

Peptides were reconstituted in 0.1 % TFA (v/v) and chromatographically resolved using an Ultimate 3000 RSLCnano (Dionex) HPLC. Peptides were first loaded onto an Acclaim PepMap 100 C18, 3 µm particle size, 100 Å pore size, 20 mm x 75 µm ID (Thermo Scientific, 164535) trap column using a loading buffer (2 % acetonitrile (MeCN) (v/v) and 0.05 % TFA in 97.95 % H2O) with a flow rate of 7 µL/min. Chromatographic separation was achieved using an EASY-Spray column, PepMap C18, 2 µm particles, 100 Å pore size, 500 mm x 75 µm ID (Thermo Scientific, ES803). The gradient utilised a flow of 0.275 µL/min, starting at 98 % mobile A (0.1% formic acid, 5 % dimethyl sulfoxide (DMSO) in H_2O) and 2 % mobile B (0.1 % formic acid, 75 % MeCN, 5% DMSO and 19.9 % H2O). After 3 min mobile B was increased to 8 % over 3 min, increased to 25 % over 69 min, to 45 % over 35 min, further increased to 90% in 17 min and held for 5 min. Finally, mobile B was reduced back to 5 % over 3 min for the rest of the acquisition.

MS1 data were acquired in real time over 150 minutes using an Orbitrap Fusion Lumos Tribrid mass spectrometer in positive, top speed mode with a cycle time of 5 s. The chromatogram (MS1) was captured using 60,000 resolution, a scan range of 375-1500 with a 50 ms maximum injection time, and 4e5 AGC target. MS2 dynamic exclusion with repeat count 2, exclusion duration of 30 s, 20 ppm tolerance window was used, along with isotope exclusion, a minimum intensity exclusion of 2e4, charge state inclusion of 3-8 ions and peptide mono isotopic precursor selection. Precursors within a 1.2 *m/z* isolation window were then fragmented using 25 % normalised collusion-induced dissociation (CID), 100 ms maximum injection time and 5 e4 AGC target. Scans were recorded using 30,000 resolution in centroid mode starting 120 *m/z*. MS3 spectra containing peaks with a mass difference of 31.9721 Da were further fragmented with a 43 % normalised higher collision induced dissociation, using a 2 *m/z*

isolation window, 150 ms maximum injection time and 2e4 AGC target. 4 scans were recorded using an ion trap detection in rapid mode starting at 120 *m/z*.

Data analysis.

Data processing was carried out using Proteome Discoverer Version 2.3 (ThermoFisher Scientific) with the XlinkX node(2017; Liu et al., 2015). The acquisition strategy was set to MS2_MS3 mode. The database comprised solely of the specific zRET^{ECD}, zGFRα1a^{D1-3} and GDNF$^{mat.}$ sequences. Trypsin was selected as the proteolytic enzyme allowing up to two missed cleavages with a minimal peptide length of five residues. Masses considered were in the range of 0.3-10 kDa. The precursor mass tolerance, FTMS fragment mass tolerance, and ITMS Fragment Mass Tolerance were set to 10 ppm, 20 ppm and 0.5 Da respectively. A static carbamidomethyl (+57.021 Da) modification was utilised for cysteine residues, with an additional dynamic modification for oxidation (+15.995 Da) on methionine residues. The False Discovery Rate (FDR) threshold was set to 0.05 with percolator as the strategy. The list of reported cross-linked spectral matches were manually examined and cross-links with spectra that did not contain acceptable b and y ion coverage were excluded. The reduced list was exported to crosslinkviewer.org (Combe et al., 2015) in order to graphically view the cross-links.
